# Food Grade Synthesis of Hetero-Coupled Biflavones and 3D-Quantitative Structure–Activity Relationship (QSAR) Modeling of Antioxidant Activity

**DOI:** 10.3390/antiox14060742

**Published:** 2025-06-16

**Authors:** Hongling Zheng, Xin Yang, Qiuyu Zhang, Joanne Yi Hui Toy, Dejian Huang

**Affiliations:** 1 Department of Food Science and Technology, National University of Singapore, 2 Science Drive 2, Singapore 117542, Singapore; e0035606@u.nus.edu (H.Z.); zhangqy@nus.edu.sg (Q.Z.); 2 David H. Koch Institute for Integrative Cancer Research, Massachusetts Institute of Technology, Cambridge, MA 02139, USA; xin_yang@mit.edu; 3 Biomedical and Health Technology Platform, National University of Singapore (Suzhou) Research Institute, 377 Linquan Street, Suzhou 215123, China

**Keywords:** biflavones, oxidative coupling, antioxidant activity, QSAR

## Abstract

Biflavonoids are a unique subclass of dietary polyphenolic compounds known for their diverse bioactivities. Despite these benefits, these biflavonoids remain largely underexplored due to their limited natural availability and harsh conditions required for their synthesis, which restricts broader research and application in functional foods and nutraceuticals. To address this gap, we synthesized a library of rare biflavonoids using a radical–nucleophile coupling reaction previously reported by our group. The food grade coupling reaction under weakly alkaline water at room temperature led to isolation of 28 heterocoupled biflavones from 11 monomers, namely 3′,4′-dihydroxyflavone, 5,3′,4′-trihydroxyflavone, 6,3′,4′-trihydroxyflavone, 7,3′,4′-trihydroxyflavone, diosmetin, chrysin, acacetin, genistein, biochanin A, and wogonin. The structures of the dimers are characterized by nuclear magnetic resonance spectroscopy (NMR) and high-resolution mass spectroscopy (HRMS). In addition, we evaluated the antioxidant potential of these biflavones using a DPPH (2,2-diphenyl-1-picrylhydrazyl) radical scavenging assay and the DPPH value ranges between 0.75 to 1.82 mM of Trolox/mM of sample across the 28 synthesized dimers. Additionally, a three-dimensional quantitative structure–activity relationship (3D-QSAR) analysis was conducted to identify structural features associated with enhanced antioxidant activity. The partial least squares (PLS) regression QSAR model showed acceptable r^2^ = 0.936 and q^2^ = 0.869. Additionally, the average local ionization energy (ALIE), electrostatic potential (ESP), Fukui index (F-), and electron density (ED) were determined to identify the key structural moiety that was capable of donating electrons to neutralize reactive oxygen species.

## 1. Introduction

**Figure 1:**
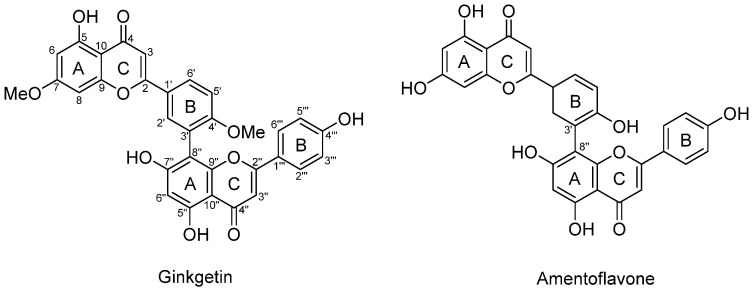
Molecular structures of ginkgetin and amentoflavone.

Flavonoids are common secondary metabolites found in plants and they consist of a 15-carbon skeleton which includes two phenyl rings (A and B) and a heterocyclic ring (C). Two flavonoid monomers are connected via different interflavonyl linkages (A–A, A–B, A–C, B–B, B–C, and C–C) to form biflavonoids, which are an important class of secondary metabolites with a broad spectrum of health promotion activities which include antioxidant [1,2], anti-inflammatory [3,4], antiviral [5,6], antibacterial [7,8], antifungal [9], cytotoxic [10,11,12], and neuroprotective properties [13,14]. Despite their promising bioactivities, the chemical space of biflavonoids remains largely underexplored due to their limited natural abundance. Biflavonoids are found in plant angiosperms of *Clusiaceae*, *Thymelaeaceae*, *Ochnaceae*, and *Selaginellaceae*, accounting for about half of all the biflavonoids reported so far [15]. The most studied biflavonoids are ginkgetin and amentoflavone (Figure 1), as they are found in a great variety of plant species [16,17]. Biflavonoids can also be synthesized chemically via Suzuki–Miyaura [18] or Ullmann coupling [19] in the presence of metal catalysts with pre-functionalized flavonoid monomers with the phenolic group protected. However, the multi-step chemical synthesis often involves harsh conditions, which limits the discovery and production of new biflavonoids. Our lab has reported a simple method for synthesizing biflavones [20]. The method features commercially available flavonoids without any prior chemical modifications and a one-step catalyst-free coupling reaction conducted under an ambient, food-grade alkaline condition. This green and versatile method opens the door to obtaining rare biflavonoids with ease. It expands the current library of AB-type biflavonoids, which predominantly consists of amentoflavone [15] and its derivatives with C3′–C8″ linkage by introducing biflavonoids with C2′–C6″ linkage, thereby enhancing the structural diversity.

Aerobic respiration generates energy as well as free radicals in the form of reactive oxygen species (ROS) [21] or reactive nitrogen species (RNS). The accumulation of a large number of free radicals leads to oxidative stress, which has been linked to the development of various chronic diseases, including cancer and pathological inflammation [22]. The oxidative stress can be counteracted by antioxidants such as biflavonoids [23]. This library of biflavonoids, which share the same C–C linkage but differ in the position and types of substituent groups, serves as an excellent basis for developing three-dimensional quantitative structure–activity relationships (QSARs). Thus, we built a QSAR model to analyze the antioxidant activity of biflavonoids based on experimental results from the DPPH assay. This model elucidates the antioxidant effects of these rare biflavonoids and aids in the design of biflavonoids with improved antioxidant potential, thereby contributing to the development of bioactive compounds with therapeutic applications.

## 2. Materials and Methods

Materials: Potassium hydroxide was obtained from Merck & Co., Inc. (Rahway, NJ, USA). Flavones (3′,4′-dihydroxyflavone, 5,3′,4′-trihydroxyflavone, 6,3′,4′-trihydroxyflavone, 7,3′,4′-trihydroxyflavone) were obtained from Indofine Chemical Co., Inc., Hillsborough, NJ, USA. Diosmetin, chrysin, acacetin, genistein, biochanin A, wogonin, and chrysoeriol were obtained from BLD Pharmatech Ltd., Shanghai, China. In addition, 2,2-diphenyl-1-picrylhydrazyl (DPPH) and 6-hydroxy-2,5,7,8-tetramethylchloroman-2-carboxylic acid (Trolox) were purchased from Sigma Chem (Burlington, MA, USA). Dimethylsulfoxide (DMSO) was purchased from Thermo Fisher Scientific Co., Ltd. (Waltham, MA, USA). Additionally, 96-Well TC-Treated Microplates were purchased from Corning Inc. (Corning, NY, USA). All aqueous solutions were prepared with 18.2 MΩ·cm ultrapure water obtained from a Millipore water purification system (Merck KGaA, Darmstadt, Germany).

Reaction mixtures were analyzed by a Waters high-performance liquid chromatography (HPLC) system with a C18 column (Phenomenex, Torrance, CA, USA, Luna 5 μm C18, 250 × 4.6 mm) and PDA detector at 300 nm. ^1^H and ^13^C NMR spectra were recorded on Bruker (Billerica, MA, USA) 500 MHz spectrometers. TopSpin 4.0.5 was used for NMR data collection and MestReNova v12.0.0 was used for NMR data analysis. Chemical shifts are reported in ppm from the solvent resonance as the internal standard: ^1^H (DMSO-*d6*: δ 2.50 ppm), ^13^C (DMSO-_d6_: δ 39.52 ppm). Data are reported as follows: chemical shift (δ ppm), integration, multiplicity (s = singlet, d = doublet, t = triplet, q = quartet, m = multiplet), and coupling constants (Hz).

### 2.1. Synthesis of Biflavonoids

Flavones as radical precursors (0.25 mmol, 1.0 eq) and flavones as nucleophiles (0.3125 mmol, 1.25 eq) were added to an alkaline solution (125 mL of 0.01 M KOH) in a 500 mL plastic bottle, after which the pH was adjusted to 11.5 using 0.1 M KOH. The bottle was capped and kept at room temperature without stirring. The progress of the reaction was monitored using HPLC. Concentrated hydrochloric acid (4 mL, 10 M) was added to the reaction mixture to quench the reaction. The mixture was then extracted using ethyl acetate (2 × 125 mL). The combined organic phases were evaporated in vacuo. The dried organic phase was dissolved in methanol and filtered using a 0.22 µm membrane (Merck Millipore, Burlington, MA, USA) for HPLC analysis and separation.

### 2.2. HPLC Separation of Reaction Mixtures

A gradient elution method was applied for the separation and characterization of flavonoid coupling products. DI water with 0.1% formic acid was used as mobile phase A and acetonitrile (ACN) was used as mobile phase B. The column was conditioned with 95% of mobile phase A for 10 min before gradient elution from 95% of mobile phase A to 100% of mobile phase B in 30 min at a flow rate of 1.0 mL/min. The Waters 2998 Photodiode Array (PDA) Detector was used with detection wavelengths from 190 to 800 nm. The signal at 300 nm was analyzed. Isocratic elution with DI water and acetonitrile was used for the isolation of biflavonoids. The elution condition for each biflavonoid can be found in the Supplementary Materials.

### 2.3. Characterization of Biflavonoids with ^1^H NMR, ^13^C NMR, and MS

The ^1^H NMR and ^13^C NMR spectra for the various biflavonoids were obtained using DMSO-*d_6_* (350–500 µL) as the solvent and recorded on a Bruker AVANCE I 500 NMR spectrometer. Additionally, two-dimensional NMR techniques, including HSQC and HMBC, were also employed. Mass spectrometry was performed on a Thermo Scientific (Waltham, MA, USA) LCQ Fleet ion trap mass spectrometer (MS) in ES negative mode. The temperature of the heated capillary was set to 250 °C, and the spray voltage was maintained at 4.5 kV. Nitrogen served as the sheath gas at a flow rate of 80 psi, while the auxiliary gas flow rate was set at 20 psi. Full-scan mass spectra were recorded in negative ion mode across a mass-to-charge ratio (*m*/*z*) range of 100–1000, with a scanning speed of one scan per second. High-resolution electrospray ionization mass spectrometry (HRESIMS) was conducted using an Agilent 6546 Accurate-Mass LC-QTOF-MS instrument in negative ion mode over a mass-to-charge ratio (*m*/*z*) range of 100–1000, with a scan speed of one scan per second.

### 2.4. DPPH Assay

A previously reported DPPH method was modified to evaluate the radical scavenging activity of the biflavonoid [24]. The stock solutions (20 mM) of each sample were prepared by dissolving the biflavones in DMSO. The stock solutions were diluted to 0.125 mM using MeOH: DI water (20:80 *v*/*v*). The Trolox standard (1 mM) was freshly prepared and serially diluted to generate a calibration curve over the concentration range of 0.125 mM to 0.001 mM. The 0.5 mM DPPH solution was prepared by dissolving 1.95 mg DPPH in 10 mL methanol. Each well on the 96-well plate was loaded with 30 µL sample and 30 µL of 0.5 mM DPPH solution. After 30 min, the absorbance at 517 nm was recorded on a Biotek Epoch 2 microplate spectrophotometer equipped with BioTek Gen5 software version 3.02. Antioxidant activity of each biflavonoid was evaluated using the standard calibration curve of the Trolox solution and the final readings were reported as mM of Trolox equivalent per mM of sample.

### 2.5. Data Set Preparation, Alignment, and 3D-QSAR Modeling by Partial Least Squares (PLS) Analysis

A dataset comprising 28 synthesized dimers was utilized to investigate the antioxidant activity, specifically the DPPH radical scavenging activity (expressed in mM of Trolox/mM of dimer). The 2D structures of the ligands were extracted from an SDF file and subsequently converted into 3D using the LigPrep tool in Schrodinger Suite [25]. LigPrep was employed to generate the lowest-energy conformations of the ligands while preserving the stereochemistry and protonation states at the physiological pH. The OPLS4 force field was applied to generate the 3D structure. Subsequently, energy minimization was performed using the Minimization tool in Maestro to further optimize the geometry of the 3D conformers, under the OPLS4 force field with a convergence criterion of 0.01 kcal/mol/Å for the gradient. After the minimization process, the ligands were aligned using Ligand Alignment based on a pharmacophore model. The aligned structures were then utilized for field-based QSAR. The field-based 3D-QSAR models were developed using the PHASE module of Schrödinger’s software (https://www.schrodinger.com/platform/products/phase/, accessed on 8 June 2025). Partial least squares (PLS) analysis was employed to develop 3D-QSAR models, with DPPH values as dependent variables and Gaussian intensities as descriptors (independent variables). Various statistical parameters such as standard deviation (SD), non-cross-validated correlation coefficient (R^2^), cross-correlation validation (R^2^CV), R^2^ scramble, Fischer’s test (F-test), variance ratio (*p*-value), root mean square error (RMSE), Pearson r-values, correlation coefficient of external validation (Q^2^), and Pearson r were computed to assess the reliability of the established models. Partial charges were considered when the potential fields were calculated and the grid spacing was set as 1.0 Å. The number of ligands to leave out for the cross-validation was set as 1 (leave-one-out). In order to evaluate the predictive ability of the developed QSAR models, the dataset was randomly split into a training and test set (80:20). The maximum PLS factor was set as 4 to avoid over-fitting of the models and the best 3D-QSAR model was selected based on statistical analysis of the internal and external validation parameters. Lastly, the field-based models were represented as steric, electrostatic, hydrophobic, hydrogen bond donor, and hydrogen bond acceptor.

### 2.6. Density Functional Theory (DFT) Calculation

The molecular orbitals of the compounds and atomic energy features were determined via Becke’s three-parameter exchange potential and the Lee–Yang–Parr correlation (B3LYP) density functional theory approach utilizing 6–31G** as the basis set [26]. The jaguar panel of Maestro (Schrodinger Suite) was used to determine the different quantum mechanical parameters of the initially optimized compound. The calculations included the determination of the following properties: highest occupied molecular orbital (HOMO), lowest unoccupied molecular orbital (LUMO), their energy gap (ΔE), electrostatic potential (ESP), average local ionization energy (ALIE), Fukui indices (F-), and electron density (ED).

## 3. Results and Discussion

### 3.1. Synthesis of Biflavonoids Under Alkaline Conditions

**Figure 2:**
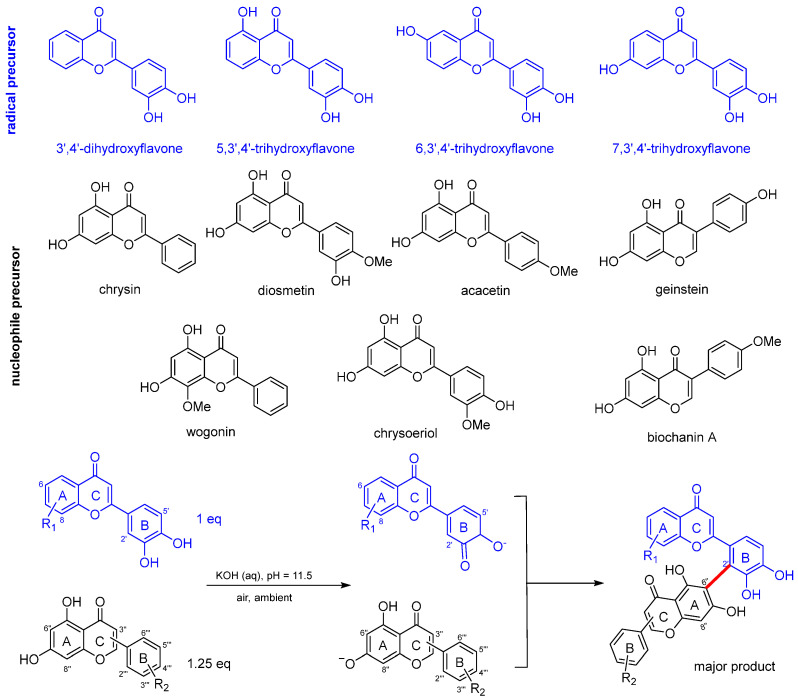
Molecular structures of the flavonoid monomers and reaction scheme for biflavone formation in alkaline solution. [blue molecules represent radical precursor, black molecules represent nucleophile precursors and the red bond indicates the new bond formed].

The experiments were conducted at room temperature in the aeriated buffer at pH 11.5 to isolate sufficient quantities of product for subsequent characterization and biological assays. The reaction scheme is shown in Figure 2 and the mechanisms were proposed based on our previous study [20]. The stoichiometric ratio was adjusted to 1:1.25, with 1.25 equivalents of the nucleophile precursor used to shift the equilibrium of this reversible reaction toward product formation. This occurs under alkaline conditions, and the flavones first undergo deprotonation to form phenolates which are subsequently converted to their respective ortho-semiquinone anion radicals by molecular oxygen dissolved in the buffer. These radicals then react with flavone nucleophiles generated by deprotonation under the alkaline condition, leading to the formation of biflavones. The reaction is therefore pH-sensitive and requires conditions that promote deprotonation of the coupling partners. Two biflavones with C2′–C6″ and C2′–C8″ linkages were formed, the one featuring the C2′–C6″ linkage being the major product due to the lower averaged local ionization energy at C6″ [20]. The coupling reaction gave rise to one major regioisomer and one (or more) minor isomers, which were not sufficient to be isolated for further characterizations. Therefore, we focused on the isolation of the major isomers that have C2′–C6″ interflavonyl linkage.

Due to the reversible nature of the coupling reactions, careful monitoring of reaction progress is essential to maximize product formation and minimize product degradation. For future efforts aimed at reproducing or scaling up the synthesis of specific biflavonoids, optimization of key parameters such as reaction time, stoichiometric ratio, and reagent concentration is crucial for improving product yield.

**Figure 3:**
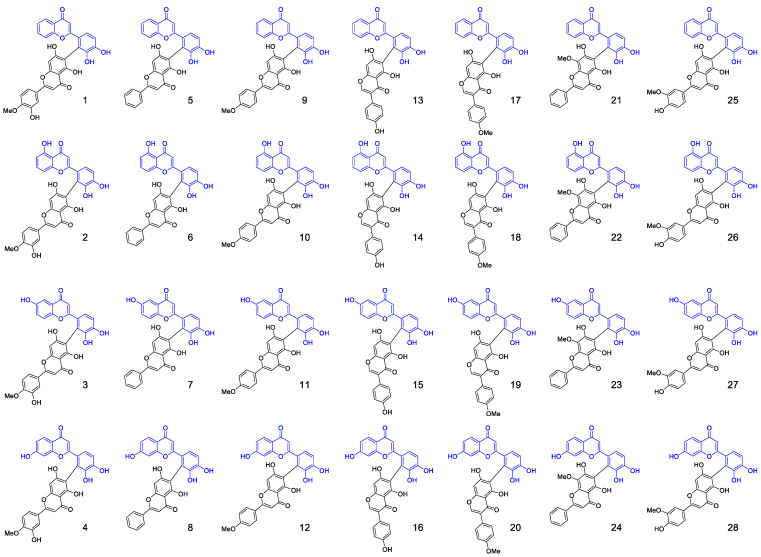
Structures of the 28 rare biflavonoids.

We were able to isolate 28 biflavones from radical precursors (3′,4′-dihydroxyflavone, 5,3′,4′-trihydroxyflavone, 6,3′,4′-trihydroxyflavone, 7,3′,4′-trihydroxyflavone) and various nucleophiles (diosmetin, chrysin, acacetin, genistein, chrysoeriol, biochanin A, and wogonin). The structures of these biflavones are listed in Figure 3. Radical precursors are selected to diversify the biflavone library as the positions and number of substituents on ring A of these flavonoids differ from the monomers that constitute natural biflavones with C2′–C6″ linkage. Naturally occurring biflavones contain two substituents on ring A, specifically either one hydroxyl and one methoxy substituent or two hydroxyl group at positions C5 and C7 [15]. There is no hydroxyl substituent on ring A of 3′,4′-dihydroxyflavone and there is only one hydroxyl substituent on 5,3′,4′-trihydroxyflavone, 6,3′,4′-trihydroxyflavone, and 7,3′,4′-trihydroxyflavone. Flavones bearing hydroxyl groups at positions C5 and C7 were chosen as the second monomer because their deprotonated forms are sufficiently nucleophilic to undergo radical coupling reactions for biflavone synthesis.

### 3.2. HPLC Analysis of the Reaction Profiles

**Figure 4:**
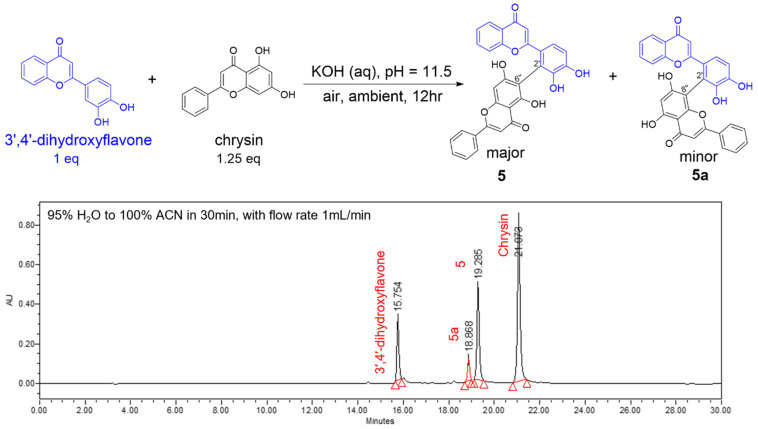
Reaction scheme between 3′,4′-dihydroxyflavone and chrysin. HPLC chromatogram of the reaction.

HPLC analysis was performed in detail for a representative reaction between 3′,4′-dihydroxyflavone and chrysin to form the dimer designated as biflavone **5**, in order to provide insight into product distribution. Based on mass spectrometry data, this reaction yielded two biflavonoid isomers (Figure 4). According to the previously reported reaction mechanism, the major biflavone contains C2′–C6″ linkage while the minor isomer contains C2′–C8″ linkage [20]. Biflavones **5** and **5a** were isolated and characterized using ^1^H and ^13^C NMR and mass spectroscopy. Compounds **5** and **5a** have *m*/*z* values of 505.0929 and 505.0930, respectively. However, different proton and carbon signals were observed in the 1D and 2D NMR spectra of the isomers (Figures S5 and S30–S32). In the chromatogram of wogonin (Figure S1), only three peaks were observed because wogonin has a methoxy group instead of hydrogen at C8, resulting in the formation of only one product. The yield of each major dimer is reported in the Supplementary Materials.

### 3.3. Separation and Characterization of Biflavones

The isolations were performed using semi-prep HPLC. While the crude products were more soluble in methanol compared to acetonitrile, using a water–methanol mobile phase combination generated high pressure that often disrupted the flow. Thus, acetonitrile was selected as the organic mobile phase solvent instead. The mobile phase composition (water:acetonitrile ratio) was adjusted to ensure proper peak separation. Although increasing the water content improved peak resolution, it correspondingly increased the separation time. Thus, optimization involved balancing peak resolution with analysis duration to establish appropriate separation conditions. Gradient elution was adopted initially with the intention to shorten the separation time. However, it gave poor peak resolution and thus isocratic elution was used. The flow rate was kept at 5 mL/min to ensure fast separation and stable pressure for smooth separation. The separation conditions as well as the ^1^H and ^13^C NMR are reported in the Supplementary Materials (Figures S2–S29). The assignments of signals in ^1^H and ^13^C NMR spectra are reported in Tables S1 and S2.

### 3.4. Antioxidant Activity Measured by DPPH Assay

**Figure 5:**
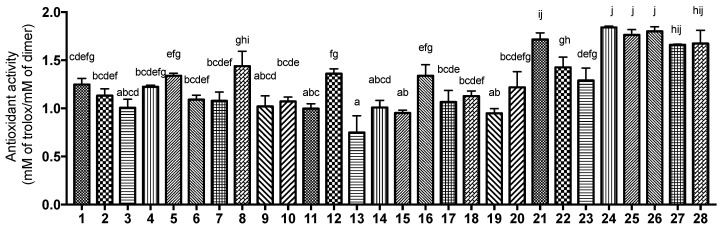
DPPH activity of the biflavonoids **1**–**28**. a–j: Bar represented by different letters means DPPH values are significantly different (*p* < 0.05) by one-way ANOVA with post hoc Tukey’s test.

The antioxidant activities of the dimers were determined using a DPPH assay and the results obtained are shown in Figure 5. Trolox was utilized as the positive control and the calibration curve for Trolox follows a linear regression with an R^2^ value of 1.00. The antioxidant activity was expressed as mM of Trolox/mM of sample. The DPPH value ranges between 0.75 to 1.82 mM of Trolox/mM of sample across the 28 synthesized dimers. In particular, biflavonoids **21** and **24**–**28** had a statistically higher antioxidant activity as compared to the rest of the samples. Flavonoids are recognized for their ability to neutralize ROS and RNS by donating a hydrogen atom and an electron to radicals such as hydroxyl, peroxyl, and peroxynitrite [27]. This process stabilizes the radicals, resulting in the formation of a comparatively stable flavonoid radical.

### 3.5. Field-Based 3D-QSAR Analysis

**Figure 6:**
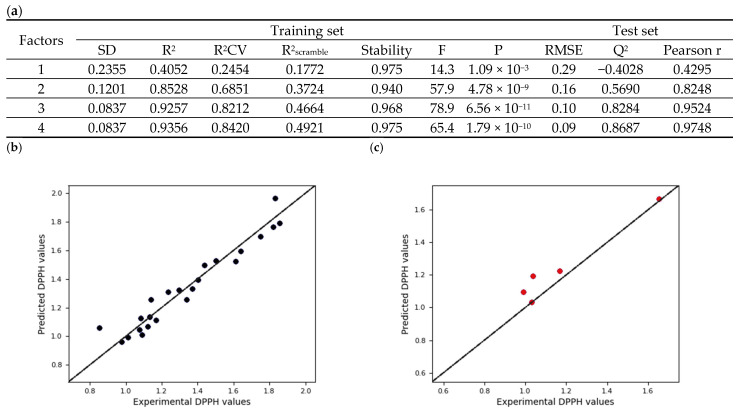
(**a**) Partial least squares (PLS) statistics for field-based 3D-QSAR model and regression plot of experimental vs. predicted DPPH values for the (**b**) training and (**c**) test set of field-based 3D-QSAR. [the points on the black line indicate that the predicted DPPH values is equal to the experimental DPPH values].

The aligned dataset was randomly distributed to a training set consisting of 23 samples and a test set comprising five samples (Figure S33). A 3D-QSAR model was developed utilizing a field-based approach to establish a correlation between the antioxidative activity of aligned flavonoid dimers and the interaction energy field contributions at each grid point. Partial least squares (PLS) regression analysis with four PLS factors was employed to build the model generated using an extended Gaussian-based potential incorporating steric, electrostatic, hydrophobic, hydrogen bond acceptor, and hydrogen bond donor fields. Each model produced predicted activity values and the most statistically robust model (PLS factor = 4) was identified. In general, a QSAR model is considered valid when it achieves an R^2^ value exceeding 0.6 [28]. As shown in Figure 6a, the optimal model demonstrated a good predictive capability, with an R^2^ (CV) value of 0.842, derived from leave-one-out (LOO) cross-validation. The stability value closely aligned with R^2^ (0.936), and this indicates that there was no overfitting of the model. Furthermore, a high variance ratio (F) and a low probability of error (P) signifies strong predictability for the selected model. External validation metrics which include RMSE (0.09), Q^2^ (0.869), and a Pearson correlation coefficient of 0.975 demonstrate a strong positive linear correlation. The scatterplots shown in Figure 6b illustrate the relationship between the experimental and predicted DPPH values (expressed as mM of Trolox/mM of sample). These scatterplots indicate that the molecules from both datasets lie close to the linear regression line, thereby confirming the reliability of the model at PLS4.

**Figure 7:**
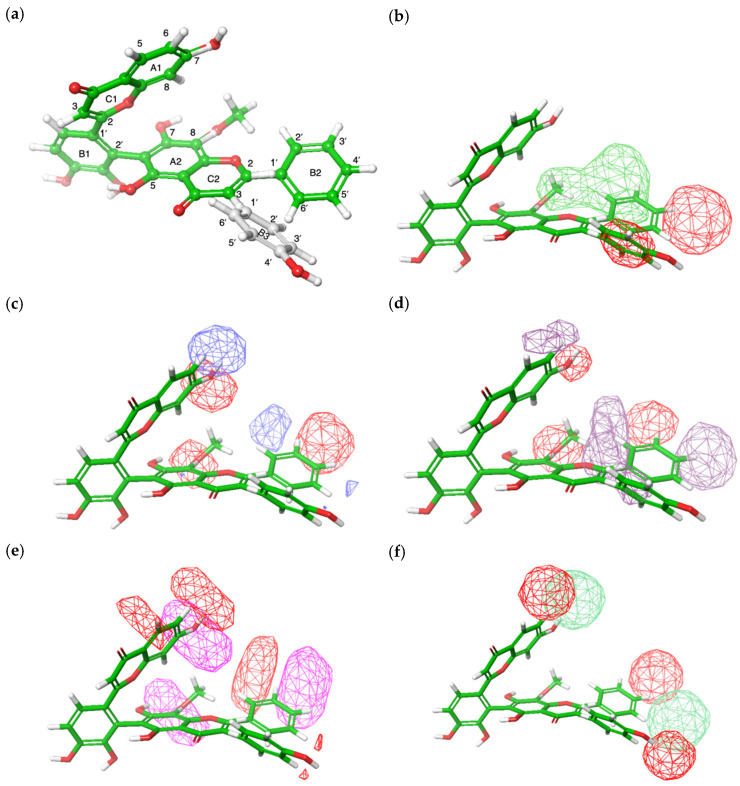
Field-based 3D-QSAR contour maps based on biflavone **24** (highest DPPH value) and biflavone **13** (lowest DPPH value): (**a**) structure of biflavone **24** and **13**; (**b**) steric field (lime green contour represents areas where steric interaction is favored and red represents disfavored); (**c**) electrostatic field (light blue represents favored and red represents disfavored electrostatic interaction); (**d**) hydrophobic field (blue-violet represents favored while red represents disfavored hydrophobic interaction); (**e**) H bond acceptor (magenta and red represent favorable and unfavorable hydrogen bond acceptor interaction, respectively); and (**f**) H bond donor field (light green and red contours represent favorable and unfavorable hydrogen bond donor interactions, respectively).

The contribution values for steric, electrostatic, hydrophobic, hydrogen bond acceptor, and hydrogen bond donor interactions were 0.375, 0.083, 0.153, 0.213, and 0.177, respectively. The molecular field analysis indicated that steric interactions contributed more significantly than other factors. In the steric contour map of the selected compounds (Figure 7b), favorable steric regions were highlighted in lime green, while unfavorable areas were shown in red. Figure 7a provides the flavonoid numbering system to facilitate interpretation of the contour maps, where rings A1, B1, and C1 correspond to the radical precursor, and rings A2, B2/B3, and C2 correspond to the nucleophile precursor. Flavones such as diosmetin and chrysin contain ring B2 while isoflavones such as genistein contain B3. The results showed that the interaction between ring B3 and C2 exhibited an unfavorable steric effect compared to that of ring B2 and C2. Additionally, introducing steric hindrance (e.g., hydroxyl or methoxy groups) on ring B2 at C4′ and C5′ could reduce DPPH radical scavenging activity, whereas increased steric hindrance at C8 of ring A2 and C2′ and C3′ of ring B2 appeared to enhance DPPH activity.

The electrostatic contour map (Figure 7c) showed positive electrostatic potential in light blue regions and negative potential in red regions. Favorable electrostatic interactions were observed for hydroxyl or methoxy groups at C8 of ring A2, C7 of ring A1, and C3′/4′ of ring B2. Conversely, removing these groups from C6 of ring A1 and C2′ of ring B2 could enhance DPPH activity.

Figure 7d shows the hydrophobic contour map of the selected compounds, where hydrophobic-favorable regions were depicted in blue-violet and unfavorable regions in red. Therefore, increasing the presence of hydrophilic groups at C7 of ring A1, C3′ of ring B2, and C8 of ring A2 might lead to enhanced DPPH activity, while increasing hydrophobic groups at C2′ and C4′ of ring B2 could also improve DPPH activity.

In the hydrogen bond acceptor contour map (Figure 7e), the magenta regions indicated that the presence of hydrogen bond acceptors, such as hydroxyl or methoxy groups, at C7 of ring A1, C8 of ring A2, and C3′ of ring B2, would positively influence DPPH activity. In contrast, red regions indicated that the absence of these groups at C5 and C6 of ring A1 and C2′ of ring B2 would enhance activity.

Finally, the light green region in the hydrogen bond donor contour map (Figure 7f) showed that the presence of hydrogen bond donors (hydroxyl groups) at C7 of ring A1 and C4′ of ring B2 would increase DPPH activity. Additionally, the red regions indicated that the absence of hydrogen bond donors at C4′ of ring B2, C4′ of ring B3, and C6 of ring A1 would further increase DPPH activity.

Since steric effects contributed the most to the activity, optimal DPPH activity can be achieved by increasing steric bulk at C8 of ring A2 and C2′/3′ of ring B2.

### 3.6. Density Functional Theory Calculation of Molecular Orbital Energies

**Figure 8:**
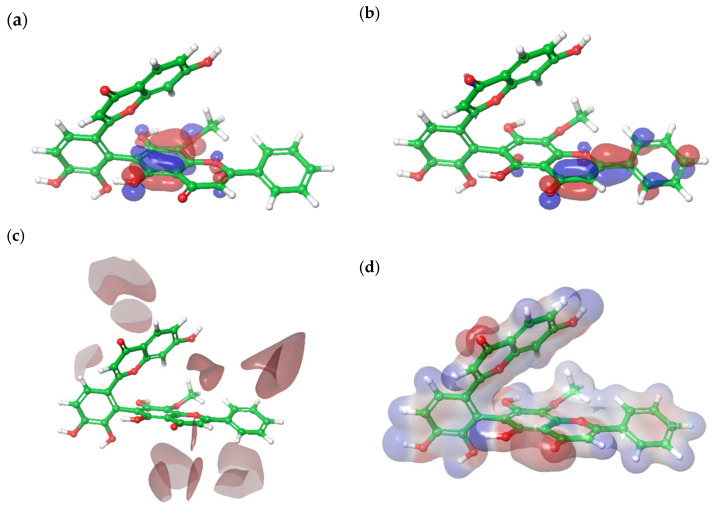

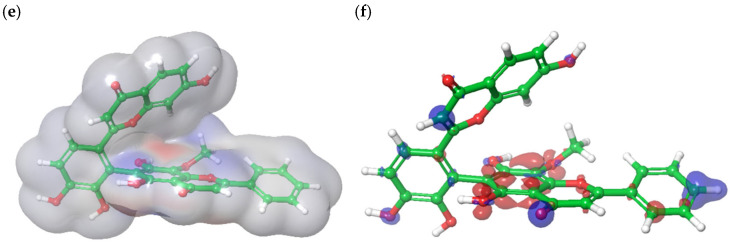
Molecular orbitals of biflavone **24** with blue representing a positive phase while red indicates a negative phase: (**a**) highest occupied molecular orbital (HOMO) and (**b**) lowest unoccupied molecular orbital (LUMO); (**c**) average localization ionization energy (ALIE) mapped on molecular surface of biflavone **24**, where red denotes low ionization energy, white denotes intermediate, and blue represents high ionization energy; (**d**) electrostatic potential (ESP) mapped on molecular surface of biflavone **24**, where red signifies electron rich regions and blue indicates electron-poor region; (**e**) electron density (ED) mapped on molecular surface of biflavone **24**, whereby red signifies less electron density, white is intermediate density, and blue indicates high electron density; (**f**) Fukui indices (F-): red signifies positive and blue negative values.

The HOMO energy is an important parameter to describe the antioxidant ability of a molecule because it is related to electron transfer reactions [29]. The HOMO is mainly localized on ring A2 while LUMO is centered on ring C2 (Figure 8a,b). The HOMO and LUMO energy levels were determined to be −0.222 eV and −0.071 eV, respectively, with an energy gap (ΔE) of 0.151 eV. The small energy gap indicated that biflavone 24 could transfer an electron with less energy input, thereby increasing its likelihood to engage in redox reactions and to donate electrons to neutralize free radicals. Therefore, this indicates that biflavone 24 could possess strong antioxidant potential.

The average localization ionization energy (ALIE) ranged from 198.98 kcal/mol (minimum) to 388.88 kcal/mol (maximum). The lower-ALIE regions indicated areas where the molecule could lose electrons easily, hence facilitating the neutralization of reactive oxygen species. In the ALIE map, the bright red regions around ring B2 and C2 highlighted areas with lower ionization energy (Figure 8c). Additionally, the electrostatic potential (ESP) mapping of biflavone **24** showed values ranging from −60.92 kcal/mol (minimum) to 64.79 kcal/mol (maximum) (Figure 8d). The red regions (carbonyl and hydroxyl group in ring A2, C1, and C2) indicated electron-rich areas which were essential for antioxidant activity. On the other hand, the blue regions signified electron-deficient zones. Apart from ALIE and ESP, the electron density (ED) map emphasized biflavone **24**′s electron-donating potential (Figure 8e). In the ED map, blue regions around C5 and C8 in ring A2 denoted areas of higher electron density.

The Fukui indices highlighted regions of biflavone **24** that were susceptible to both nucleophilic and electrophilic attack. This indicated their potential to either donate or accept electrons depending on the surrounding environment and interacting species. In particular, the F- index identified electron-rich areas with a strong ability for electron donation. These electron-rich areas, highlighted in red (OH groups in ring A2) were potential sites where biflavone **24** could donate electrons to neutralize free radicals (Figure 8f). This finding supported the localization of the HOMO on ring A2, suggesting that this region was likely the primary site for electron donation.

The combination of these electronic properties indicated that biflavone **24** was a promising antioxidant that was capable of donating electrons to neutralize reactive oxygen species (low ALIE, red ESP, F- indices), potentially making it effective in reducing oxidative stress in biological systems.

## 4. Conclusions

In this study, a library of 28 rare biflavonoids with C2′–C6″ interflavonyl linkages was synthesized, isolated, and structurally characterized. This work expands the structural diversity of biflavonoids beyond the predominantly studied C3′–C8″ linked derivatives such as amentoflavone. The synthesis was achieved under green, catalyst-free, and food-grade conditions, enabling direct coupling of flavone monomers without the need for pre-functionalization. Purification and structural confirmation were conducted using semi-preparative HPLC, NMR, and HRMS, ensuring high compound purity and precise characterization. This foundational work establishes a robust platform for future research on biflavonoid bioactivities, structure–activity relationships, and potential nutraceutical and pharmaceutical applications.

In addition, the antioxidant activity of these biflavonoids was evaluated, identifying biflavone **24** as the most potent compound based on DPPH assays. A highly predictive 3D-QSAR model revealed that steric interactions play a dominant role in determining antioxidant potential, while complementary DFT calculations supported the strong antioxidant effect of biflavone **24** by highlighting its low HOMO–LUMO gap, favorable ESP mapping, and electron-donating regions. These findings offer valuable insights into the structure–activity relationships of biflavonoids, guiding the rational design of more effective antioxidants for therapeutic use. However, it is important to acknowledge that the DPPH and QSAR assessments were performed in chemical and computational systems, which may not fully replicate biological conditions. Future studies will incorporate cellular-based assays, such as ROS measurements, to confirm the biological relevance of these biflavonoids and further explore their therapeutic potential.

## Data Availability

The original contributions presented in this study are included in the article/supplementary material. Further inquiries can be directed to the corresponding author(s).

## Supplementary Materials

The following supporting information can be downloaded at https://www.mdpi.com/article/10.3390/antiox14060742/s1: The reaction scheme and HPLC profile of 3′,4′-dihydroxyflavone and wogonin (Figure S1). The ^1^H,^13^C NMR and mean mass of biflavonoids **5a** (Figures S2–S30). HSQC and HMBC of biflavonoids **5** and **5a** (Figures S31–S32). The ^1^H chemical shifts of biflavonoids **1**–**28** (Table S1). The ^13^C chemical shifts of biflavonoids **1**–**28** (Table S2). Predicted DPPH activity from field-based QSAR (Table S3). Alignment of the pharmacophoric features of flavonoid dimers before alignment and after alignment (Figure S33).

## Abbreviations and Nomenclature

ROS: reactive oxygen species, RNS: reactive nitrogen species, QSAR: quantitative structure–activity relationship, LCQ: liquid chromatography quadruple, HPLC: high-pressure liquid chromatography, KOH: potassium hydroxide, PLS: partial least squares, SD: standard deviation, B3LYP: Becke’s three-parameter exchange potential and Lee–Yang–Parr correlation, HOMO: highest occupied molecular orbital. LUMO: lowest unoccupied molecular orbital, ESP: electrostatic potential, ALIE: average local ionization energy, ED: electron density.
