# Supplementary material for: Food Grade Synthesis of Hetero-Coupled Biflavones and 3D-Quantitative Structure–Activity Relationship (QSAR) Modeling of Antioxidant Activity
Source: Antioxidants (Basel). 2025 Jun 16;14(6):742. doi: 10.3390/antiox14060742 (PMC12189461; doi:10.3390/antiox14060742)
Supplement: Supplementary file 1 [file antioxidants-14-00742-s001.zip › antioxidants-3668507-supplementary.pdf]

SUPPORTING INFORMATION

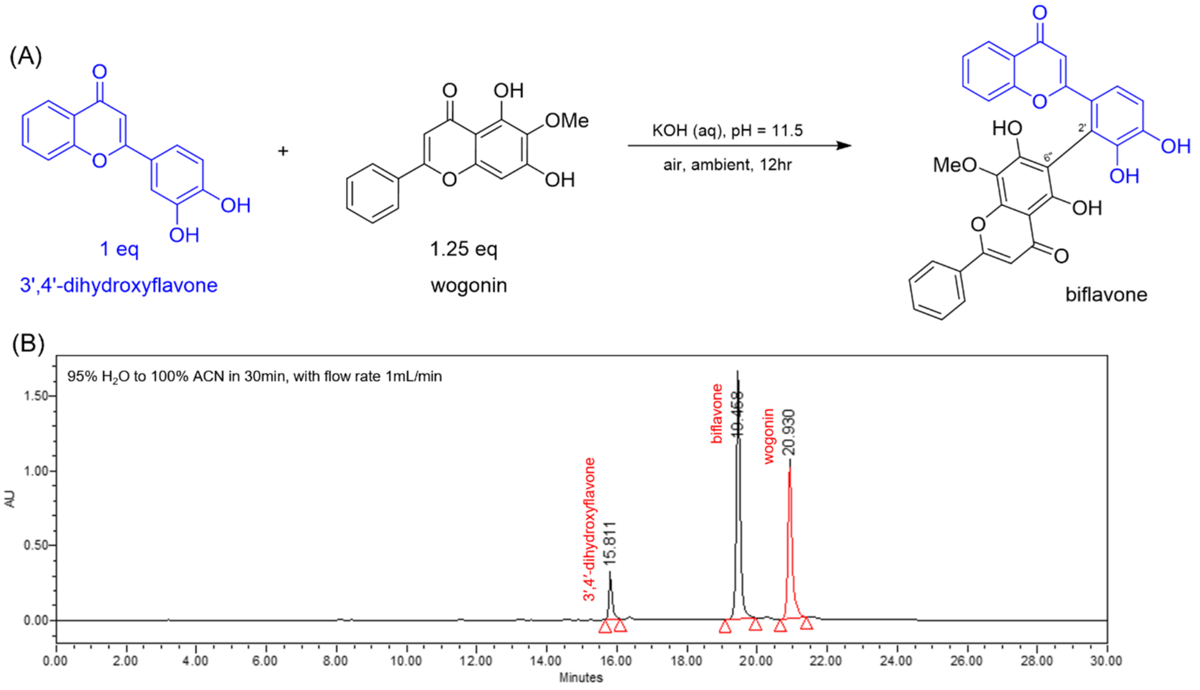

Figure S1. **A**) Reaction scheme between 3',4''-dihydroxyflavone and wogonin. **B**) HPLC chromatogram of the reaction mixtures

^{1}H and ^{13}C NMR and biflavones 1-28

1 Diosmetin + 3',4'-dihydroxyflavone (6-(2,3-dihydroxy-6-(4-oxo-4H-chromen-2-yl)phenyl)-5,7-dihydroxy-2-(3-hydroxy-4-methoxyphenyl)-4H-chromen-4-one), 9 mg, 7 % yield, yellow solid. Semi-prep HPLC, injection volume was 250 μL. ACN/Water = 40: 60, flow rate 5.0 mL/min, λ = 300 nm. ^{1}H NMR (500 MHz, DMSO-*d6*) δ 13.09 (s, 1H), 9.49 (s, 1H), 7.91 (d, *J* = 7.4 Hz), 7.69 (dd, *J* = 8.0 Hz, 1H), 7.55 (d, *J* = 8.6 Hz, 1H), 7.44 (s, 1H), 7.38 (dd, *J* = 8.0 Hz, 1H), 7.27 (d, *J* = 8.6 Hz, 1H, H-8), 7.22 (d, *J* = 8.5 Hz, 1H, H-6'), 7.08 (d, *J* = 8.6 Hz, 1H, H-5'''), 6.96 (d, *J* = 8.8 Hz, 1H), 6.76 (s, 1H), 6.55 (s, 1H), 6.12 (s, 1H), 3.86 (s, 3H). ^{13}C NMR (126 MHz, DMSO) δ 182.2, 177.0, 166.2, 163.8, 162.6, 159.3, 156.8, 156.2, 151.6, 148.6, 147.3, 144.8, 134.5, 125.7, 125.2, 124.8, 123.5, 123.4, 120.6, 120.2, 119.2, 118.3, 114.8, 113.4, 112.7, 109.0, 108.6, 104.0, 103.9, 93.7, 56.2. HRMS(ESI-TOF) **[M-H]**$^-$ calculated for $C_{31}H_{19}O_{10}$ = 551.0984, found 551.0981.

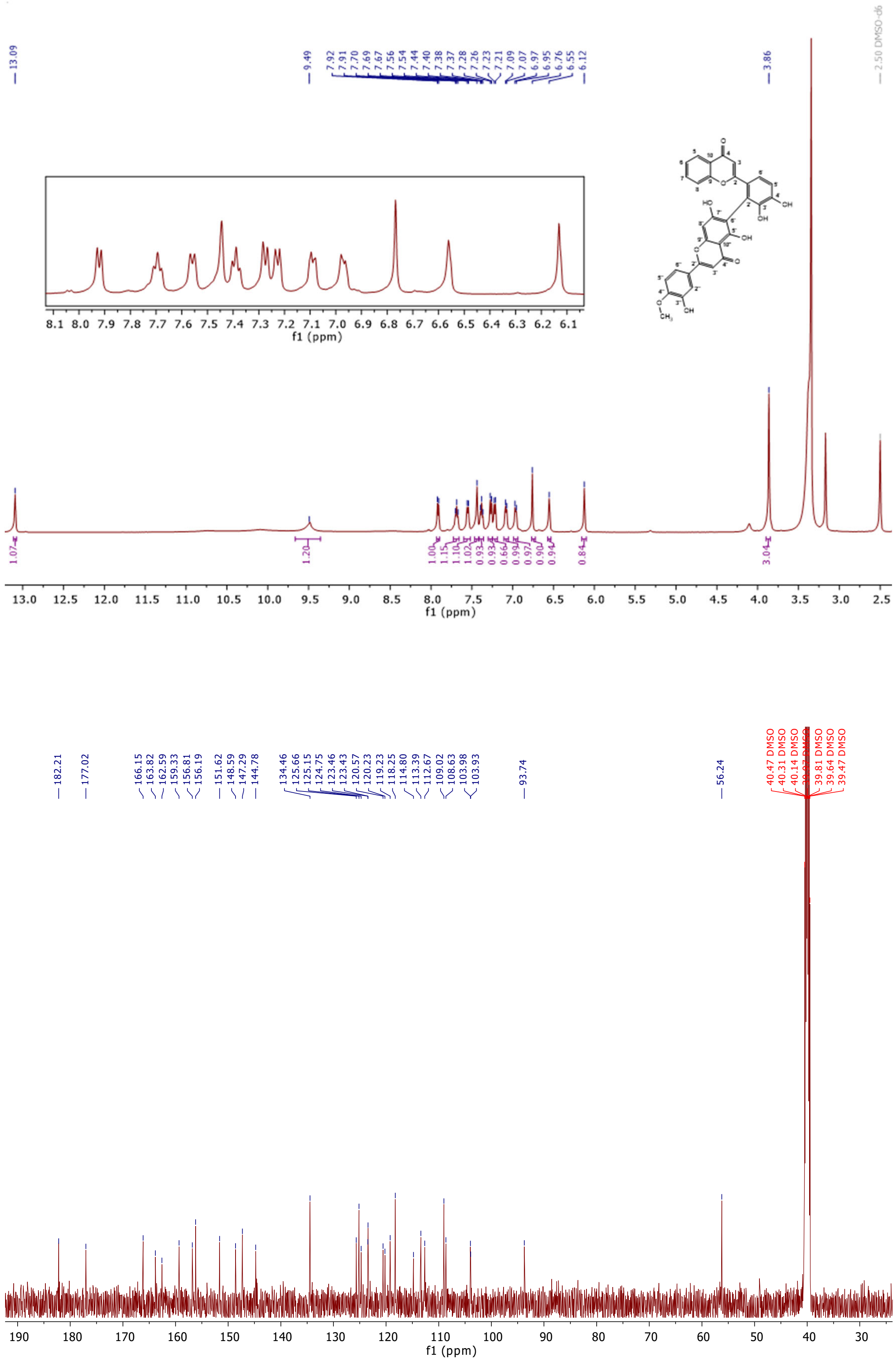

Figure S2.1. ^{1}H and ^{13}C NMR of biflavone 1

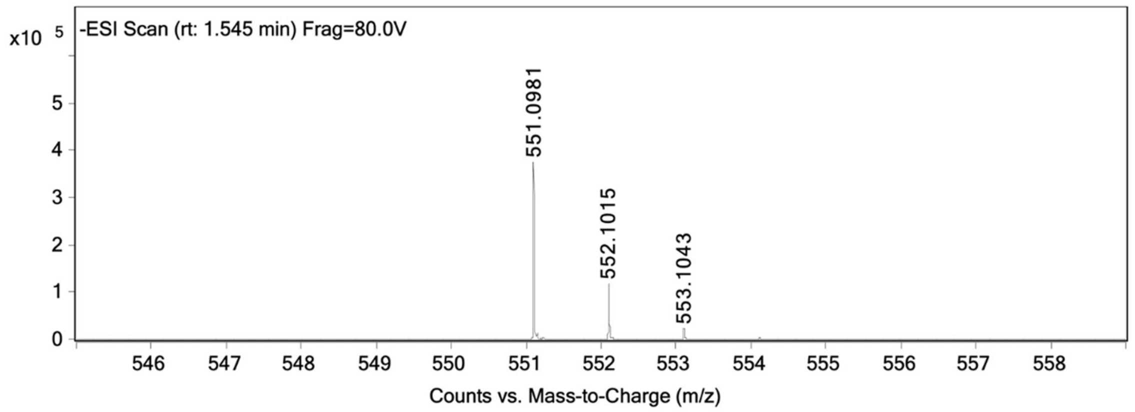

Figure S2.2. HRMS spectrum of biflavone 1

2 Diosmetin + 5,3',4'-trihydroxyflavone (6-(2,3-dihydroxy-6-(5-hydroxy-4-oxo-4H-chromen-2-yl)phenyl)-5,7-dihydroxy-2-(3-hydroxy-4-methoxyphenyl)-4H-chromen-4-one), 7.76 mg, 6 % yield, yellow solid. Semi-prep HPLC, injection volume was 250 µL. ACN/Water = 45: 55, flow rate 5.0 mL/min, λ = 300 nm, ^{1}H NMR (500 MHz, DMSO-*d6*) δ 13.11 (s, 1H), 12.64 (s, 1H), 9.50 (s, 1H), 7.59 – 7.50 (m, 2H), 7.44 (s, 1H), 7.24 (d, *J* = 8.4 Hz, 1H), 7.09 (d, *J* = 8.6 Hz, 1H), 6.96 (d, *J* = 8.5 Hz, 1H), 6.76 (s, 1H), 6.70 (d, *J* = 8.3 Hz, 1H), 6.65 (d, *J* = 8.5 Hz, 1H), 6.55 (s, 1H), 6.18 (s, 1H), 3.87 (s, 3H). ^{13}C NMR (126 MHz, DMSO) δ 182.9, 182.2, 168.0, 163.8, 160.3, 159.3, 156.9, 156.4, 151.6, 149.2, 147.3, 144.9, 136.2, 124.0, 123.5, 121.0, 120.4, 119.2, 114.8, 113.4, 112.7, 111.1, 110.1, 108.5, 107.3, 107.2, 104.0, 103.8, 93.8, 56.2. HRMS(ESI-TOF) [**M**-**H**]$^-$ calculated for $C_{31}H_{19}O_{11}$ = 567.0933, found 567.0931.

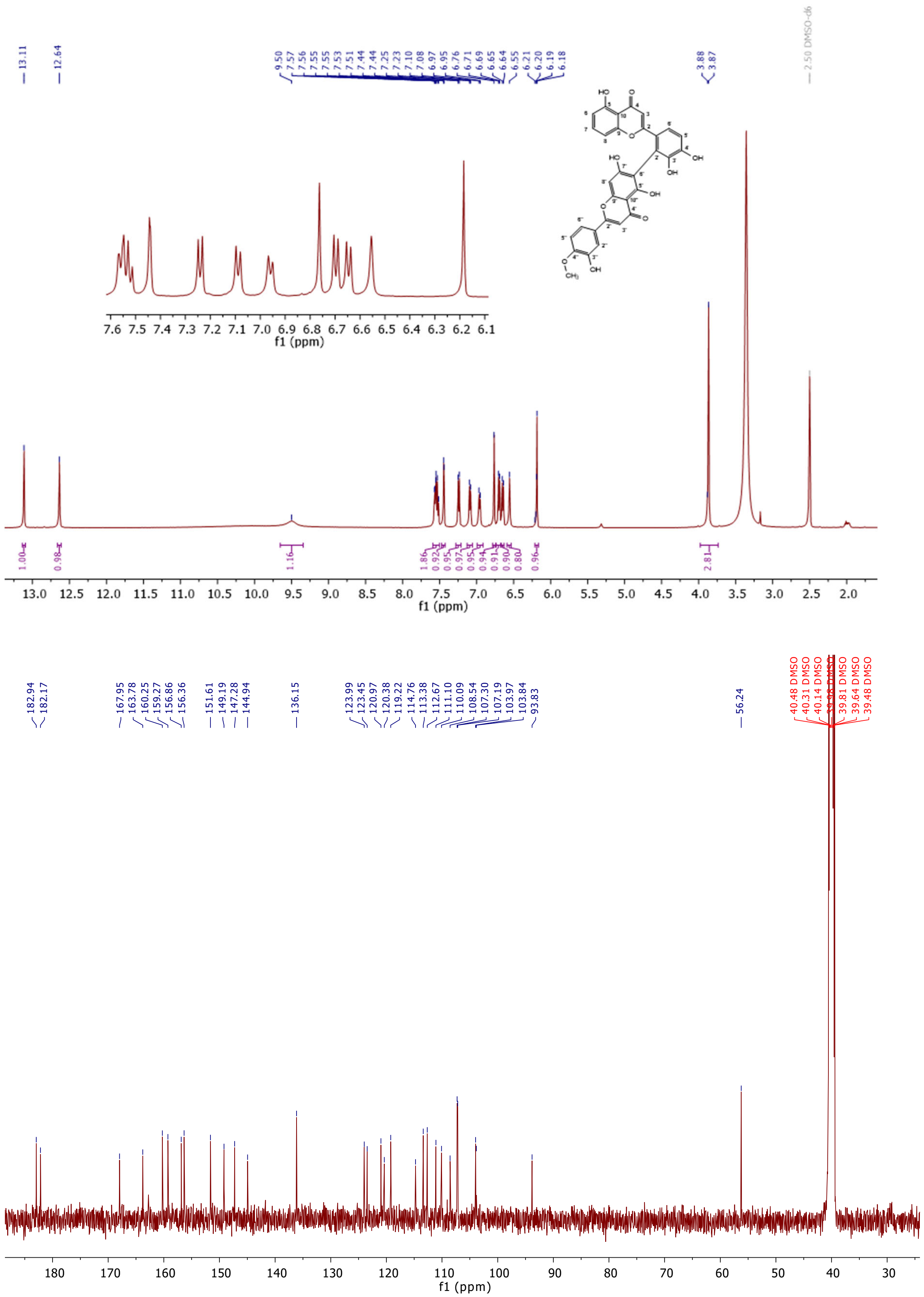

Figure S3.1. ^{1}H and ^{13}C NMR of biflavone 2

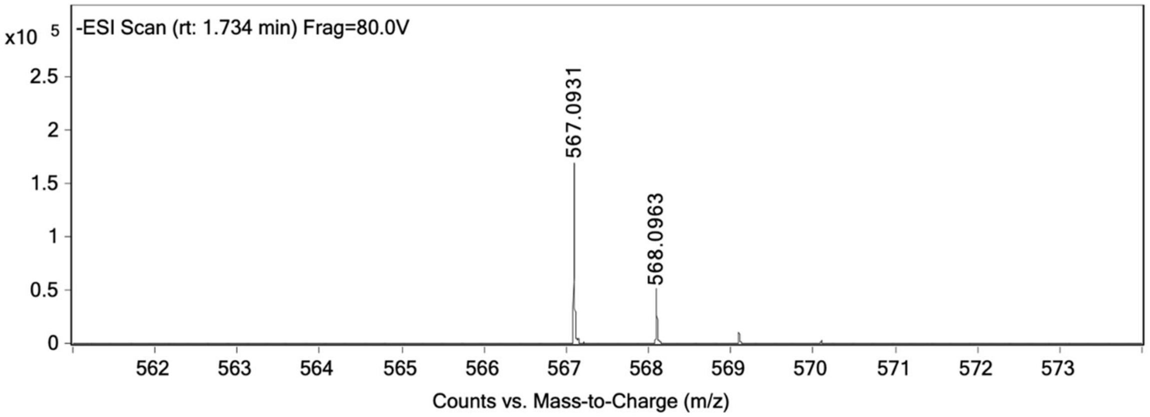

Figure S3.1. HRMS spectrum of biflavone 2

3 Diosmetin + 6,3',4'-trihydroxyflavone (6-(2,3-dihydroxy-6-(6-hydroxy-4-oxo-4H-chromen-2-yl)phenyl)-5,7-dihydroxy-2-(3-hydroxy-4-methoxyphenyl)-4H-chromen-4-one), 13.64 mg, 9% yield, yellow solid. Semi-prep HPLC, injection volume was 250 µL. ACN/Water = 35: 65, flow rate 5.0 mL/min, λ = 300 nm, ^{1}H NMR (500 MHz, DMSO-*d6*) δ 13.07 (d, *J* = 2.3 Hz, 1H), 10.73 (s, 1H), 10.06 (s, 1H), 9.90 (s, 1H), 9.50 (s, 1H), 8.42 (s, 1H), 8.13 (s, 1H), 7.56 (d, *J* = 8.5 Hz, 1H), 7.45 (s, 1H), 7.19 -7.12 (m, 3H), 7.10 (d, J=8.3 Hz, 1H), 6.96 (d, *J* = 8.3 Hz, 1H), 6.76 (s, 1H), 6.55 (s, 1H), 6.04 (s, 1H), 3.87 (s, 3H, H on methoxy substituent). ^{13}C NMR (126 MHz, DMSO) δ 182.2, 176.9, 165.7, 163.8, 162.5, 159.3, 156.8, 155.1, 151.6, 149.9, 148.4, 147.3, 144.7, 125.0, 124.3, 123.5, 123.3, 120.4, 120.1, 119.6, 119.2, 114.8, 113.4, 112.7, 108.7, 108.1, 107.8, 104.0, 103.9, 93.7, 56.2. HRMS(ESI-TOF) [**M-H**]$^-$ calculated for $C_{31}H_{19}O_{11}$ = 567.0933, found 567.0931.

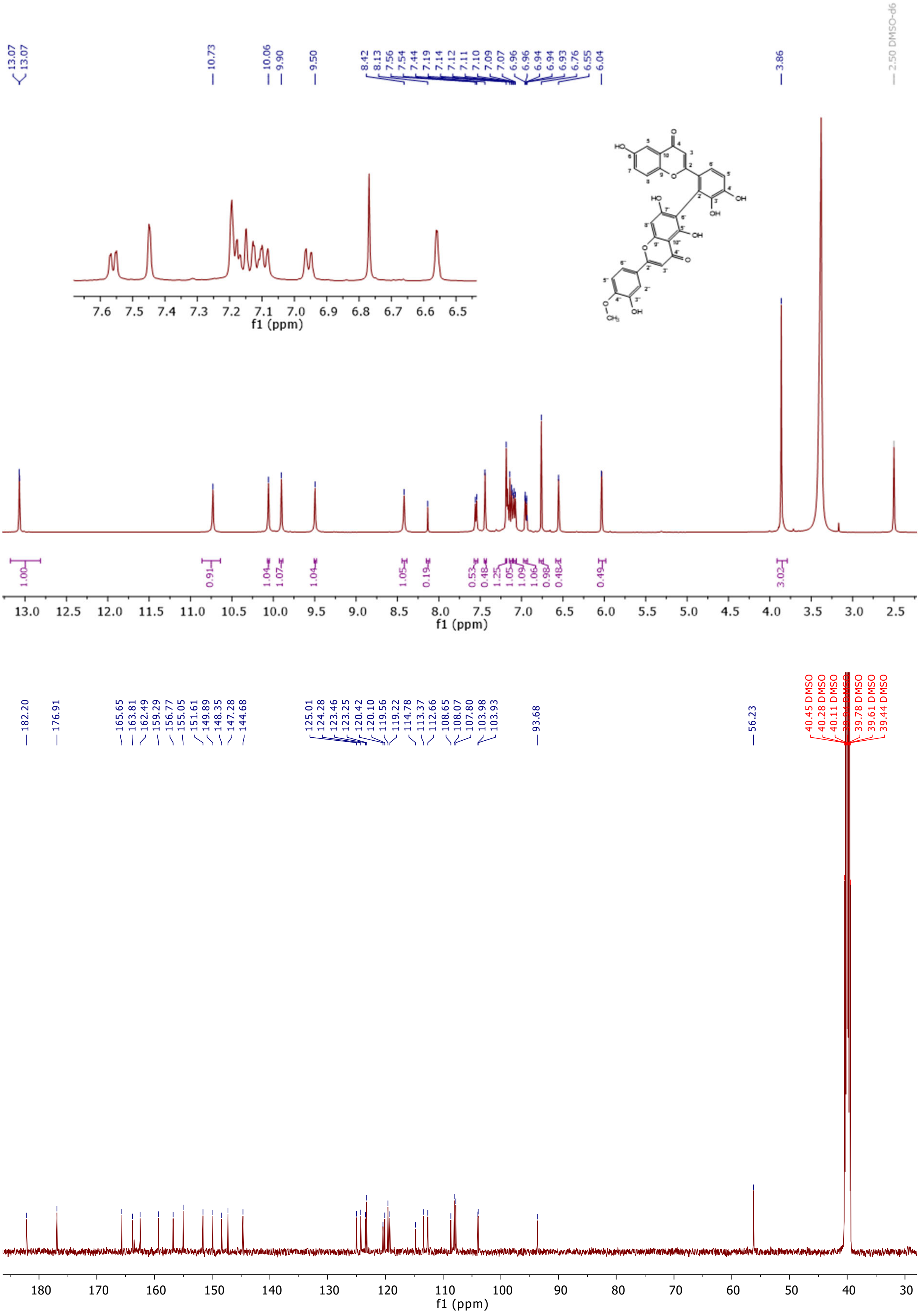

Figure S4.1. ^{1}H and ^{13}C NMR of biflavone 3

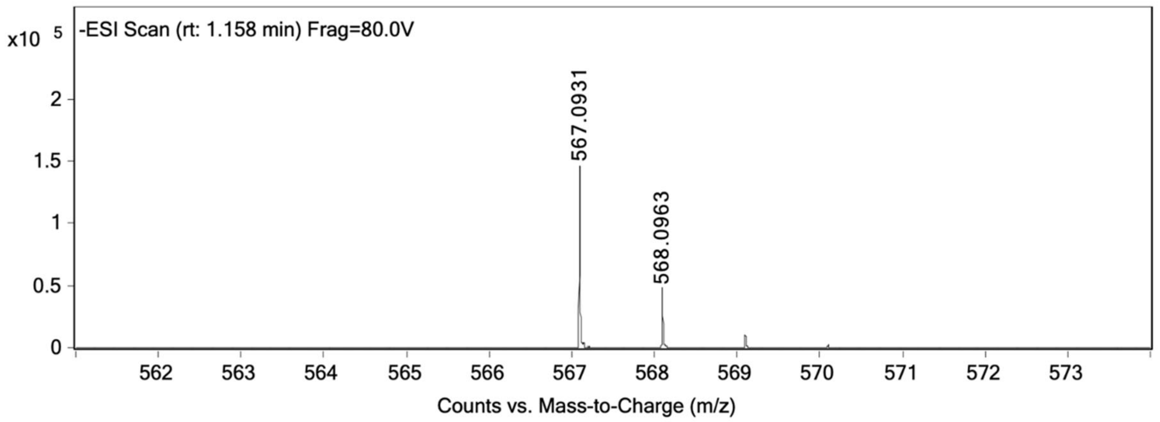

Figure S4.2. HRMS spectrum of biflavone 3

4 Diosmetin + 7,3',4'-trihydroxyflavone (6-(2,3-dihydroxy-6-(7-hydroxy-4-oxo-4H-chromen-2-yl)phenyl)-5,7-dihydroxy-2-(3-hydroxy-4-methoxyphenyl)-4H-chromen-4-one), 14.86 mg, 11% yield, yellow solid. Semi-prep HPLC, injection volume was 250 μL. ACN/Water = 40: 60, flow rate 5.0 mL/min, λ = 300 nm, ^{1}H NMR (500 MHz, DMSO-*d6*) δ 13.09 (s, 1H), 9.52 (s, 1H), 7.75 (d, *J* = 8.7 Hz, 1H), 7.56 (dd, *J* = 8.5, 2.3 Hz, 1H), 7.45 (d, *J* = 2.3 Hz, 1H), 7.17 (d, *J* = 8.4 Hz, 1H), 7.09 (d, *J* = 8.6 Hz, 1H), 6.95 (d, *J* = 8.4 Hz, 1H), 6.81 (dd, *J* = 8.7, 2.3 Hz, 1H), 6.77 (s, 1H), 6.55 (s, 1H), 6.49 (d, *J* = 2.2 Hz, 1H), 6.01 (s, 1H), 3.87 (s, 3H). ^{13}C NMR (126 MHz, DMSO) δ 182.2, 176.5, 165.5, 163.8, 162.8, 162.6, 159.3, 158.0, 156.8, 151.6, 148.3, 147.3, 144.7, 126.8, 124.9, 123.5, 120.4, 120.1, 119.2, 116.2, 115.2, 114.7, 113.37, 112.7, 108.7, 108.6, 104.0, 103.9, 102.4, 93.7, 56.2. HRMS(ESI-TOF) [**M-H**]$^-$ calculated for $C_{31}H_{19}O_{11}$ = 567.0933, found 567.0931.

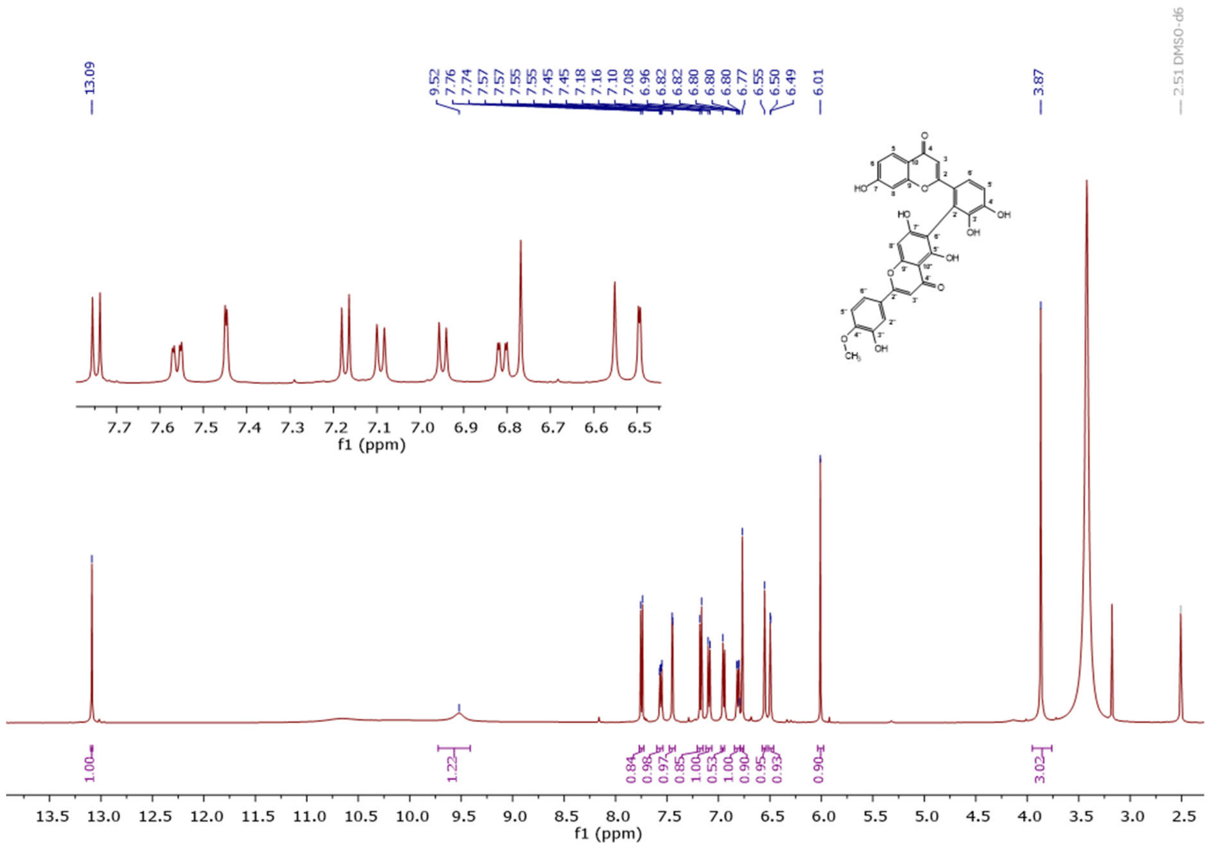

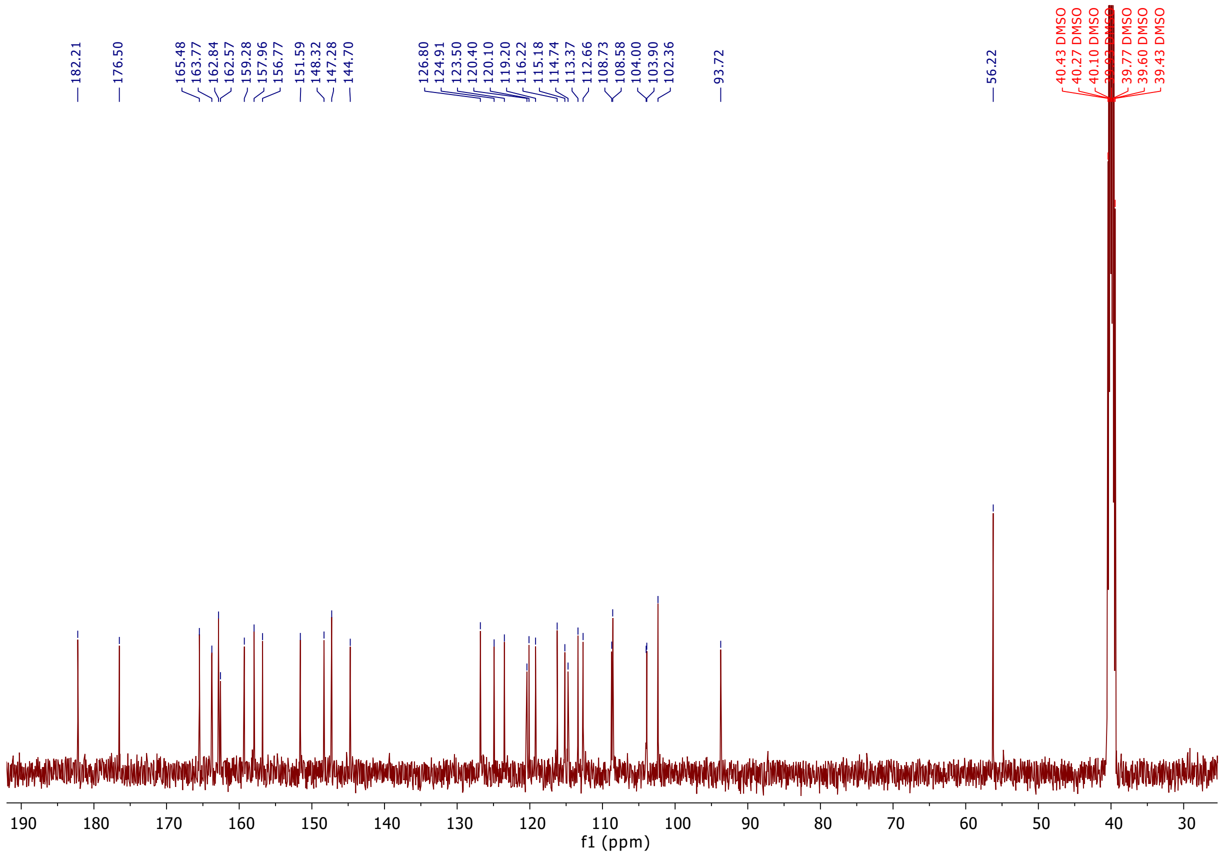

Figure S5.1. ^{1}H and ^{13}C NMR of biflavone 4

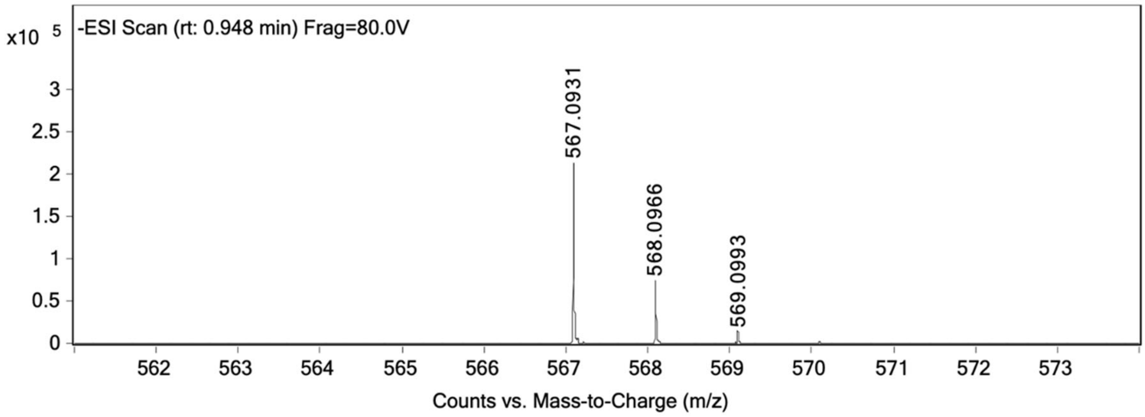

Figure S5.2. ^{1}H and ^{13}C NMR of biflavone 4

5 Chrysin + 3',4'-dihydroxyflavone (6-(2,3-dihydroxy-6-(4-oxo-4H-chromen-2-yl)phenyl)-5,7-dihydroxy-2-phenyl-4H-chromen-4-one), 24 mg, 19 % yield, yellow solid. Semi-prep HPLC, injection volume was 250 µL. ACN/Water = 40: 60, flow rate 5.0 mL/min, λ = 300 nm, ^{1}H NMR (500 MHz, DMSO-*d6*) δ 13.00 (s, 1H), 8.07 (d, *J* = 7.4 Hz, 2H), 7.91 (d, *J* = 7.9 Hz, 1H,), 7.68 (dd, *J* = 7.1 Hz, 1H), 7.63 – 7.52 (m, 3H), 7.37 (dd, *J* = 7.5 Hz), 7.27 (d, *J* = 8.4 Hz, 1H), 7.23 (d, *J* = 8.3 Hz, 1H), 7.00 – 6.95 (m, 2H), 6.62 (s, 1H), 6.14 (s, 1H). ^{13}C NMR (126 MHz, DMSO) δ 182.4, 177.0, 166.1, 163.5, 162.8, 159.3, 157.0, 156.2, 148.6, 144.8, 134.5, 132.5, 131.2, 129.6, 126.9, 125.7, 125.1, 124.8, 123.4, 120.6, 120.1, 118.2, 114.8, 109.0, 108.8, 105.7, 104.2, 93.9. HRMS(ESI-TOF) [**M-H**]$^{-}$ calculated for $C_{30}H_{17}O_8$ = 505.0929, found 505.0926.

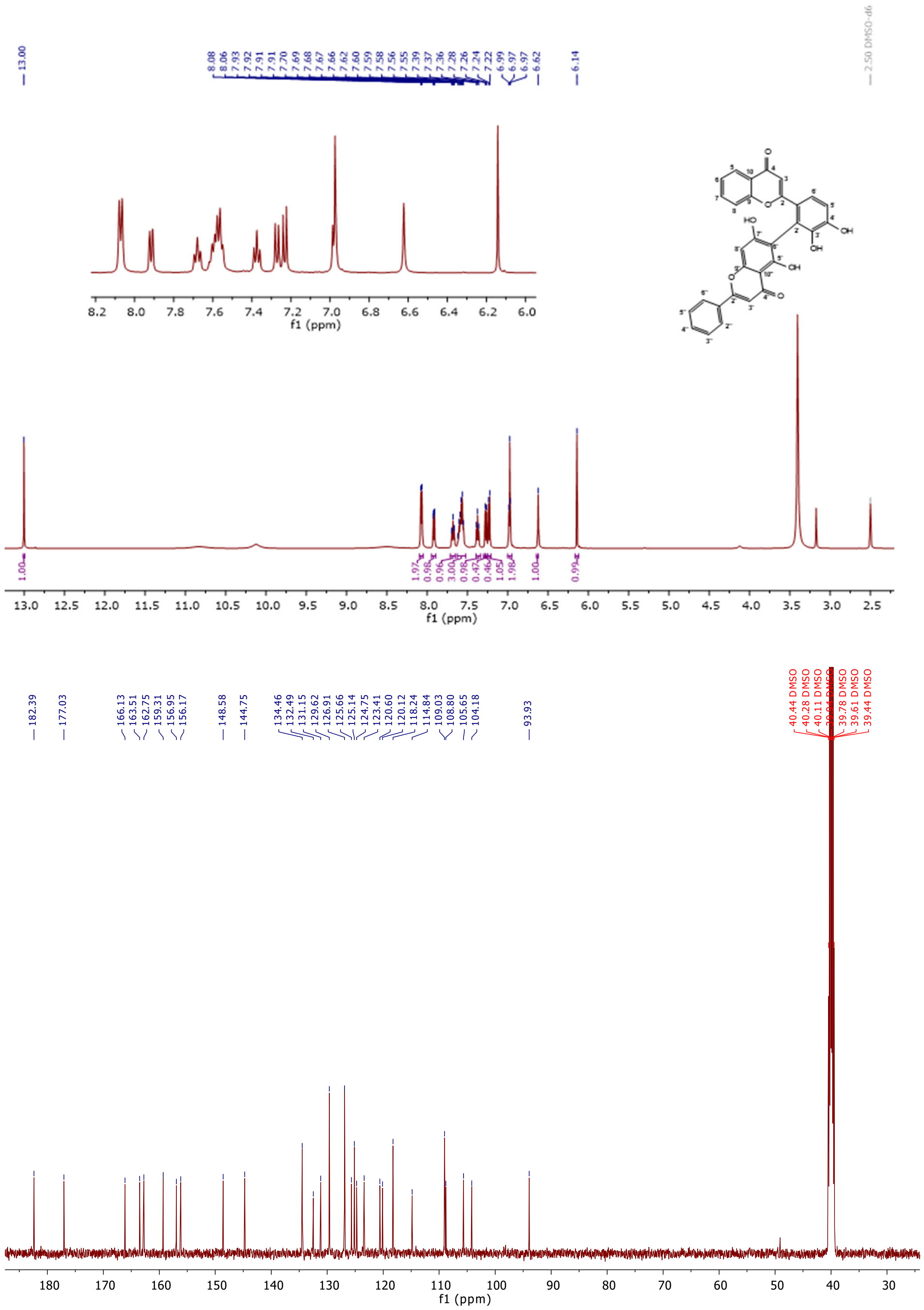

Figure S6.1. ^{1}H and ^{13}C NMR of biflavone 5

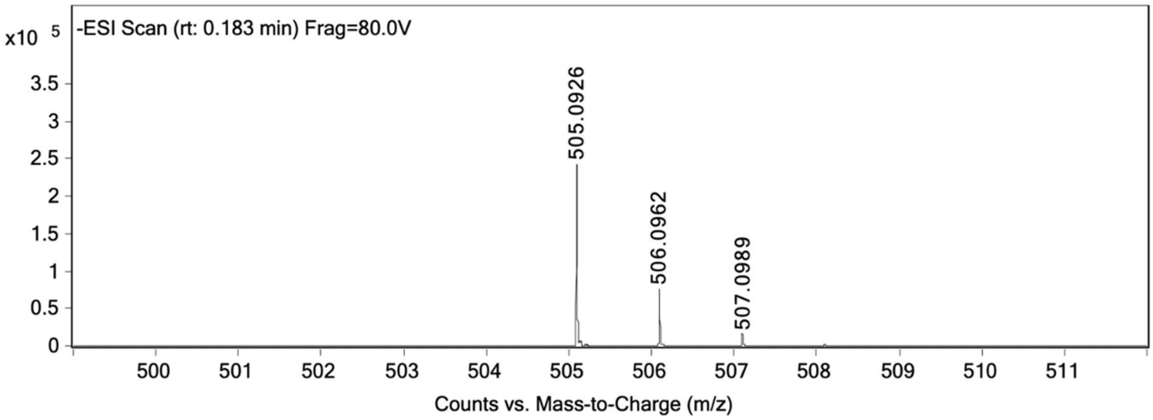

Figure S6.2 HRMS spectrum of biflavone 5

6 Chrysin + 5,3',4'-trihydroxyflavone (6-(2,3-dihydroxy-6-(5-hydroxy-4-oxo-4H-chromen-2-yl)phenyl)-5,7-dihydroxy-2-phenyl-4H-chromen-4-one), 8.97 mg, 7% yield, yellow solid. Semi-prep HPLC, injection volume was 250 μL. ACN/Water = 50: 50, flow rate 5.0 mL/min, λ = 300 nm, ^{1}H NMR (500 MHz, DMSO-*d6*) δ 13.01 (s, 1H), 12.63 (s, 1H), 8.09 (d, *J* = 7.5 Hz, 2H), 7.66 – 7.56 (m, 3H), 7.53 (dd, *J* = 8.3 Hz, 1H), 7.26 (d, *J* = 8.3 Hz, 1H), 6.98 (m, 2H), 6.70 (d, *J* = 8.1 Hz, 1H), 6.68 – 6.62 (m, 2H), 6.20 (s, 1H). ^{13}C NMR (126 MHz, DMSO) δ 183.0, 182.3, 167.9, 163.5, 162.8, 160.3, 159.3, 157.0, 156.4, 149.2, 144.9, 136.2, 132.5, 131.2, 129.6, 126.9, 124.0, 121.0, 120.3, 114.9, 111.1, 110.1, 108.7, 107.4, 107.2, 105.7, 104.2, 94.0. HRMS(ESI-TOF) **[M-H]**$^{-}$ calculated for $C_{30}H_{17}O_{9}$ = 521.0878, found 521.0876.

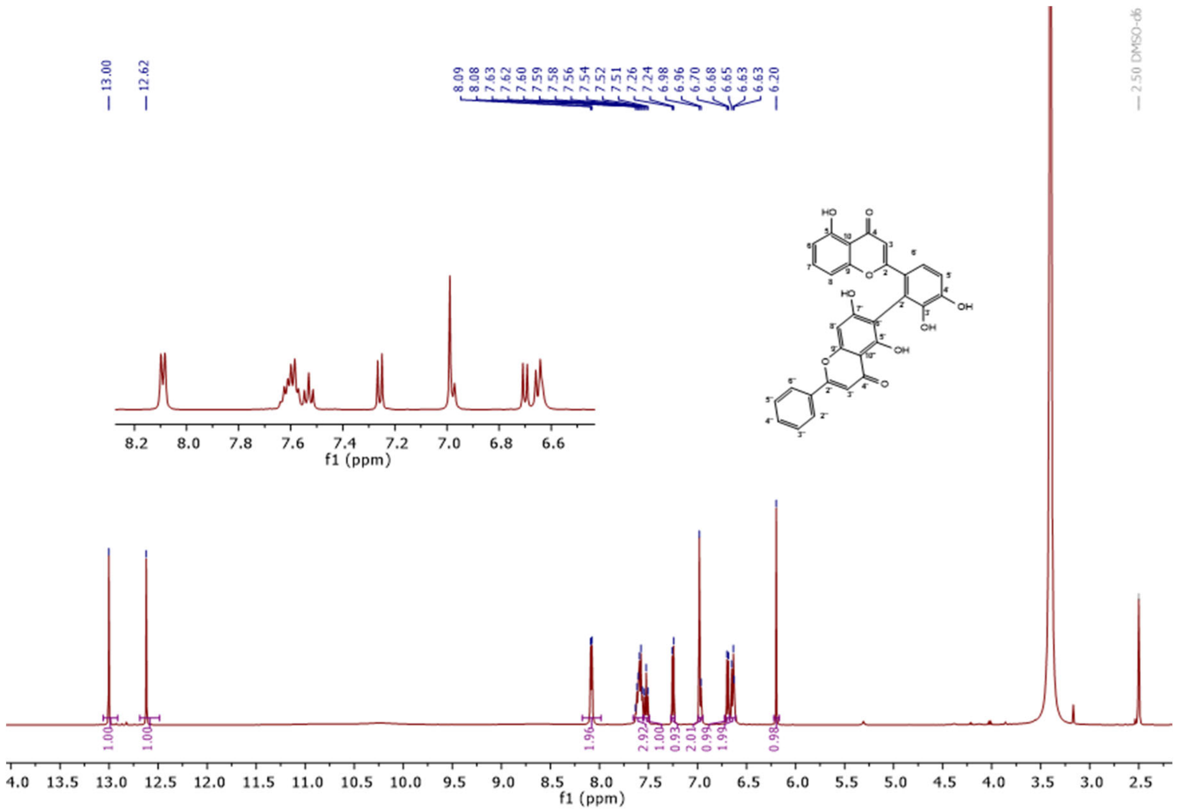

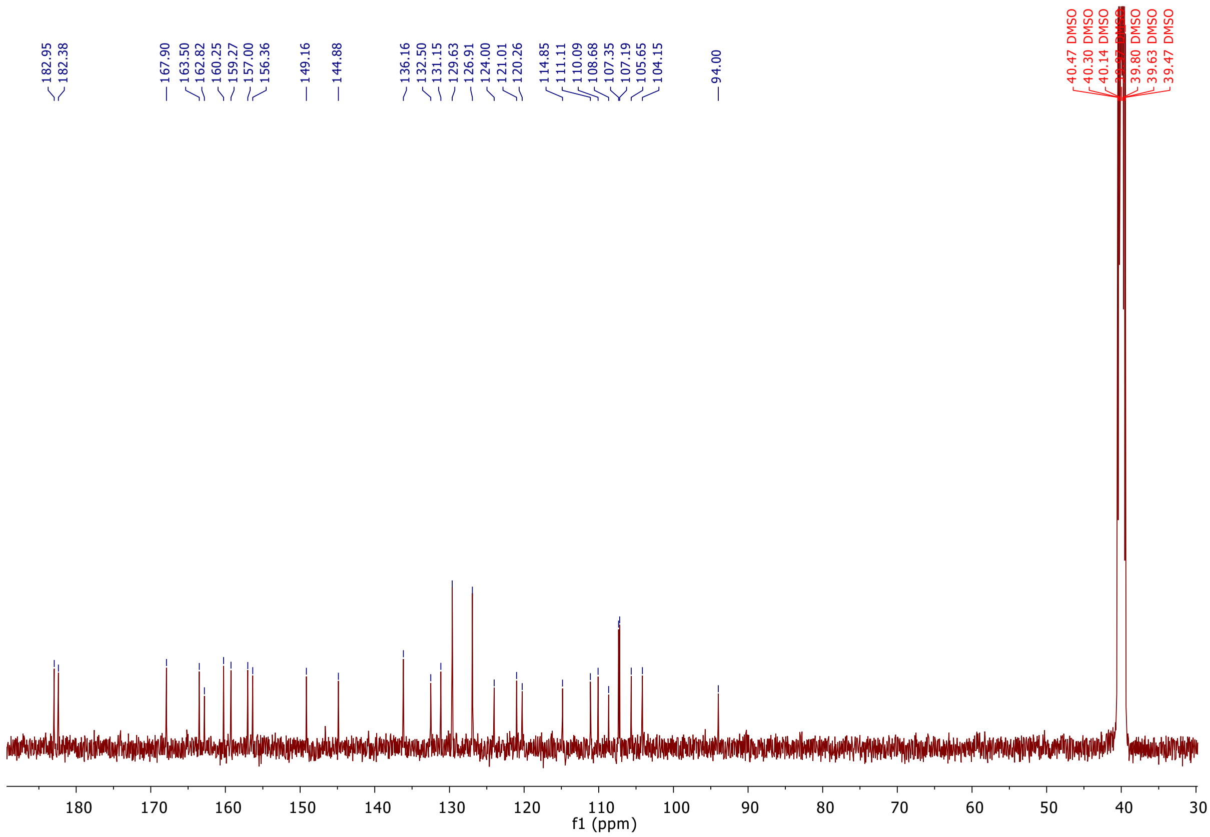

Figure S7.1. ^{1}H and ^{13}C NMR of biflavone 6

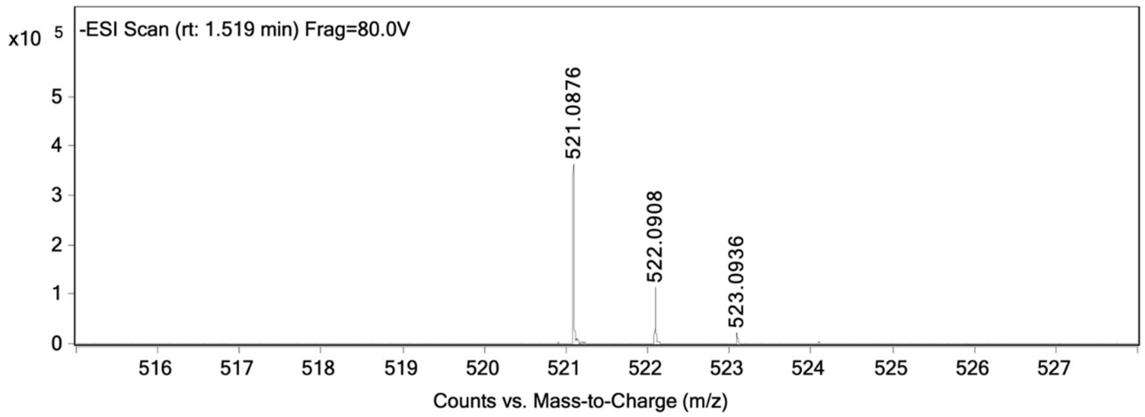

Figure S7.2. HRMS spectrum of biflavone 6

7 Chrysin + 6,3',4'-trihydroxyflavone (6-(2,3-dihydroxy-6-(6-hydroxy-4-oxo-4H-chromen-2-yl)phenyl)-5,7-dihydroxy-2-phenyl-4H-chromen-4-one), 21.62 mg, 17% yield, yellow solid. Semi-prep HPLC, injection volume was 250 μL. ACN/Water = 45: 55, flow rate 5.0 mL/min, λ = 300 nm, ^{1}H NMR (500 MHz, DMSO-*d6*) δ 12.97 (d, *J* = 3.6 Hz, 1H), 10.80 (s, 1H), 10.06 (s, 1H), 9.89 (s, 1H), 8.44 (s, 1H), 8.08 (d, *J* = 7.4 Hz, 2H), 7.66 – 7.52 (m, 3H), 7.20 – 7.17 (m, 2H), 7.15 – 7.09 (m, 2H), 6.98 (s, 1H), 6.95 (d, *J* = 8.5 Hz, 1H), 6.61 (s, 1H), 6.04 (s, 1H). ^{13}C NMR (126 MHz, DMSO) δ 182.4, 176.9, 165.7, 163.5, 162.8, 159.3, 156.9, 155.1, 149.9, 148.4, 144.7, 132.5, 131.2, 129.6, 126.9, 125.0, 124.3, 123.3, 120.4, 120.0, 119.6, 114.8, 108.9, 108.1, 107.8, 105.7, 104.2, 93.9. HRMS(ESI-TOF) [**M**-**H**]$^{-}$ calculated for $C_{30}H_{17}O_9$ = 521.0878, found 521.0877.

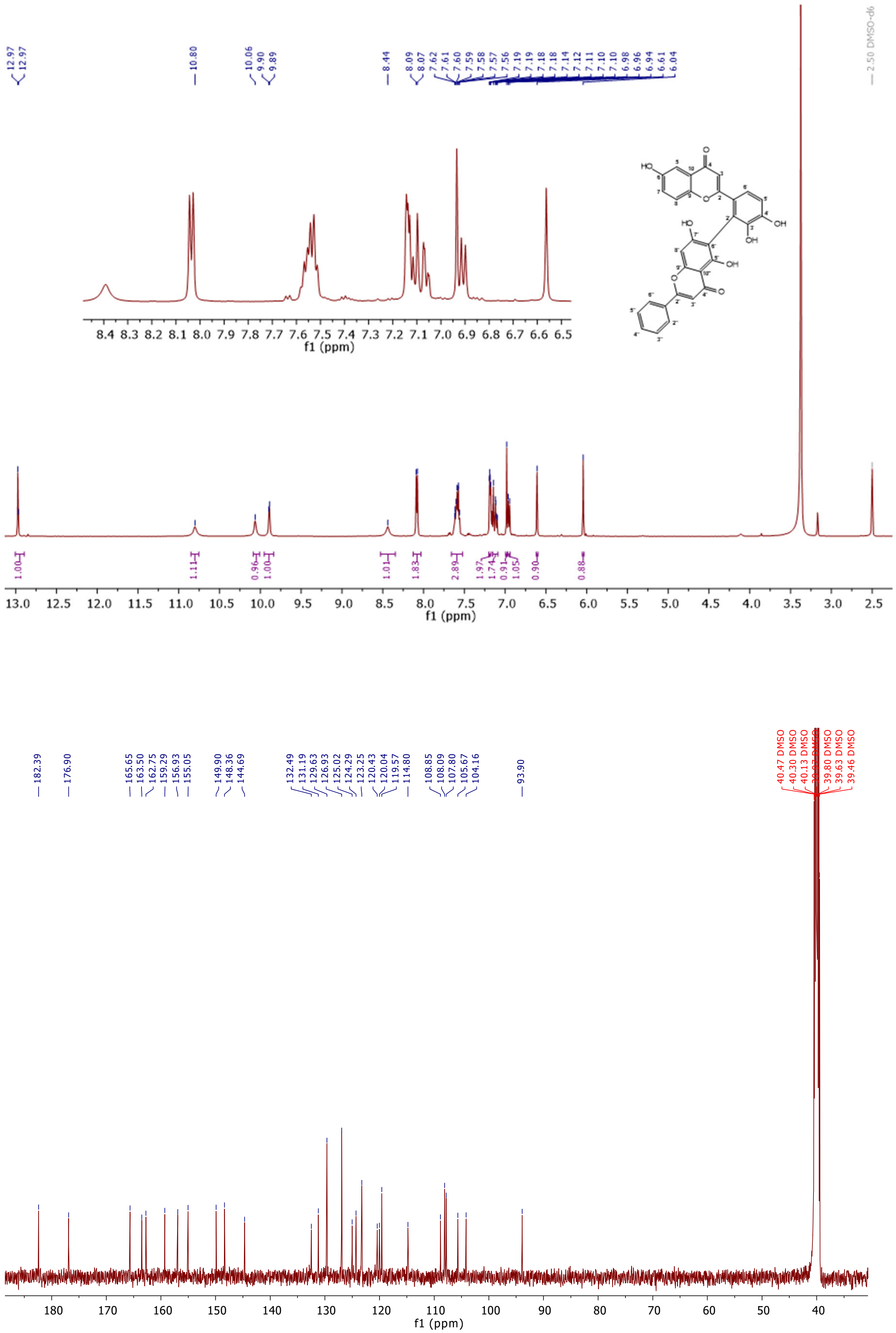

Figure S8.1. ^{1}H and ^{13}C NMR of biflavone 7

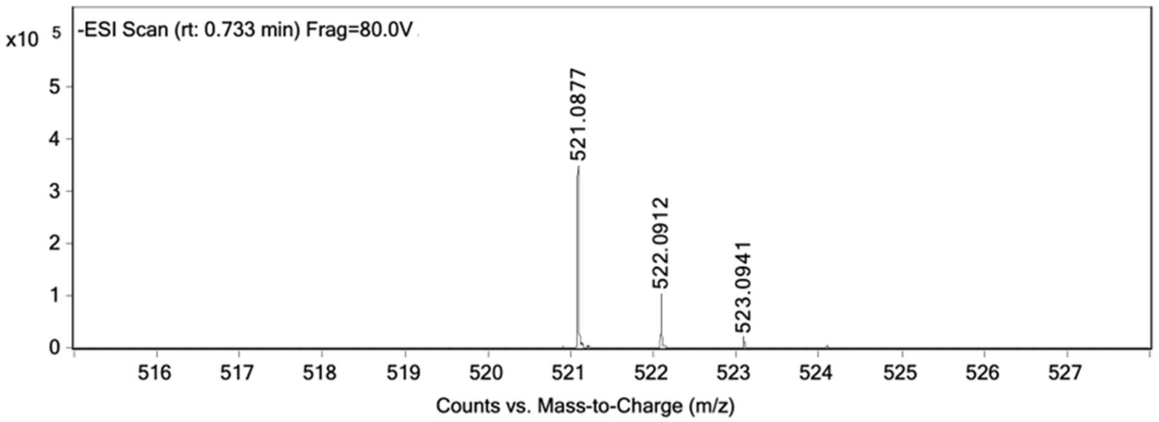

Figure S8.2.HRMS spectrum of biflavone 7

8 Chrysin + 7,3',4'-trihydroxyflavone (6-(2,3-dihydroxy-6-(7-hydroxy-4-oxo-4H-chromen-2-yl)phenyl)-5,7-dihydroxy-2-phenyl-4H-chromen-4-one), 21.51 mg, 17% yield, yellow solid. Semi-prep HPLC, injection volume was 250 μL. ACN/Water = 40: 60, flow rate 5.0 mL/min, λ = 300 nm, ^{1}H NMR (500 MHz, DMSO-*d6*) δ 13.10 – 12.87 (m, 1H), 8.09 (d, *J* = 7.9 Hz, 2H), 7.73 (d, *J* = 8.4 Hz), 7.62 – 7.57 (m, 3H), 7.16 (d, *J* = 8.2 Hz), 6.99 (s, 1H), 6.94 (d, *J* = 8.1 Hz, 1H), 6.80 (d, *J* = 8.1 Hz, 1H), 6.60 (s, 1H), 6.48 (s, 1H), 6.00 (s, 1H). ^{13}C NMR (126 MHz, DMSO) δ 182.4, 176.5, 165.4, 163.5, 162.8, 159.3, 156.9, 148.3, 144.7, 144.7, 143.8, 139.0, 132.5, 132.5, 131.2, 129.6, 126.9, 126.8, 124.9, 120.4, 120.0, 115.2, 114.8, 108.9, 108.6, 105.7, 102.4, 93.9. HRMS(ESI-TOF) [**M-H**]$^{-}$ calculated for $C_{30}H_{17}O_9$ = 521.0878, found 521.0877.

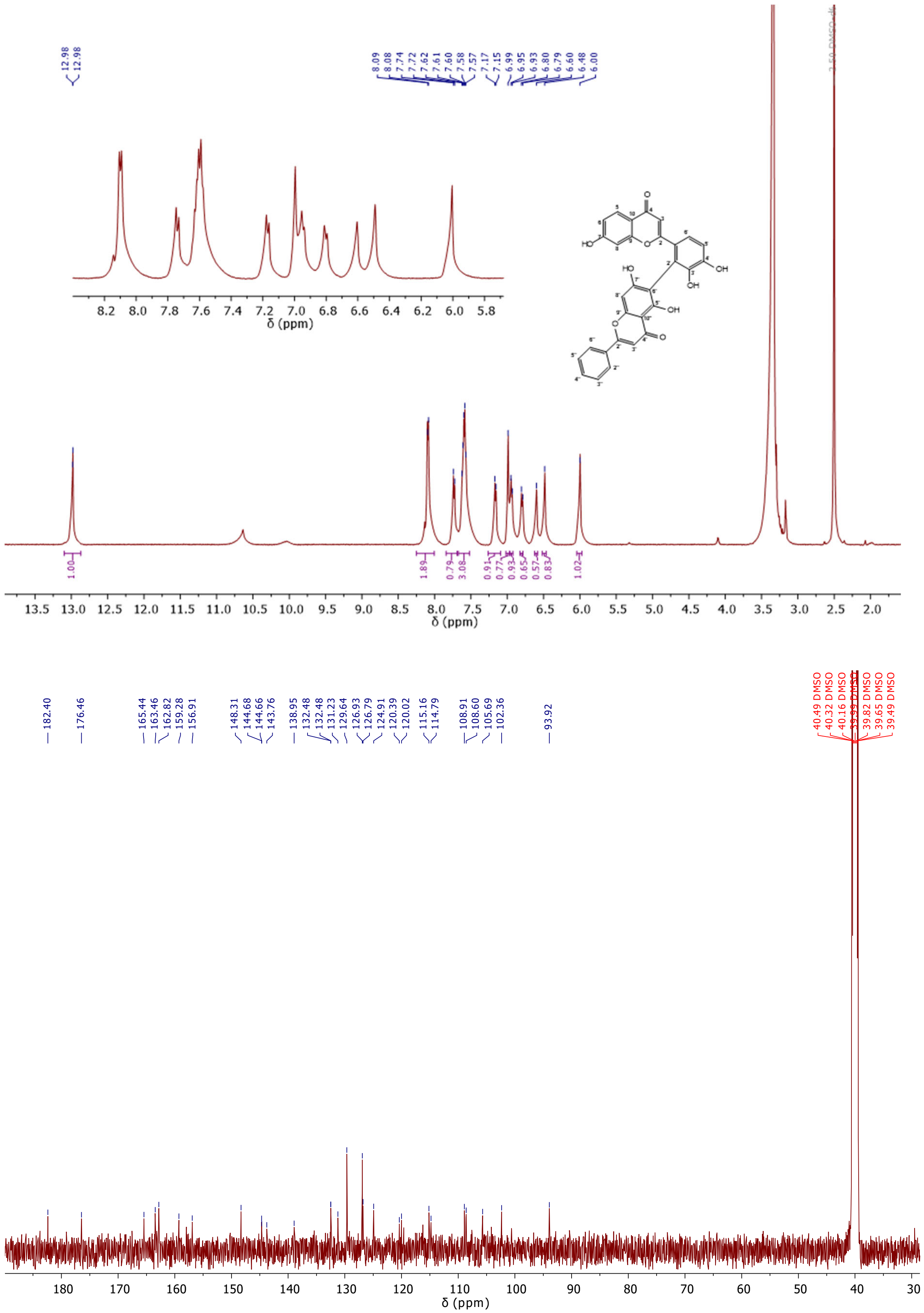

Figure S9.1. ^{1}H and ^{13}C NMR of biflavone 8

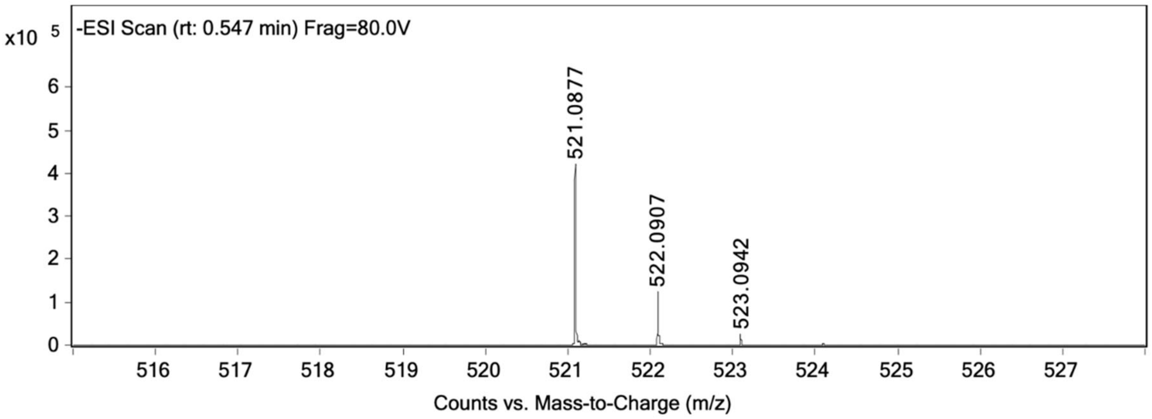

Figure S9.2. ^{1}H and ^{13}C NMR of biflavone 8

9 Acacetin + 3',4'-dihydroxyflavone (6-(2,3-dihydroxy-6-(4-oxo-4H-chromen-2-yl)phenyl)-5,7-dihydroxy-2-(4-methoxyphenyl)-4H-chromen-4-one), 3.25 mg, 3% yield, yellow solid. Semi-prep HPLC, injection volume was 250 µL. ACN/Water = 45: 55, flow rate 5.0 mL/min, λ = 300 nm, ^{1}H NMR (500 MHz, DMSO-*d6*) δ 13.08 (s, 1H), 10.77 (s, 1H), 10.12 (s, 1H), 8.46 (s, 1H), 8.04 (d, *J* = 8.5 Hz, 2H), 7.91 (d, *J* = 7.8 Hz, 1H), 7.68 (t, *J* = 7.8 Hz, 1H), 7.38 (t, *J* = 7.5 Hz, 1H), 7.27 (d, *J* = 8.4 Hz, 1H), 7.22 (d, *J* = 8.3 Hz, 1H), 7.10 (d, *J* = 8.5 Hz, 2H), 6.96 (d, *J* = 8.3 Hz, 1H), 6.87 (s, 1H), 6.59 (s, 1H), 6.12 (s, 1H), 3.85 (s, 3H).^{13}C NMR (126 MHz, DMSO) δ 182.3, 177.0, 166.1, 163.6, 162.8, 162.5, 159.3, 156.8, 156.2, 148.6, 144.7, 134.5, 128.8, 125.7, 125.1, 124.7, 123.4, 123.3, 120.6, 120.2, 118.3, 115.1, 114.8, 109.0, 108.62, 104.0, 93.8, 56.0. HRMS(ESI-TOF) [**M-H**]$^{-}$ calculated for $C_{31}H_{19}O_9$ = 535.1035, found 535.1035.

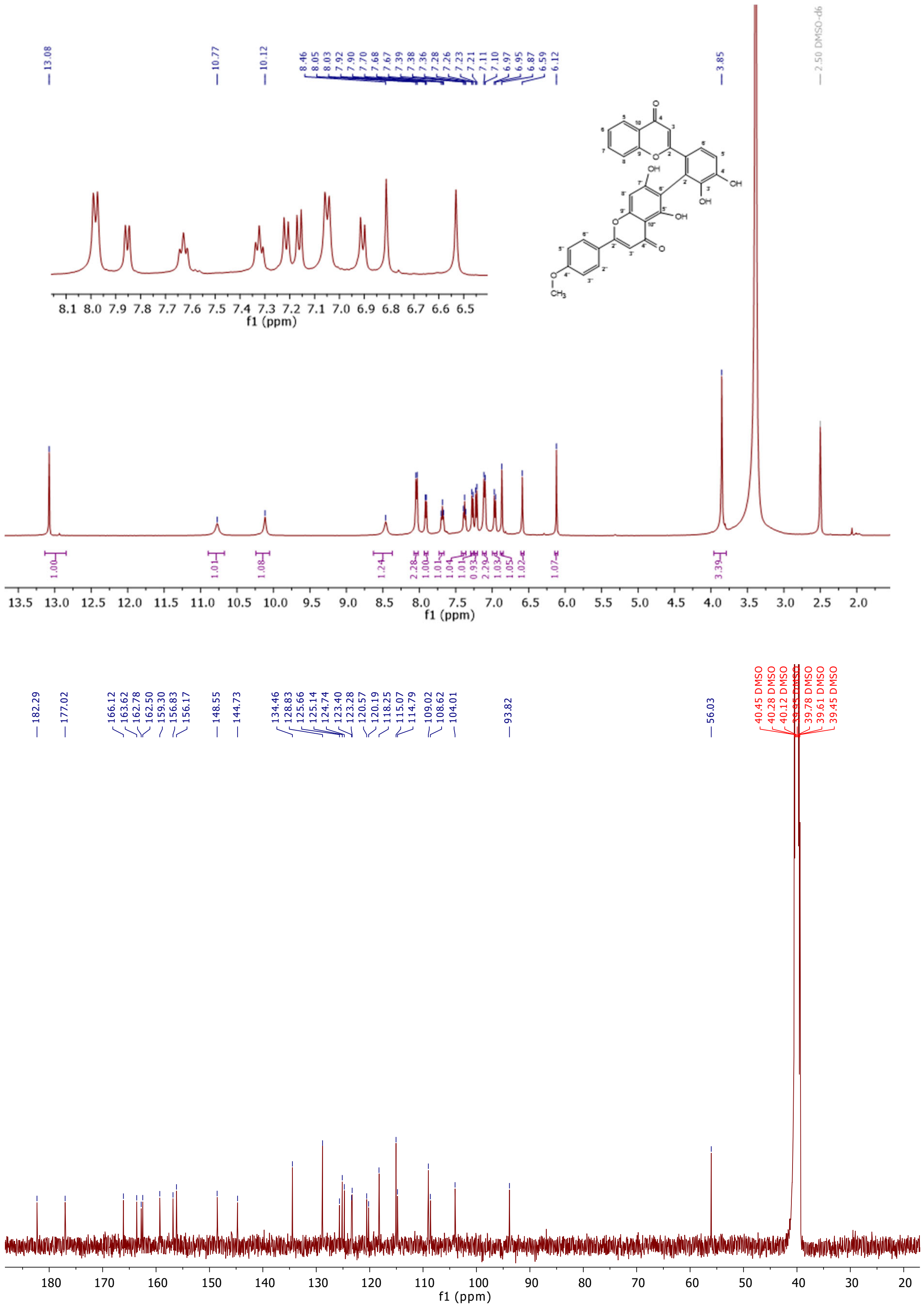

Figure S10.1. ^{1}H and ^{13}C NMR of biflavone 9

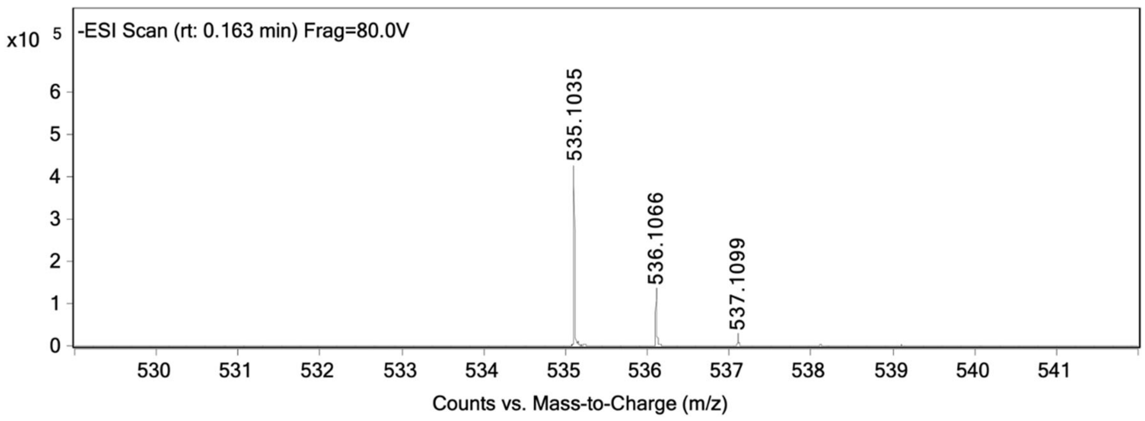

Figure S10.2. HRMS spectrum of biflavone 9

10 Acacetin + 5,3',4'-trihydroxyflavone (6-(2,3-dihydroxy-6-(5-hydroxy-4-oxo-4H-chromen-2-yl)phenyl)-5,7-dihydroxy-2-(4-methoxyphenyl)-4H-chromen-4-one), 11.33 mg, 9% yield, yellow solid. Semi-prep HPLC, injection volume was 250 µL. ACN/Water = 40: 60, flow rate 5.0 mL/min, λ = 300 nm, ^{1}H NMR (500 MHz, DMSO-*d6*) δ 13.10 (s, 1H), 12.62 (s, 1H), 8.04 (d, *J* = 8.5 Hz, 2H), 7.52 (dd, *J* = 8.3 Hz), 7.23 (d, *J* = 8.4 Hz, 1H), 7.10 (d, *J* = 8.5 Hz, 2H), 6.97 (d, *J* = 8.4 Hz, 1H), 6.87 (s, 1H), 6.69 (d, *J* = 8.2 Hz, 1H), 6.65 (d, *J* = 8.4 Hz, 1H), 6.60 (s, 1H), 6.19 (s, 1H), 3.85 (s, 3H). ^{13}C NMR (126 MHz, DMSO) δ 183.0, 182.3, 167.9, 163.6, 162.8, 162.5, 160.3, 159.3, 156.9, 156.4, 149.1, 144.9, 136.2, 128.8, 124.0, 123.3, 121.0, 120.3, 115.1, 114.8, 111.1, 110.1, 108.5, 107.4, 107.2, 104.0, 104.0, 93.9, 56.0. HRMS(ESI-TOF) [**M-H**]$^{-}$ calculated for $C_{31}H_{19}O_{10}$ = 551.0984, found 551.0981.

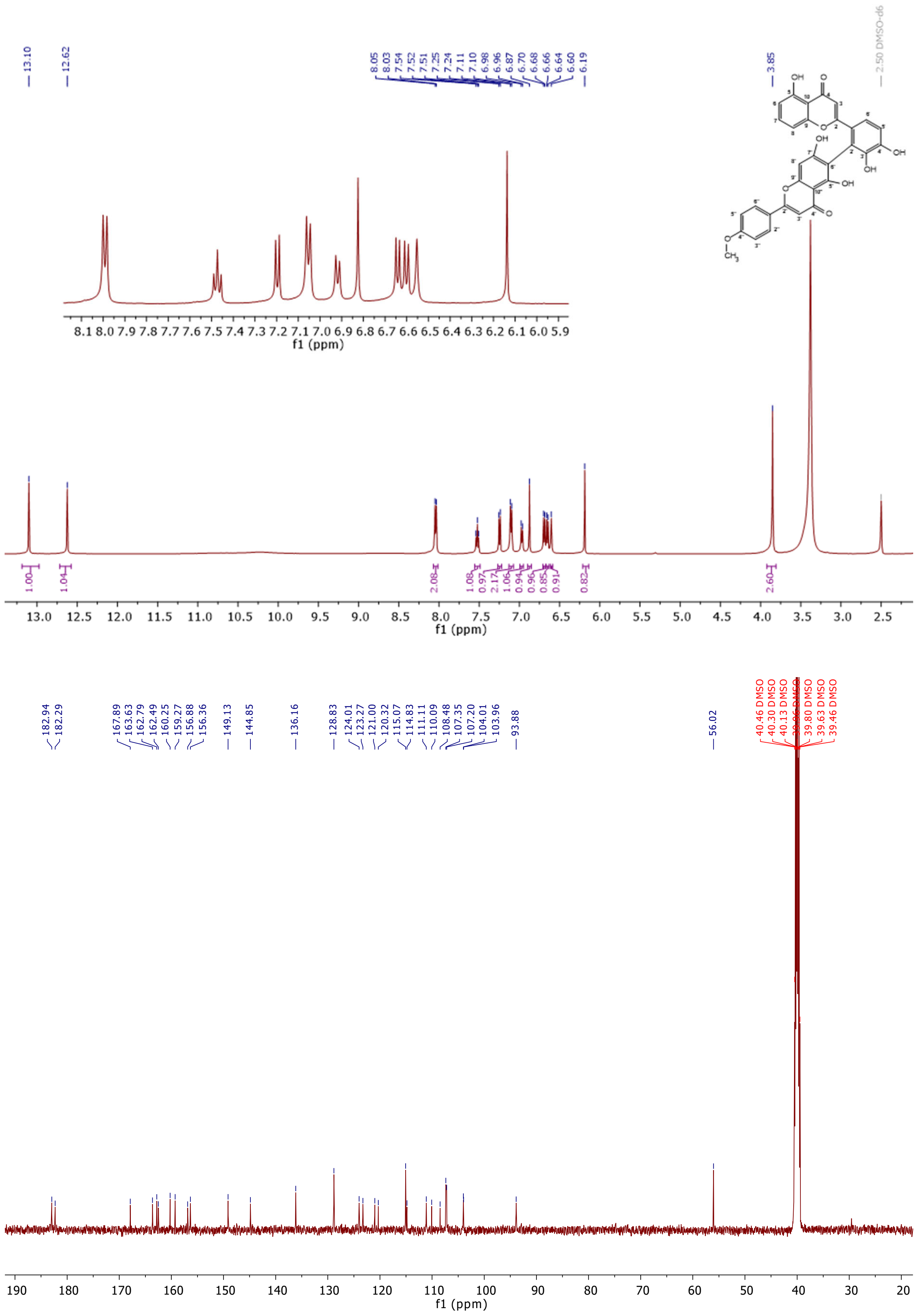

Figure S11.1. ^{1}H and ^{13}C NMR of biflavone 10

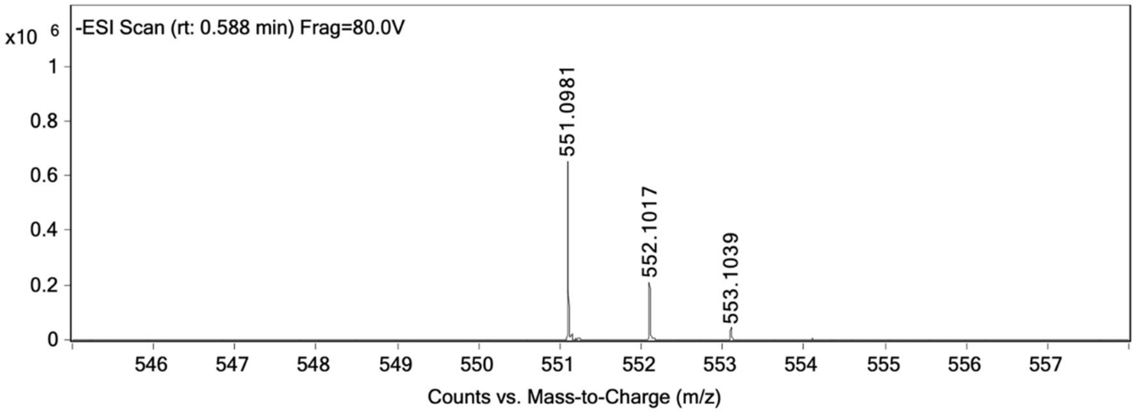

Figure S11.2. ^{1}H and ^{13}C NMR of biflavone 10

11 Acacetin + 6,3',4'-trihydroxyflavone (6-(2,3-dihydroxy-6-(6-hydroxy-4-oxo-4H-chromen-2-yl)phenyl)-5,7-dihydroxy-2-(4-methoxyphenyl)-4H-chromen-4-one), 13.37 mg, 10% yield, yellow solid. Semi-prep HPLC, injection volume was 250 µL. ACN/Water = 35: 65, flow rate 5.0 mL/min, λ = 300 nm, ^{1}H NMR (500 MHz, DMSO-*d6*) δ 13.06 (s, 1H), 8.04 (d, *J* = 8.6 Hz, 2H), 7.18 – 7.14 (m, 3H), 7.12 – 7.10 (m, 3H), 6.94 (d, *J* = 8.3 Hz, 1H), 6.87 (s, 1H), 6.58 (s, 1H), 6.02 (s, 1H), 3.86 (s, 3H). ^{13}C NMR (126 MHz, DMSO-*d6*) δ 182.3, 165.7, 163.6, 162.8, 162.5, 159.3, 156.8, 155.0, 149.9, 148.4, 144.7, 128.8, 125.0, 124.3, 123.3, 120.4, 120.1, 119.6, 115.1, 114.8, 108.1, 107.8, 104.0, 93.8, 56.0. HRMS(ESI-TOF) [**M-H**]$^{-}$ calculated for $C_{31}H_{19}O_{10}$ = 551.0984, found 551.0984.

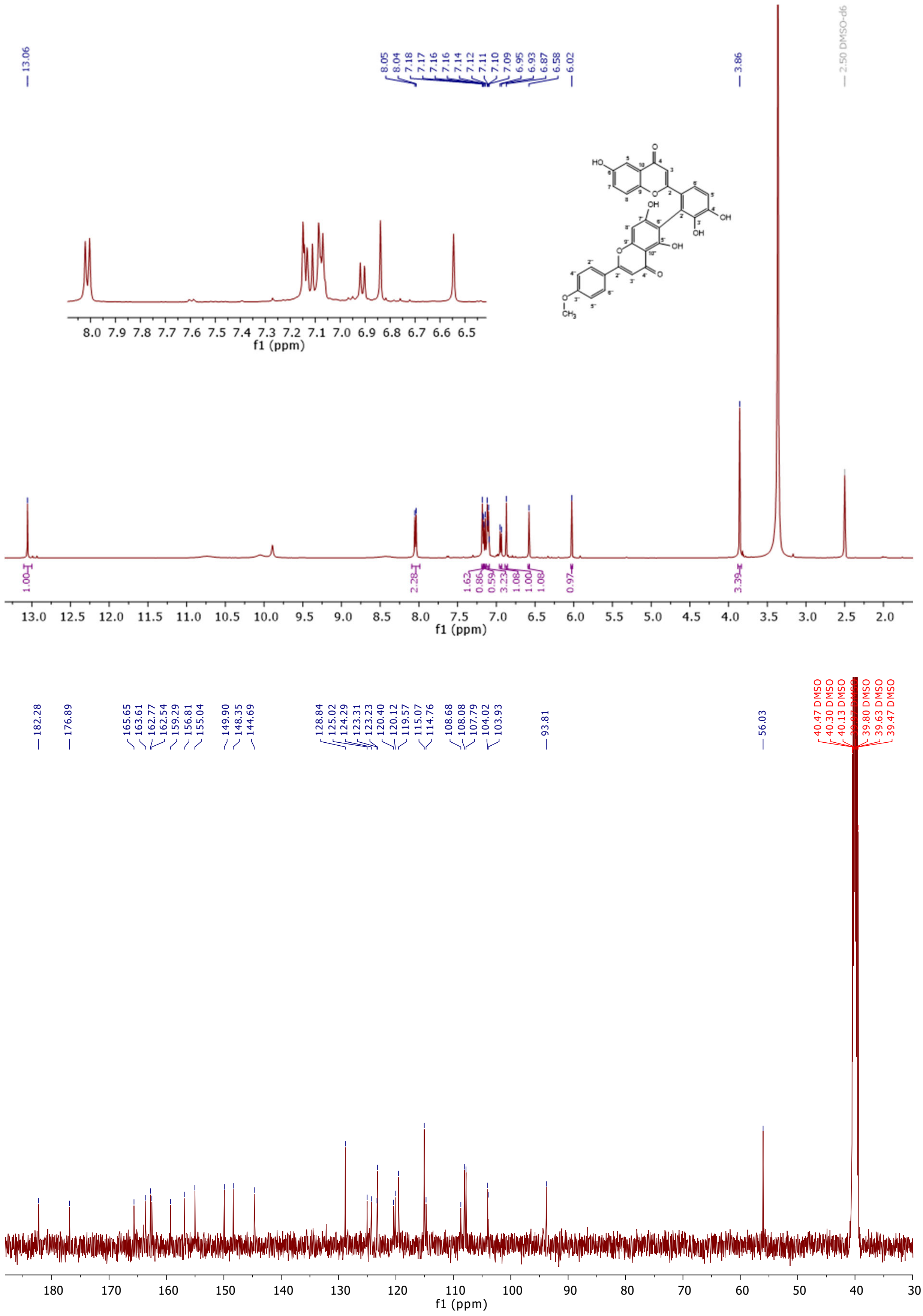

Figure S12.1. ^{1}H and ^{13}C NMR of biflavone 11

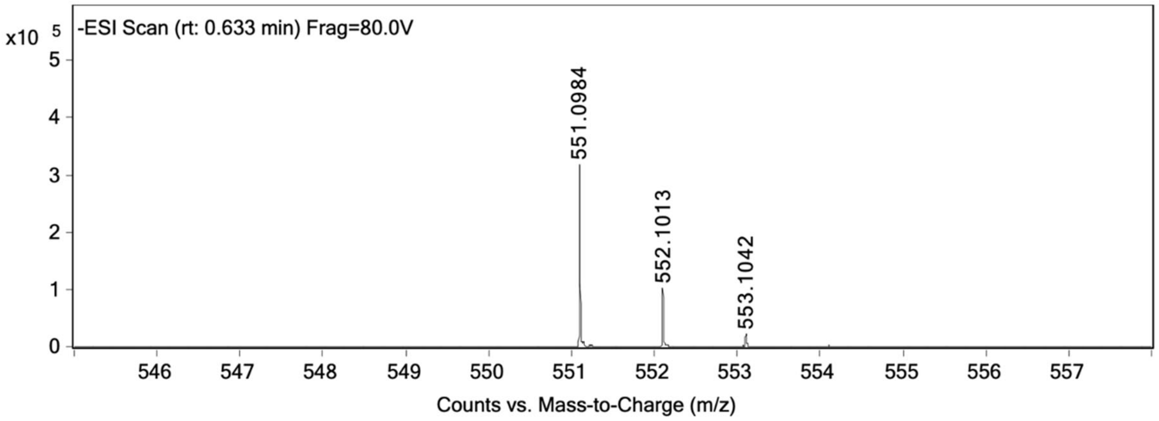

Figure S12.2. HRMS spectrum of biflavone 11

12 Acacetin + 7,3',4'-trihydroxyflavone (6-(2,3-dihydroxy-6-(7-hydroxy-4-oxo-4H-chromen-2-yl)phenyl)-5,7-dihydroxy-2-(4-methoxyphenyl)-4H-chromen-4-one), 4.55 mg, 4% yield, yellow solid. Semi-prep HPLC, injection volume was 250 µL. ACN/Water = 35: 65, flow rate 5.0 mL/min, λ = 300 nm, ^{1}H NMR (500 MHz, DMSO-*d6*) δ 13.06 (s, 1H), 8.05 (d, *J* = 8.7 Hz, 2H), 7.73 (d, *J* = 8.6 Hz, 1H), 7.16 (d, *J* = 8.4 Hz, 1H), 7.11 (d, *J* = 8.7 Hz, 2H), 6.94 (d, *J* = 8.4 Hz, 1H), 6.87 (s, 1H), 6.79 (dd, *J* = 8.8, 2.2 Hz), 6.58 (s, 1H), 6.47 (d, *J* = 2.2 Hz, 1H), 5.99 (s, 1H), 3.86 (s, 3H). ^{13}C NMR (126 MHz, DMSO) δ 182.31, 176.48, 165.49, 165.44, 163.60, 162.83, 162.77, 162.77, 162.45, 159.27, 157.96, 156.79, 148.28, 144.65, 128.83, 128.83, 126.79, 124.91, 123.33, 120.39, 120.06, 115.17, 115.08, 108.60, 104.0, 103.9, 102.4, 93.8, 56.0. HRMS(ESI-TOF) [**M**-**H**]$^{-}$ calculated for $C_{31}H_{19}O_{10}$ = 551.0984, found 551.0982.

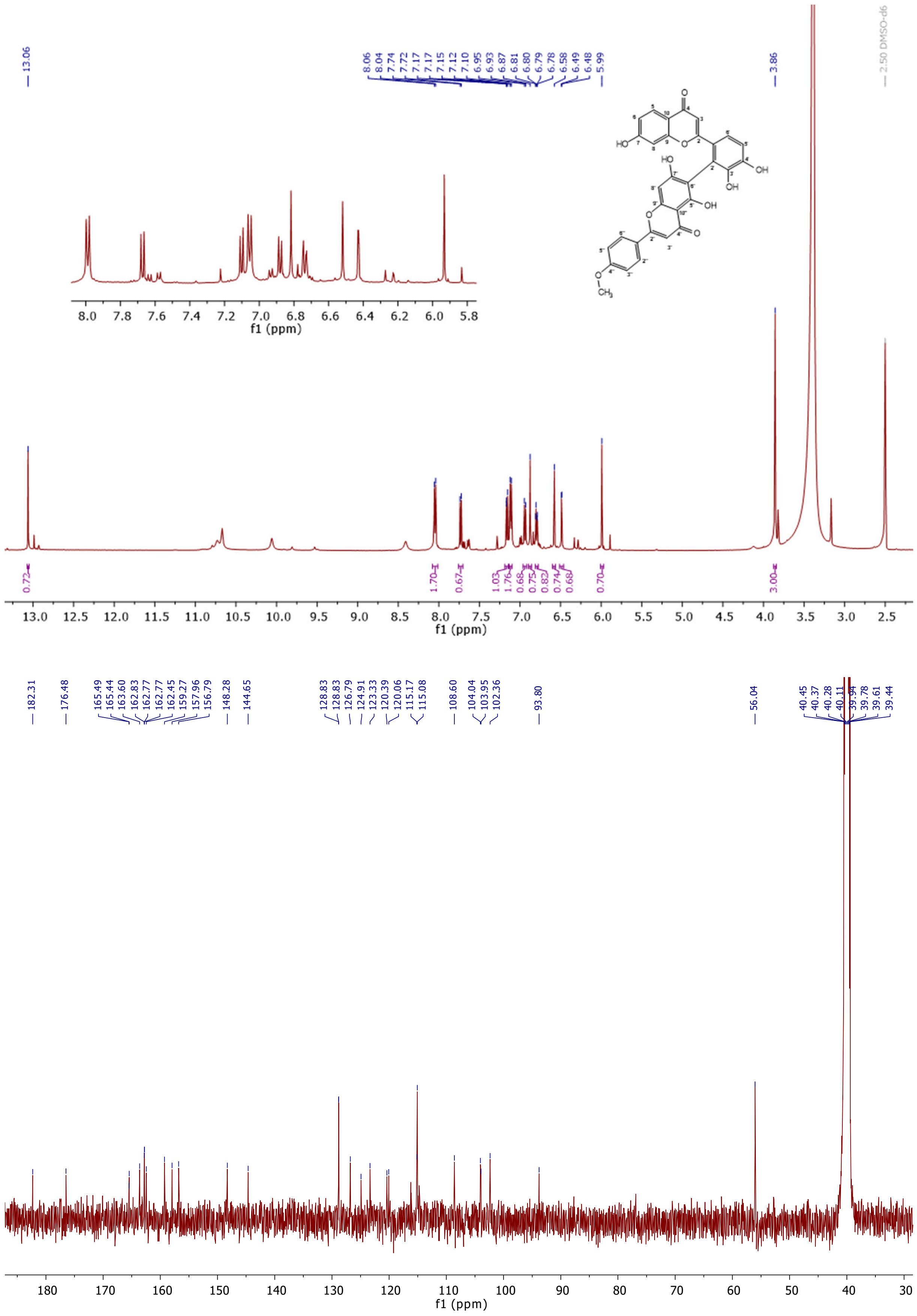

Figure S13.1. ^{1}H and ^{13}C NMR of biflavone 12

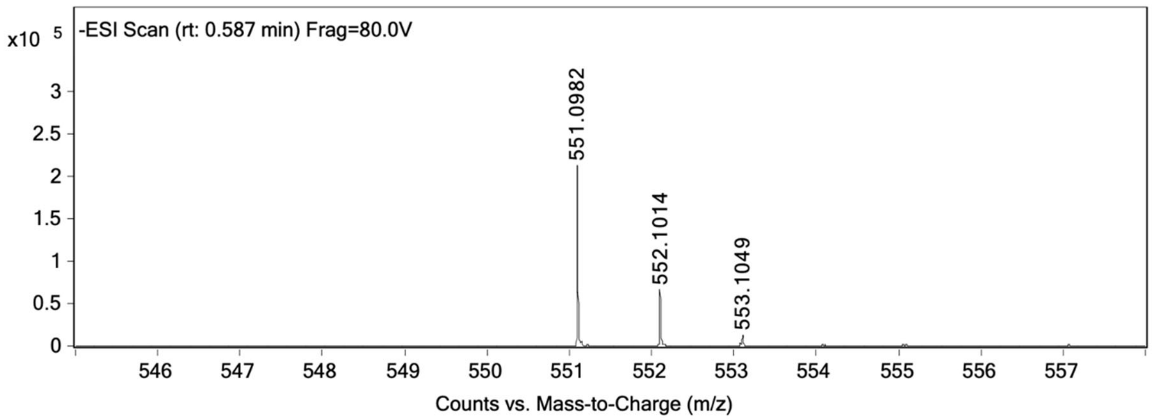

Figure S13.2. ^{1}H and ^{13}C NMR of biflavone 12

13 Genistein + 3',4'-dihydroxyflavone (6-(2,3-dihydroxy-6-(4-oxo-4H-chromen-2-yl)phenyl)-5,7-dihydroxy-3-(4-methoxyphenyl)-4H-chromen-4-one), 10 mg, 7% yield, yellow solid. Semi-prep HPLC, injection volume was 250 µL. ACN/Water = 40: 60, flow rate 5.0 mL/min, λ = 300 nm, ^{1}H NMR (500 MHz, DMSO-*d6*) δ 13.13 (s, 1H), 8.29 (s, 1H), 7.92 (d, *J* = 7.8 Hz, 1H), 7.70 (dd, *J* = 7.8 Hz, 1H), 7.39 (dd, *J* = 7.5 Hz, 1H), 7.35 (d, *J* = 8.2 Hz, 2H), 7.28 (d, *J* = 8.4 Hz, 1H), 7.20 (d, *J* = 8.2 Hz), 6.94 (d, *J* = 8.3 Hz), 6.79 (d, *J* = 8.3 Hz), 6.44 (s, 1H), 6.10 (s, 1H). ^{13}C NMR (126 MHz, DMSO) δ 180.67, 177.04, 175.30, 174.02, 166.17, 165.77, 163.40, 159.70, 157.82, 157.10, 156.17, 156.04, 154.23, 148.65, 134.45, 130.67, 125.14, 123.39, 122.69, 121.73, 120.52, 120.30, 118.28, 115.50, 114.69, 108.92, 108.79, 93.55. HRMS(ESI-TOF) calculated for $C_{30}H_{17}O_9$ = 521.0878, found 521.0877.

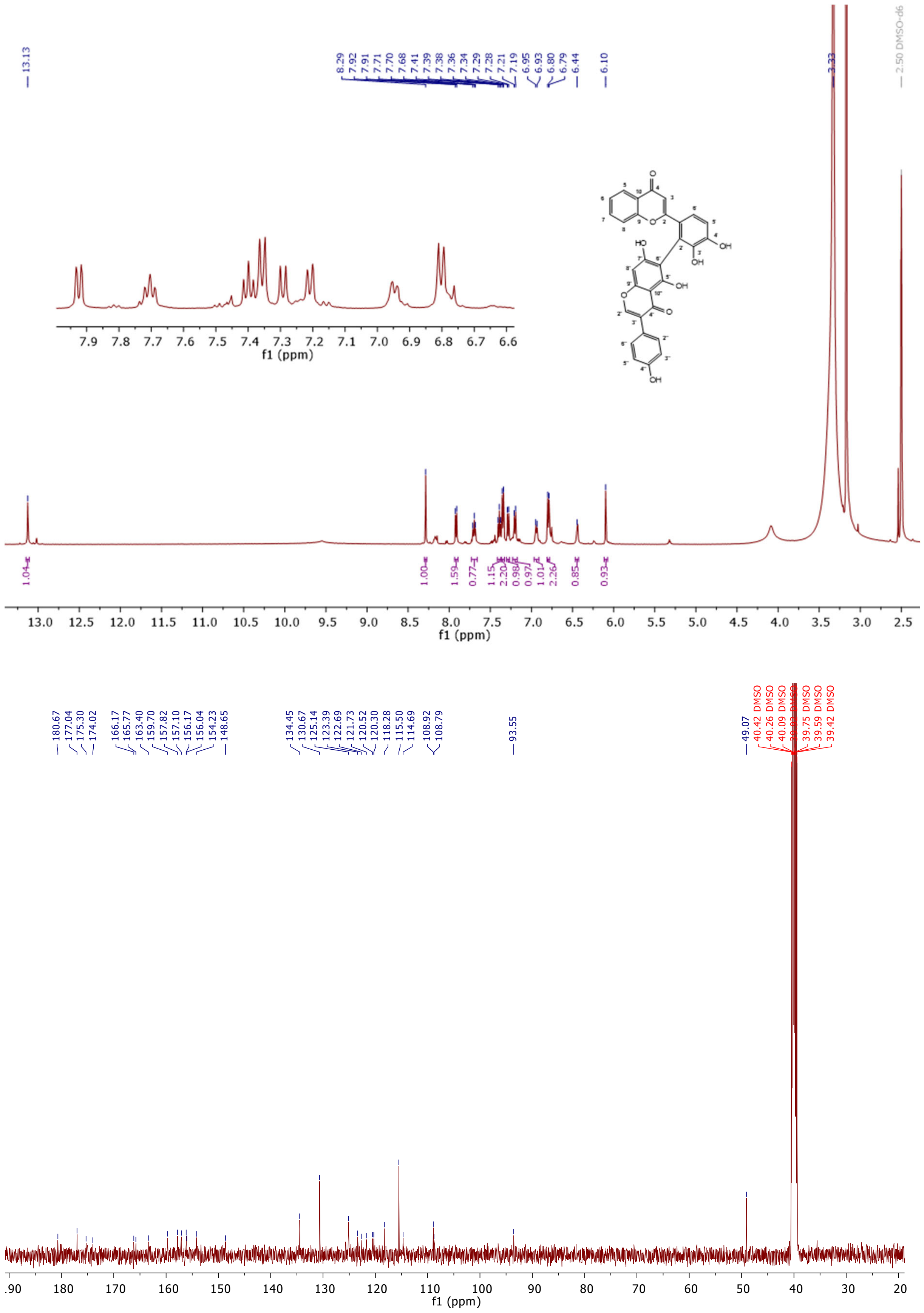

Figure S14.1. ^{1}H and ^{13}C NMR of biflavone 13

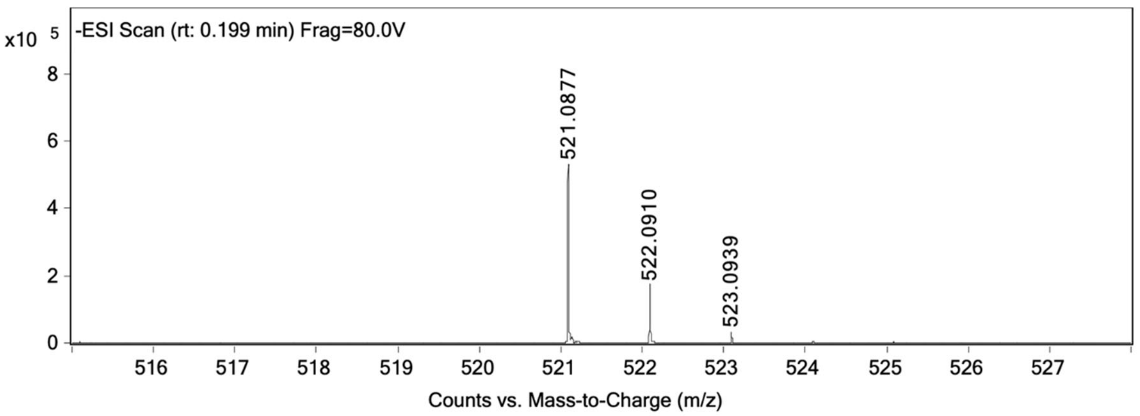

Figure S14.1.HRMS spectrum of biflavone 13

14 Genistein + 5,3',4'-trihydroxyflavone (6-(2,3-dihydroxy-6-(5-hydroxy-4-oxo-4H-chromen-2-yl)phenyl)-5,7-dihydroxy-3-(4-methoxyphenyl)-4H-chromen-4-one), 8.38 mg, 6% yield, yellow solid. Semi-prep HPLC, injection volume was 250 μL. ACN/Water = 40: 60, flow rate 5.0 mL/min, λ = 300 nm, ^{1}H NMR (500 MHz, DMSO-*d6*) δ 13.13 (s, 1H), 12.63 (s, 1H), 10.79 (s, 1H), 10.22 (s, 1H), 9.59 (s, 1H), 8.51 (s, 1H), 8.34 (s, 1H), 7.55 (dd, *J* = 8.3 Hz, 1H), 7.37 (d, *J* = 8.2 Hz, 2H), 7.25 (d, *J* = 8.3 Hz, 1H), 6.97 (d, *J* = 8.4 Hz, 1H), 6.81 (d, *J* = 8.1 Hz, 2H), 6.72 (d, *J* = 8.1 Hz, 1H), 6.66 (d, *J* = 8.3 Hz, 1H), 6.49 (s, 1H), 6.18 (s, 1H). ^{13}C NMR (126 MHz, DMSO) δ 182.9, 180.8, 167.8, 162.6, 160.3, 159.7, 157.9, 157.1, 156.4, 154.4, 149.1, 144.8, 136.2, 130.7, 124.0, 122.8, 121.7, 121.0, 120.3, 115.5, 114.9, 111.1, 110.1, 108.5, 107.3, 107.2, 104.7, 93.5. HRMS(ESI-TOF) [**M**-**H**]$^{-}$ calculated for $C_{30}H_{17}O_{10}$ = 537.0827, found 537.0825.

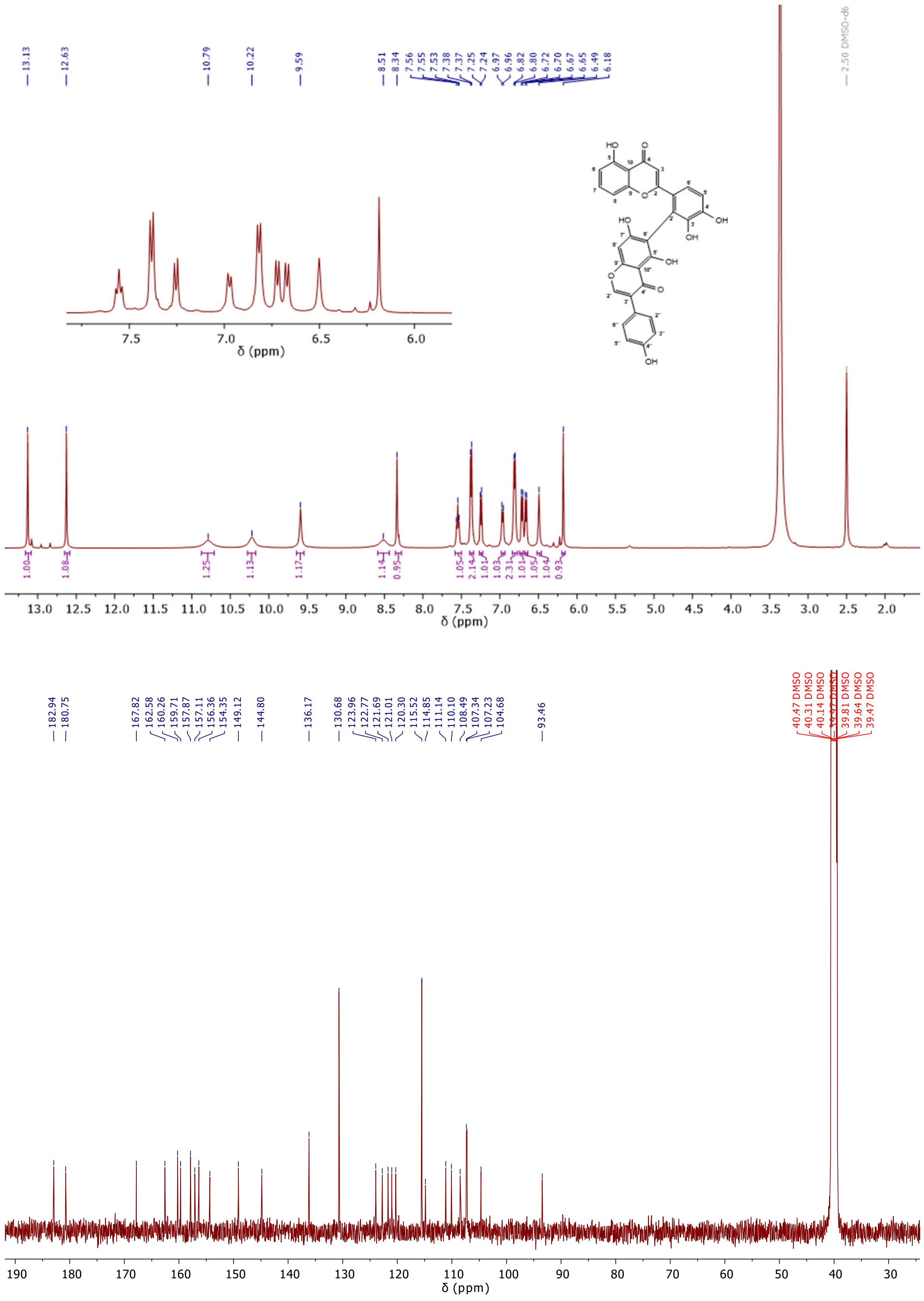

Figure S15.1. ^{1}H and ^{13}C NMR of biflavone 14

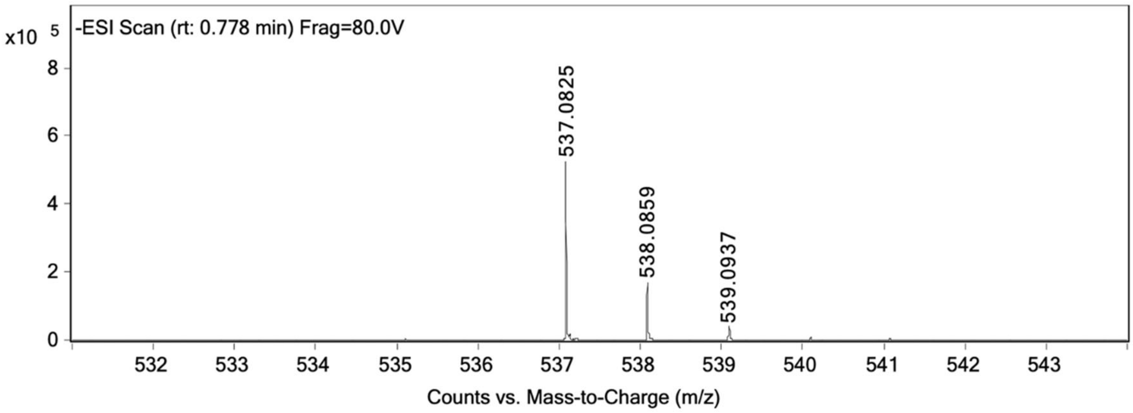

Figure S15.2. HRMS spectrum of biflavone 14

15 Genistein + 6,3',4'-trihydroxyflavone (6-(2,3-dihydroxy-6-(6-hydroxy-4-oxo-4H-chromen-2-yl)phenyl)-5,7-dihydroxy-3-(4-methoxyphenyl)-4H-chromen-4-one), 17 mg, 13% yield, yellow solid. Semi-prep HPLC, injection volume was 250 µL. ACN/Water = 40: 60, flow rate 5.0 mL/min, λ = 300 nm, ^{1}H NMR (500 MHz, DMSO-*d6*) δ 13.09 (s, 1H), 10.77 (s, 1H), 10.08 (s, 1H), 9.93 (s, 1H), 9.61 (s, 1H), 8.43 (s, 1H), 8.32 (s, 1H), 7.38 (d, *J* = 8.2 Hz, 2H), 7.17 (m, 3H), 6.95 (d, *J* = 8.4 Hz, 1H), 6.82 (d, *J* = 8.1 Hz, 2H), 6.48 (s, 1H), 6.00 (s, 1H). ^{13}C NMR (126 MHz, DMSO) δ 180.7, 176.9, 165.6, 162.7, 159.7, 157.9, 157.1, 155.1, 154.3, 149.9, 148.4, 144.7, 132.7, 131.7, 125.0, 124.3, 123.3, 122.8, 121.7, 120.4, 120.1, 119.6, 115.9, 115.5, 114.8, 108.7, 108.1, 107.8, 104.6, 93.4. HRMS(ESI-TOF) [**M**-**H**]$^{-}$ calculated for $C_{30}H_{17}O_{10}$ = 537.0827, found 537.0826.

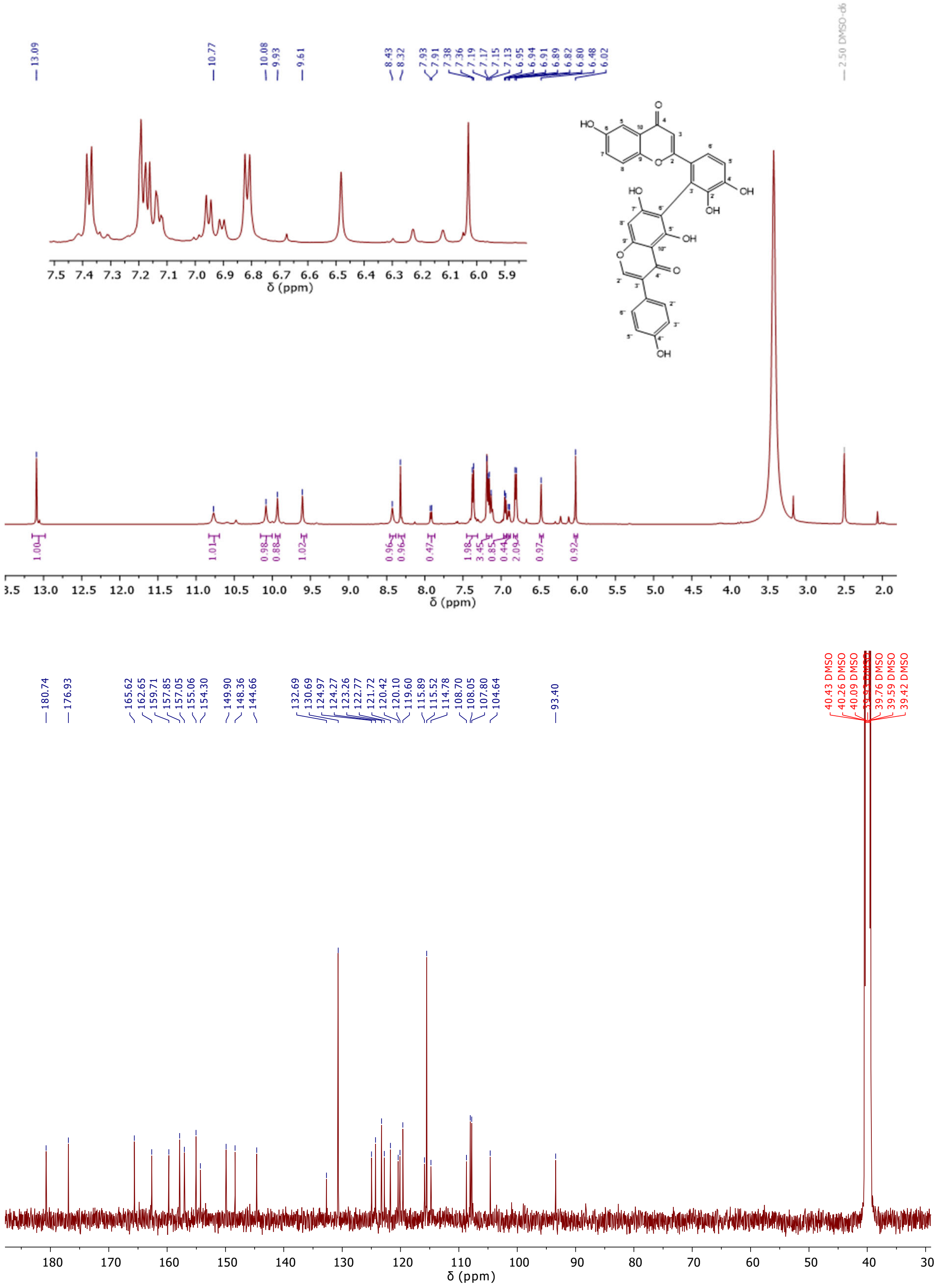

Figure S16.1. ^{1}H and ^{13}C NMR of biflavone 15

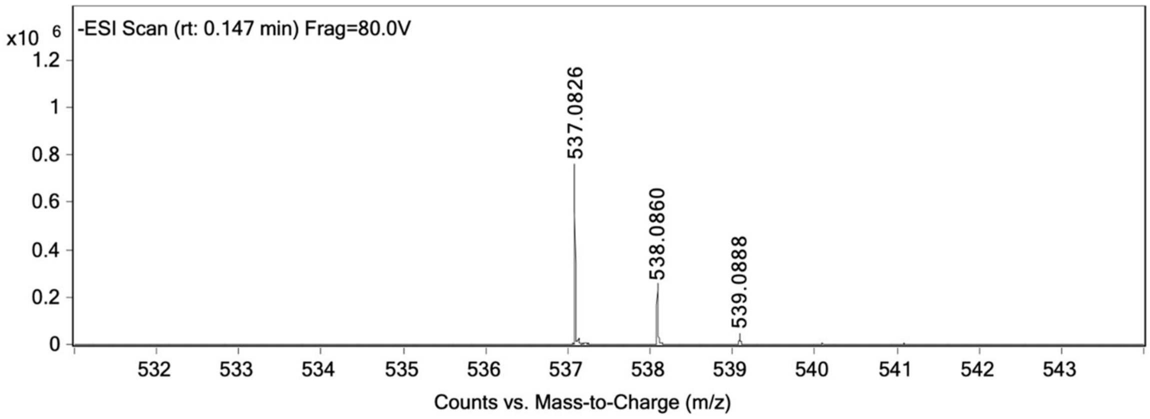

Figure S16.2. HRMS spectrum of biflavone 15

16 Genistein + 7,3',4'-trihydroxyflavone (6-(2,3-dihydroxy-6-(7-hydroxy-4-oxo-4H-chromen-2-yl)phenyl)-5,7-dihydroxy-3-(4-methoxyphenyl)-4H-chromen-4-one), 15.3 mg, 11% yield, yellow solid. Semi-prep HPLC, injection volume was 250 µL. ACN/Water = 35: 65, flow rate 5.0 mL/min, λ = 300 nm, ^{1}H NMR (500 MHz, DMSO-*d6*) δ 13.11 (s, 1H), 10.75 (s, 1H), 10.70 (s, 1H), 10.07 (s, 1H), 9.61 (s, 1H), 8.41 (s, 1H), 8.32 (s, 1H), 7.75 (d, *J* = 8.7 Hz, 1H), 7.38 (d, *J* = 8.5 Hz, 2H), 7.16 (d, *J* = 8.3 Hz, 1H), 6.94 (d, *J* = 8.3 Hz, 1H), 6.83 (d, J=8.3 Hz, 1H), 6.81 (d, J=8.6 Hz, 2H), 6.51 (d, *J* = 2.2 Hz, 1H), 6.47 (s, 1H), 5.99 (s, 1H). ^{13}C NMR (126 MHz, DMSO) δ 180.8, 176.5, 165.4, 162.9, 162.6, 159.7, 158.0, 157.9, 157.0, 154.3, 148.3, 144.6, 132.7, 130.7, 126.8, 124.9, 122.8, 121.8, 120.4, 120.1, 116.2, 115.5, 115.2, 114.8, 108.7, 108.6, 104.7, 102.4, 93.4. HRMS(ESI-TOF) [**M**-**H**]$^-$ calculated for $C_{30}H_{17}O_{10}$ = 537.0827, found 537.0825.

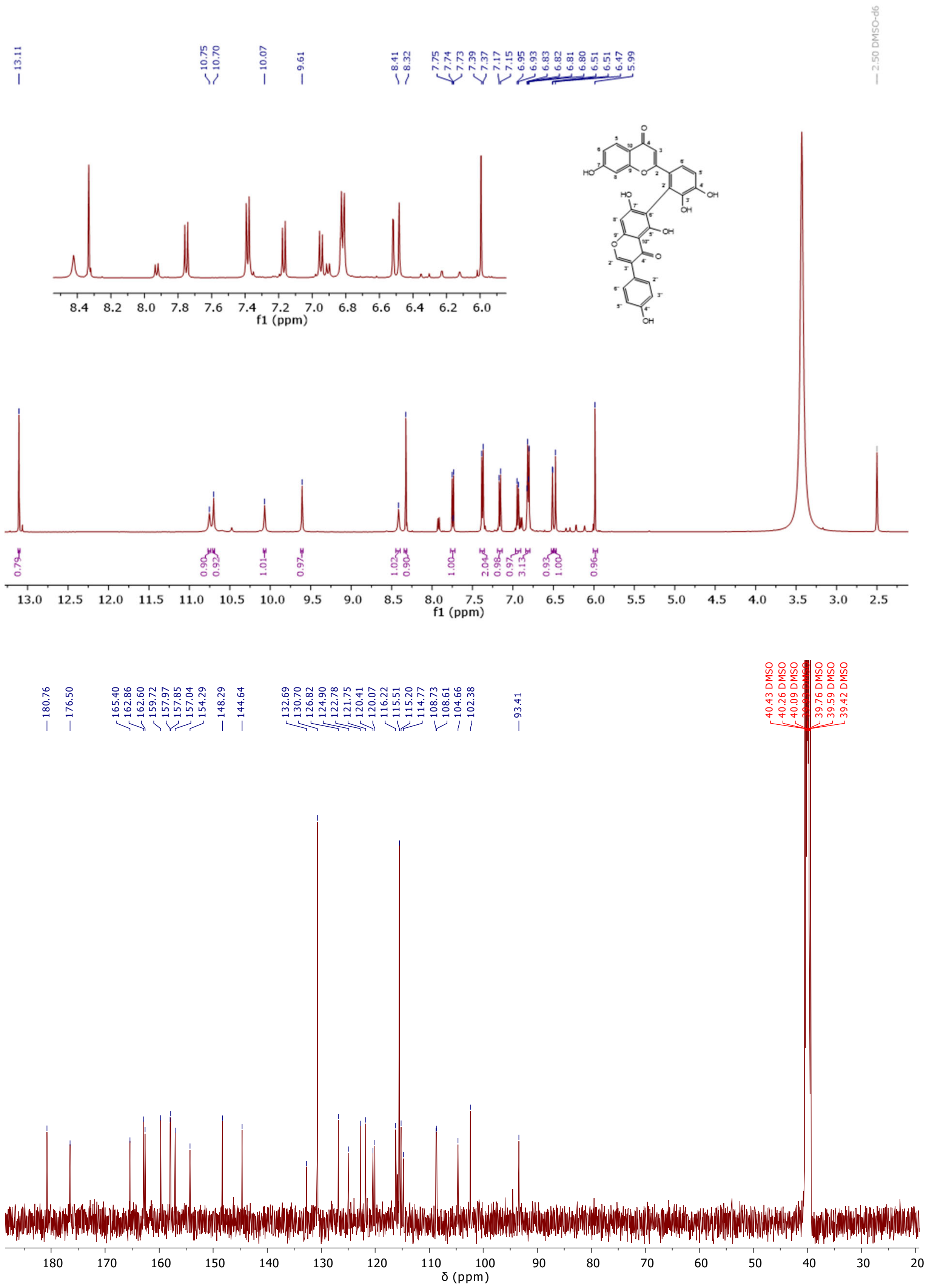

Figure S17.1. ^{1}H and ^{13}C NMR of biflavone 16

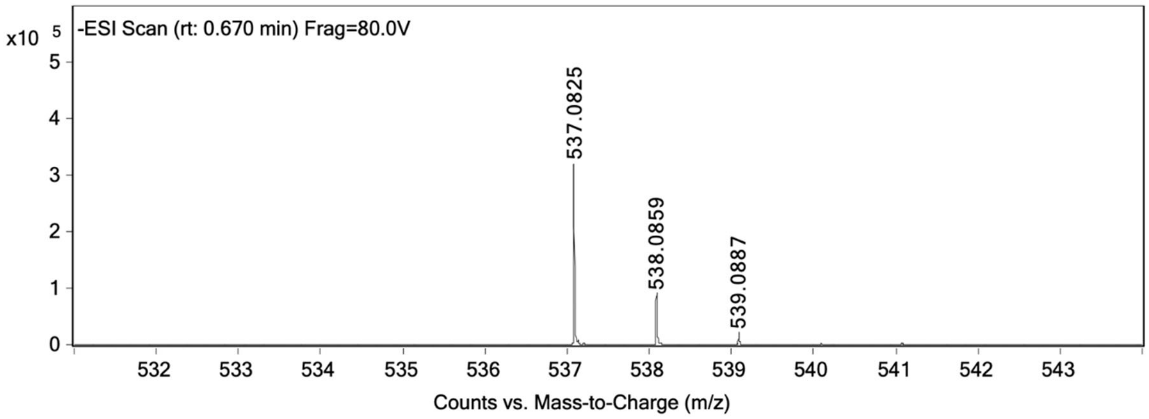

Figure S17.2. ^{1}H and ^{13}C NMR of biflavone 16

17 Biochanin A + 3',4'-dihydroxyflavone (6-(2,3-dihydroxy-6-(4-oxo-4H-chromen-2-yl)phenyl)-5,7-dihydroxy-3-(4-hydroxyphenyl)-4H-chromen-4-one), 13 mg, 10% yield, yellow solid. Semi-prep HPLC, injection volume was 250 µL. ACN/Water = 40: 60, flow rate 5.0 mL/min, λ = 300 nm, ^{1}H NMR (500 MHz, DMSO-*d6*) δ 13.22 – 12.93 (m, 1H), 8.37 (s, 1H), 7.92 (d, *J* = 7.8 Hz, 1H), 7.70 (dd, *J* = 7.9 Hz, 1H), 7.49 (d, *J* = 8.4 Hz, 2H), 7.40 (dd, *J* = 7.5 Hz, 1H), 7.30 (d, *J* = 8.4 Hz, 1H), 7.23 (d, *J* = 8.7 Hz, 1H), 7.01 – 6.96 (m, 3H), 6.49 (s, 1H), 6.12 (s, 1H), 3.78 (s, 3H). ^{13}C NMR (126 MHz, DMSO) δ 180.6, 177.0, 166.1, 162.8, 159.7, 159.6, 157.1, 156.2, 154.6, 148.6, 144.7, 134.5, 130.7, 130.6, 125.7, 125.2, 124.7, 123.4, 122.4, 120.6, 120.2, 118.3, 114.8, 114.2, 109.0, 108.7, 104.6, 93.5, 55.6. HRMS(ESI-TOF) [**M-H**]$^-$ calculated for $C_{31}H_{19}O_9$ = 535.103, found 535.1033.

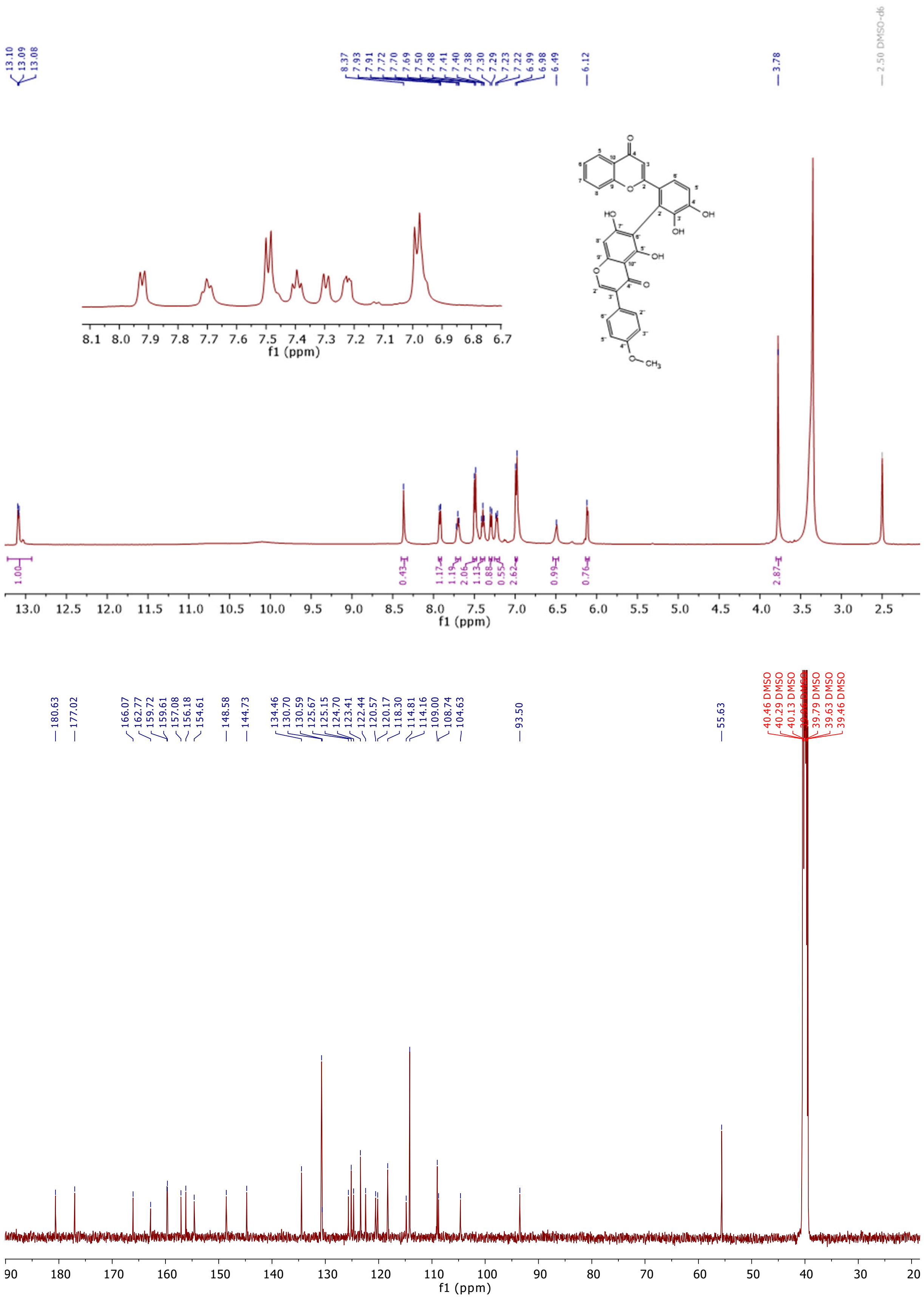

Figure S18.1. ^{1}H and ^{13}C NMR of biflavone 17

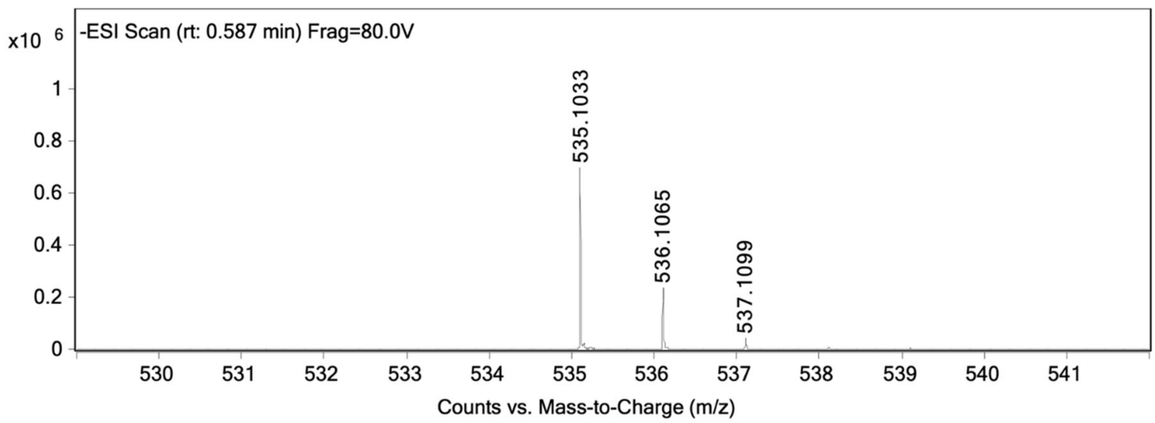

Figure S18.2. HRMS spectrum of biflavone 17

18 Biochanin A + 5,3',4'-trihydroxyflavone (6-(2,3-dihydroxy-6-(5-hydroxy-4-oxo-4H-chromen-2-yl)phenyl)-5,7-dihydroxy-3-(4-hydroxyphenyl)-4H-chromen-4-one), 5.47 mg, 4% yield, yellow solid. Semi-prep HPLC, injection volume was 250 µL. ACN/Water = 45: 55, flow rate 5.0 mL/min, λ = 300 nm, ^{1}H NMR (500 MHz, DMSO-*d6*) δ 13.10 (s, 1H), 12.62 (s, 1H), 8.37 (s, 1H), 7.55 (dd, *J* = 8.3 Hz, 1H), 7.49 (d, *J* = 8.2 Hz, 2H), 7.24 (d, *J* = 8.3 Hz, 1H), 6.99 (d, *J* = 8.4 Hz, 2H), 6.96 (d, *J* = 7.9 Hz, 1H), 6.71 (d, *J* = 8.2 Hz, 1H), 6.67 (d, *J* = 8.3 Hz, 1H), 6.50 (s, 1H), 6.18 (s, 1H), 3.78 (s, 3H). ^{13}C NMR (126 MHz, DMSO) δ 182.9, 180.6, 167.8, 162.7, 160.3, 159.7, 159.6, 157.1, 156.4, 154.7, 149.1, 144.8, 136.2, 130.7, 124.0, 123.4, 122.5, 121.0, 120.3, 114.9, 114.2, 111.2, 110.1, 108.6, 107.3, 107.2, 104.7, 93.5, 55.6. HRMS(ESI-TOF) [**M-H**]$^-$ calculated for $C_{31}H_{19}O_{10}$ = 551.0980, found 551.0981.

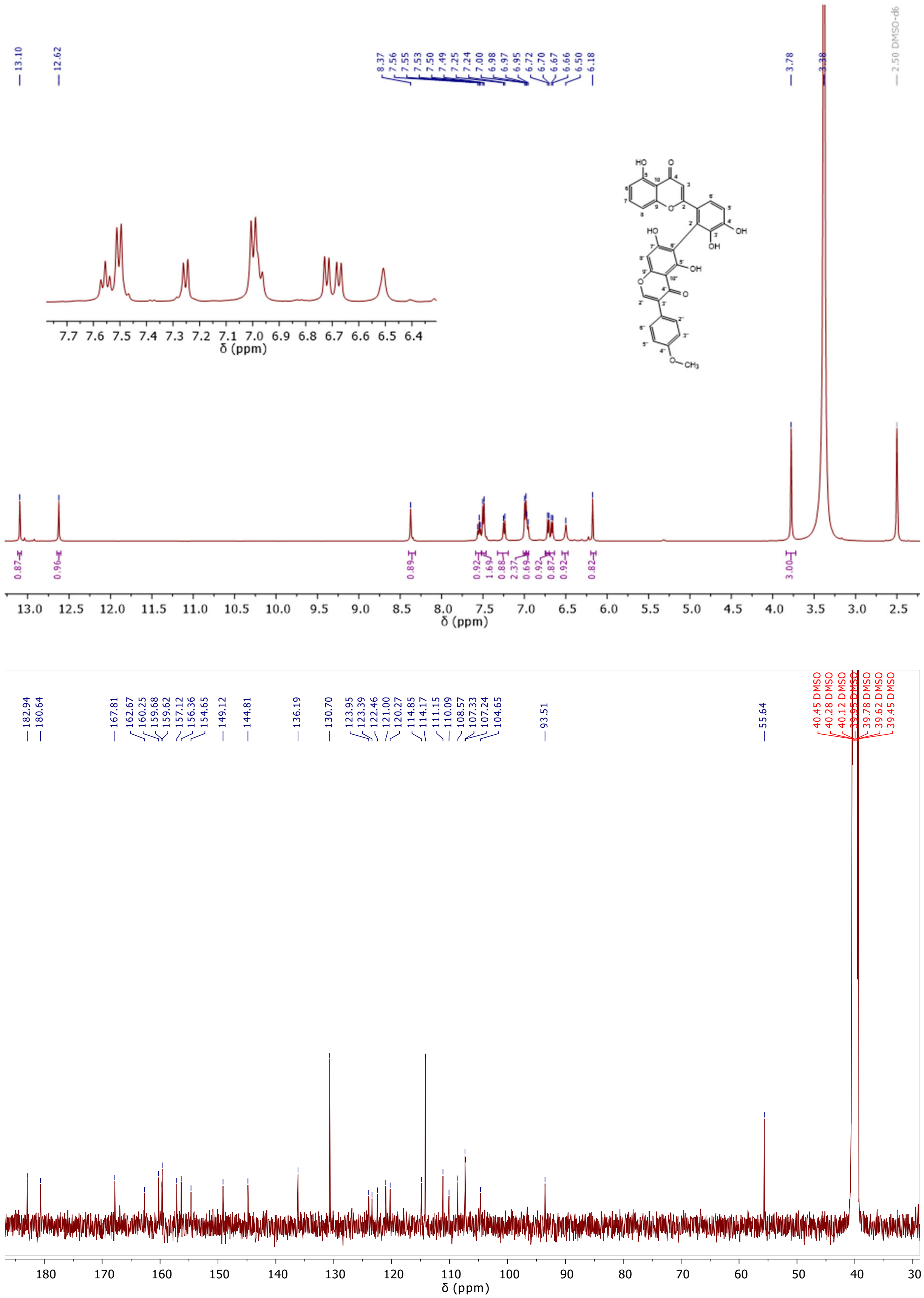

Figure S19.1. ^{1}H and ^{13}C NMR of biflavone 18

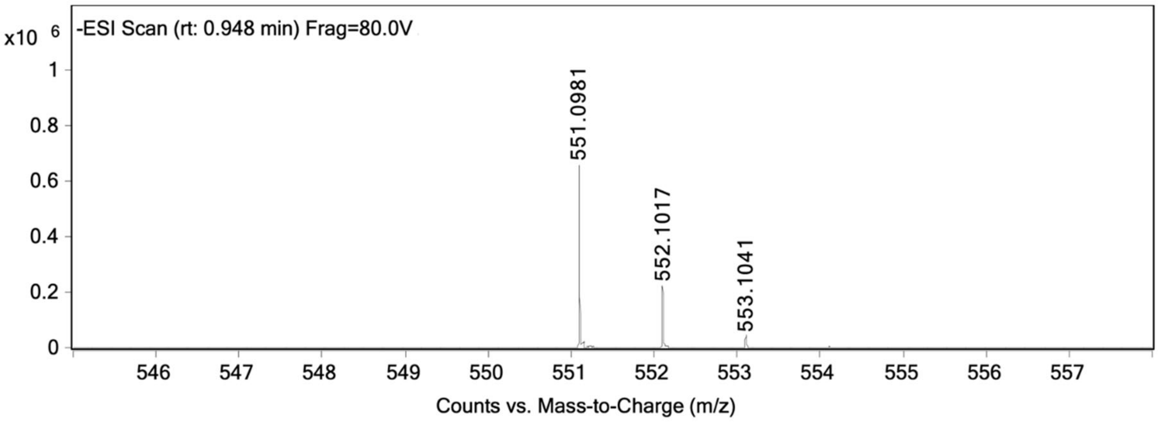

Figure S19.2. HRMS spectrum of biflavone 18

19 Biochanin A + 6,3',4'-trihydroxyflavone (6-(2,3-dihydroxy-6-(6-hydroxy-4-oxo-4H-chromen-2-yl)phenyl)-5,7-dihydroxy-3-(4-hydroxyphenyl)-4H-chromen-4-one), 19.29 mg, 14% yield, yellow solid. Semi-prep HPLC, injection volume was 250 µL. ACN/Water = 40: 60, flow rate 5.0 mL/min, λ = 300 nm, ^{1}H NMR (500 MHz, DMSO-*d6*) δ 13.08 – 12.89 (m, 1H), 10.79 (s, 1H), 10.09 (s, 1H), 9.94 (d, *J* = 7.3 Hz, 1H), 8.43 (s, 1H), 8.37 (s, 1H), 7.49 (d, *J* = 8.6 Hz, 2H), 7.21 – 7.14 (m, 3H), 7.12 (dd, *J* = 8.6, 1.8 Hz), 6.99 (d, *J* = 8.6 Hz, 2H), 6.94 (d, *J* = 8.4 Hz, 1H), 6.48 (s, 1H), 6.03 (s, 1H), 3.77 (d, *J* = 2.4 Hz, 3H). ^{13}C NMR (126 MHz, DMSO) δ 180.6, 176.9, 165.6, 162.7, 159.7, 159.6, 157.1, 155.1, 154.6, 149.9, 148.4, 144.7, 130.7, 125.0, 124.3, 123.4, 123.3, 122.5, 120.4, 120.1, 119.6, 114.8, 114.2, 108.8, 108.1, 107.8, 104.6, 93.5, 55.6. HRMS(ESI-TOF) [**M**-**H**]$^-$ calculated for $C_{31}H_{19}O_{10}$ = 551.0984, found 551.0982.

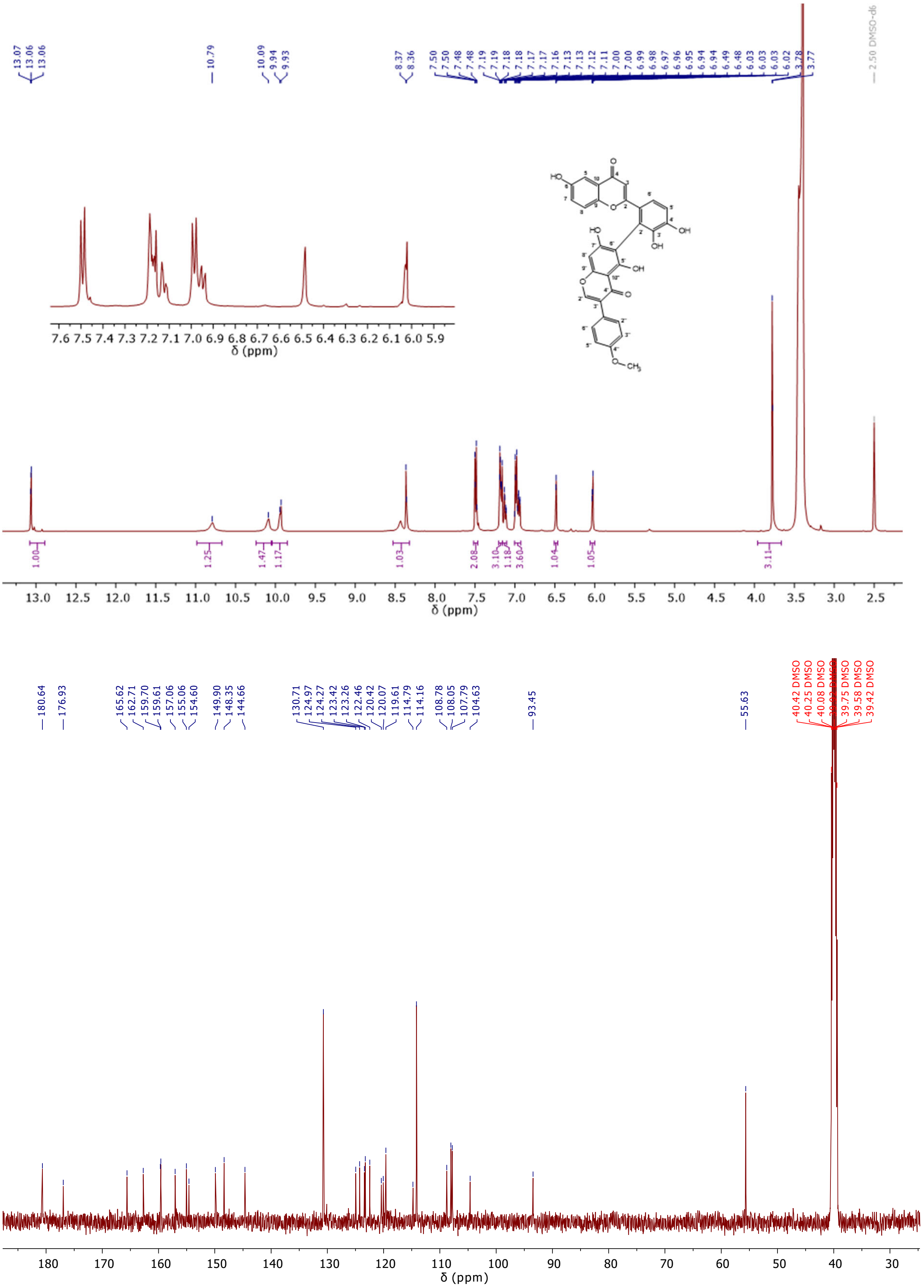

Figure S20.1. ^{1}H and ^{13}C NMR of biflavone 19

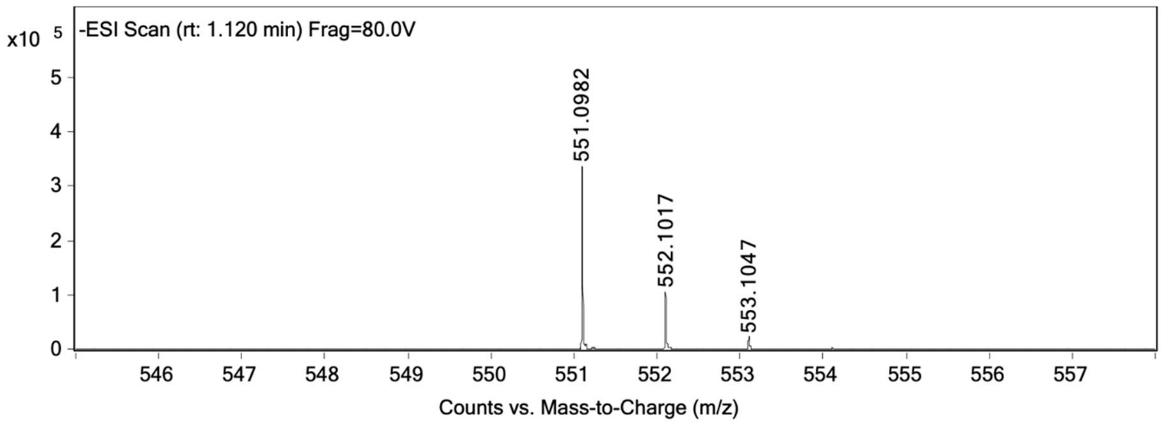

Figure S20.2. ^{1}H and ^{13}C NMR of biflavone 19

20 Biochanin A + 7,3',4'-trihydroxyflavone (6-(2,3-dihydroxy-6-(7-hydroxy-4-oxo-4H-chromen-2-yl)phenyl)-5,7-dihydroxy-3-(4-hydroxyphenyl)-4H-chromen-4-one). 12.03 mg, 9% yield, yellow solid. Semi-prep HPLC, injection volume was 250 μL. ACN/Water = 40: 60, flow rate 5.0 mL/min, λ = 300 nm, ^{1}H NMR (500 MHz, DMSO-*d6*) δ 13.08 (s, 1H), 10.77 (s, 2H), 10.08 (s, 1H), 8.37 (s, 1H), 7.75 (d, *J* = 8.7 Hz, 1H), 7.51 (d, *J* = 8.5 Hz, 2H), 7.17 (d, *J* = 8.3 Hz, 1H), 7.00 (d, *J* = 8.5 Hz, 2H), 6.95 (d, *J* = 8.3 Hz, 1H), 6.82 (dd, *J* = 8.7, 2.3 Hz, 1H), 6.52 (s, 1H), 6.49 (s, 1H), 5.99 (d, *J* = 2.3 Hz, 1H), 3.78 (s, 3H). ^{13}C NMR (126 MHz, DMSO) δ 180.3, 176.1, 165.0, 162.5, 162.3, 159.3, 159.2, 157.6, 156.7, 154.2, 147.9, 144.3, 130.3, 126.4, 124.5, 123.1, 122.1, 120.0, 119.6, 115.8, 114.8, 114.4, 113.8, 108.4, 108.2, 104.3, 102.0, 93.1, 55.2. HRMS(ESI-TOF) [**M**-**H**]$^{-}$ calculated for $C_{31}H_{19}O_{10}$ = 551.0984, found 551.0982.

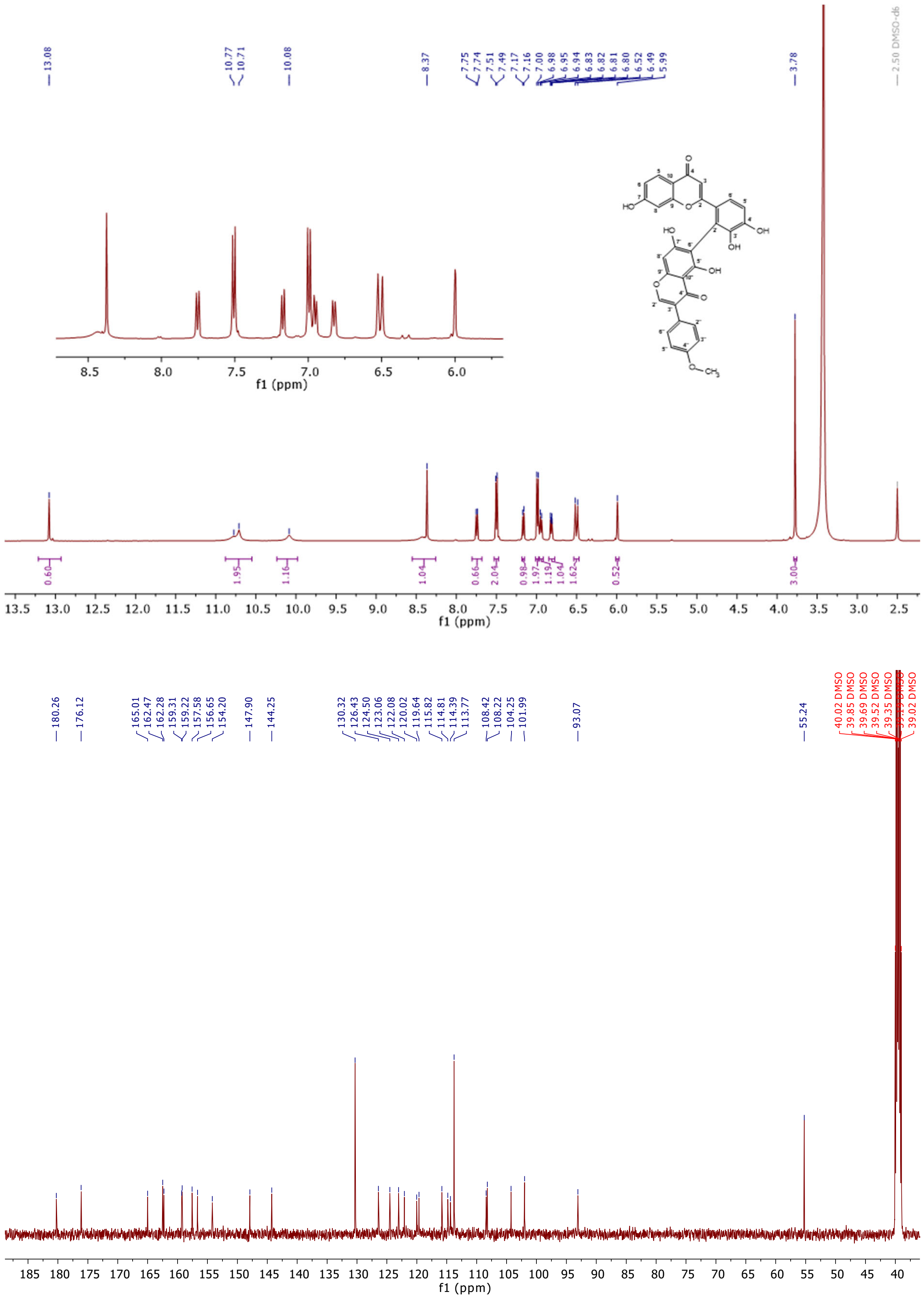

Figure S21.1. ^{1}H and ^{13}C NMR of biflavone 20

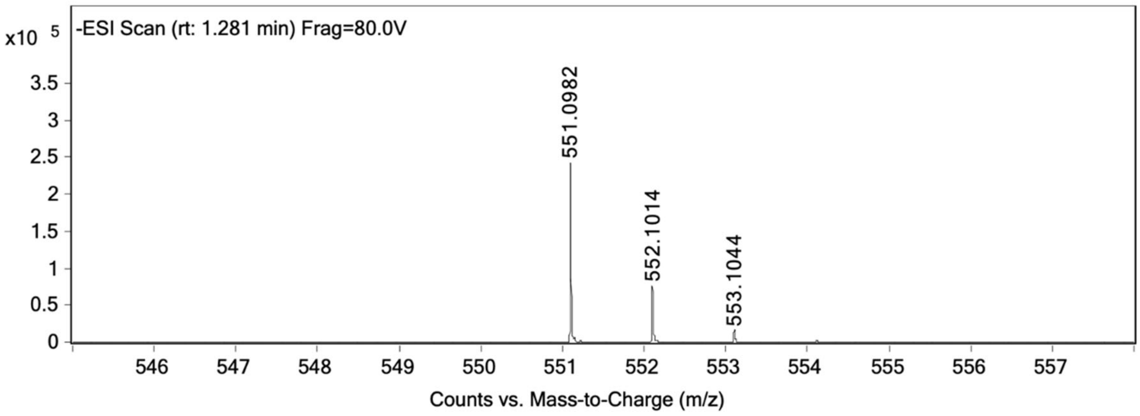

Figure S21.1. HRMS spectrum of biflavone 20

21 Wogonin + 3',4'-dihydroxyflavone (6-(2,3-dihydroxy-6-(4-oxo-4H-chromen-2-yl)phenyl)-5,7-dihydroxy-8-methoxy-2-phenyl-4H-chromen-4-one), 22.3 mg, 17% yield, yellow solid. Semi-prep HPLC, injection volume was 250 μL. ACN/Water = 48: 52, flow rate 5.0 mL/min, λ = 300 nm, ^{1}H NMR (500 MHz, DMSO-*d6*) δ 12.73 (s, 1H), 8.08 (d, *J* = 8.3 Hz, 2H), 7.92 (dd, *J* = 7.9, 1.6 Hz, 1H), 7.67 (t, *J* = 7.8 Hz, 1H), 7.61-7.59 (m, 3H), 7.37 (dd, *J* = 7.5 Hz, 1H), 7.25-7.21 (m, 2H), 7.02 (s, 1H), 6.99 (d, *J* = 8.4 Hz, 1H), 6.14 (s, 1H), 3.80 (s, 3H). ^{13}C NMR (126 MHz, DMSO) δ 182.5, 177.0, 166.1, 163.3, 156.1, 155.7, 154.6, 149.1, 148.6, 144.8, 134.4, 132.6, 131.2, 129.8, 127.9, 126.8, 125.7, 125.2, 124.7, 123.4, 120.6, 119.8, 118.2, 114.9, 109.1, 109.0, 105.7, 103.8, 62.0. HRMS(ESI-TOF) [**M**-**H**]$^{-}$ calculated for $C_{31}H_{19}O_9$ = 535.1035, found 535.1032.

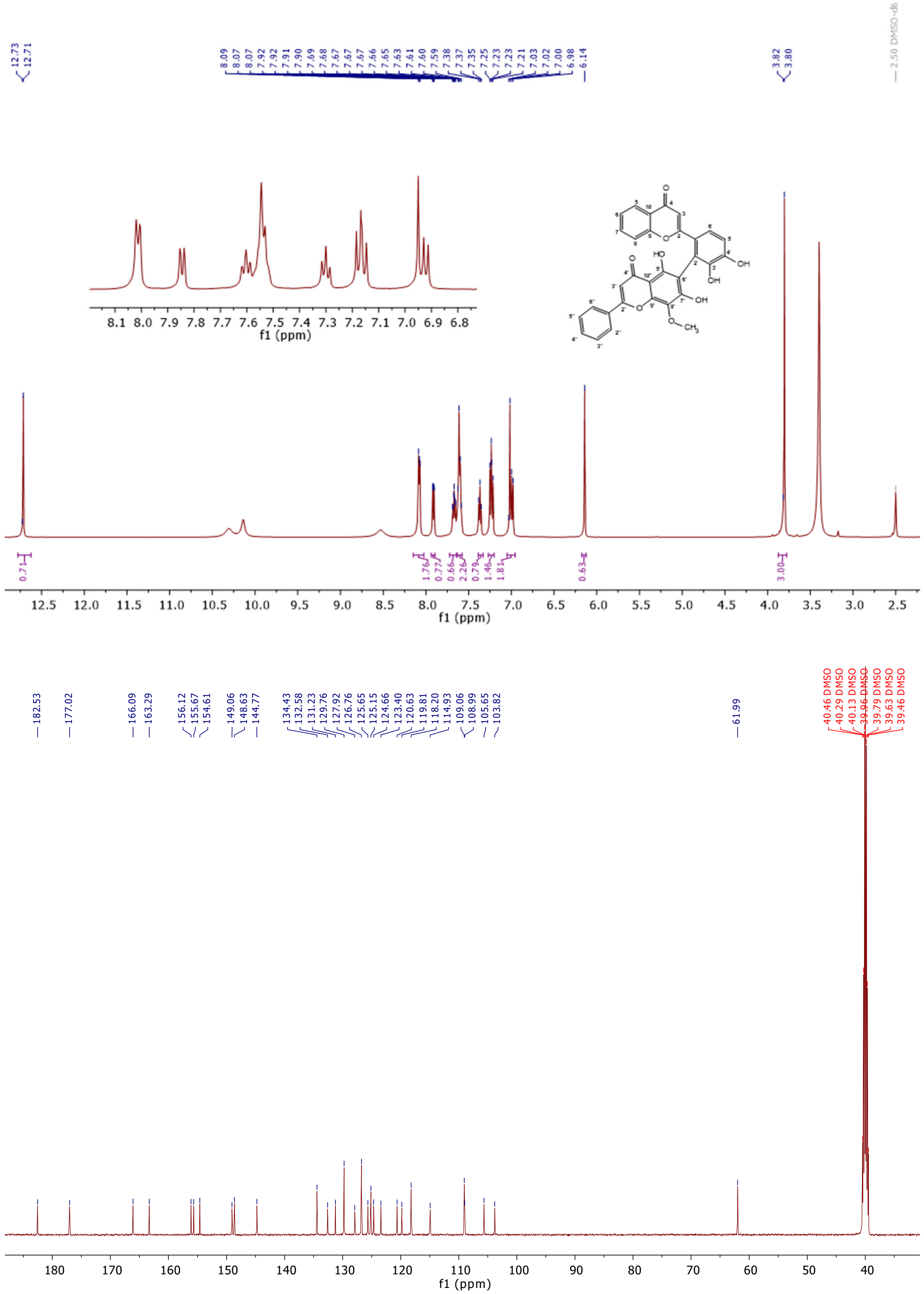

Figure S22.1. ^{1}H and ^{13}C NMR of biflavone 21

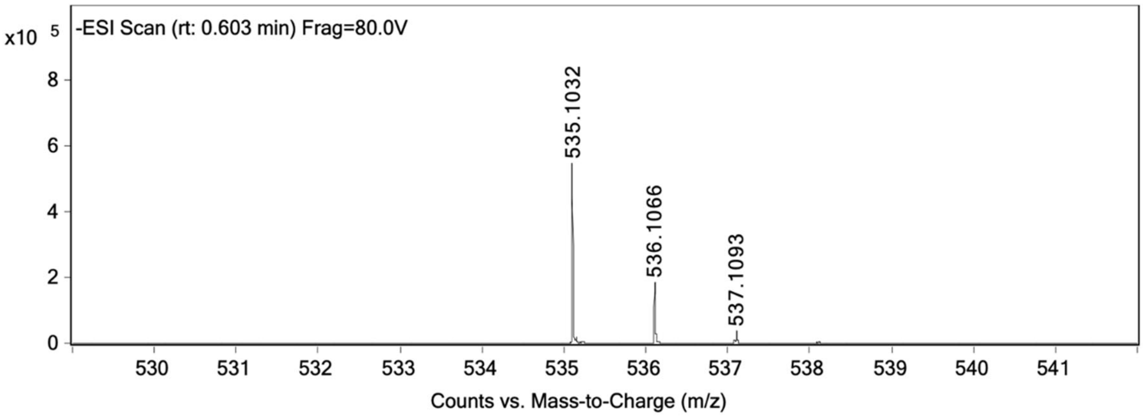

Figure S22.2. ^{1}H and ^{13}C NMR of biflavone 21

22 Wogonin + 5,3',4'-trihydroxyflavone (6-(2,3-dihydroxy-6-(5-hydroxy-4-oxo-4H-chromen-2-yl)phenyl)-5,7-dihydroxy-8-methoxy-2-phenyl-4H-chromen-4-one), 9.5 mg, 7% yield, yellow solid. Semi-prep HPLC, injection volume was 250 µL. ACN/Water = 40: 60, flow rate 5.0 mL/min, λ = 300 nm, ^{1}H NMR (500 MHz, DMSO-*d6*) δ 12.70 (s, 1H), 12.61 (s, 1H), 8.08 (d, *J* = 7.9 Hz, 2H,), 7.62 (m, 3H), 7.51 (t, *J* = 8.3 Hz, 1H), 7.26 (d, *J* = 8.4 Hz, 1H), 7.01 (s, 1H), 6.98 (d, *J* = 8.3 Hz, 1H), 6.68 (d, *J* = 8.2 Hz, 1H), 6.58 (d, *J* = 8.4 Hz, 1H), 6.20 (s, 1H), 3.81 (s, 3H). ^{13}C NMR (126 MHz, DMSO) δ 183.0, 182.5, 167.9, 163.3, 160.2, 156.3, 155.7, 154.5, 149.2, 149.1, 144.9, 136.2, 132.6, 131.2, 129.8, 128.0, 126.8, 123.9, 121.1, 119.9, 114.9, 111.1, 110.1, 108.9, 107.4, 107.1, 105.6, 103.8, 62.0. HRMS(ESI-TOF) [**M-H**]$^{-}$ calculated for $C_{31}H_{19}O_{10}$ = 551.0984, found 551.0980.

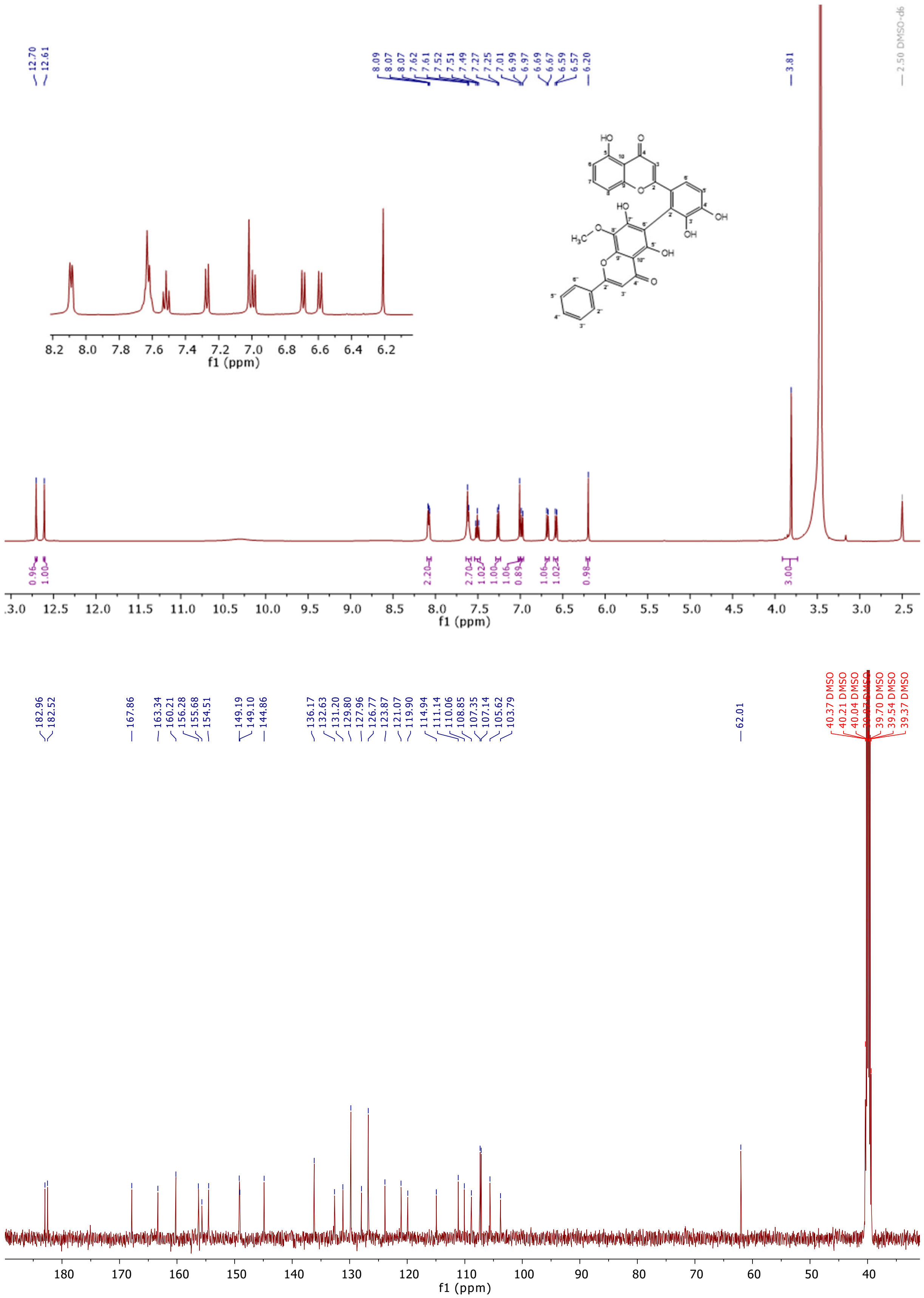

Figure S23.1. ^{1}H and ^{13}C NMR of biflavone 22

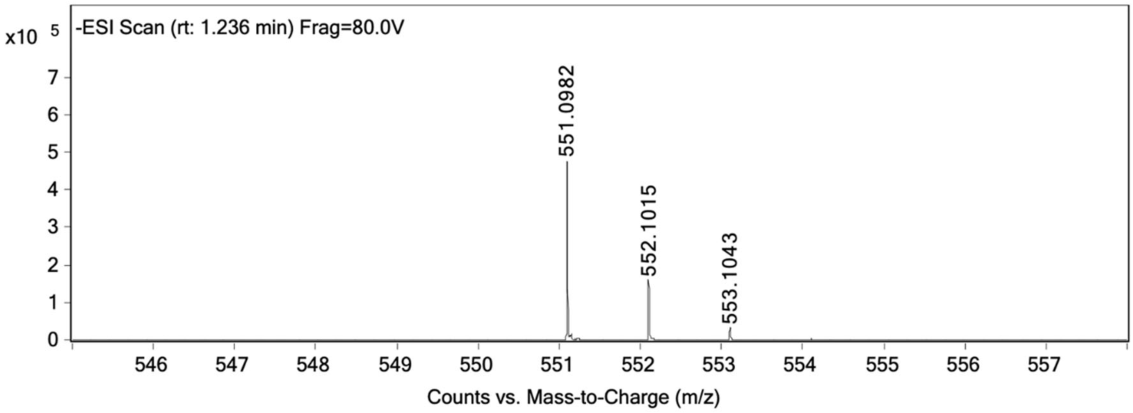

Figure S23.2. ^{1}H and ^{13}C NMR of biflavone 22

23 Wogonin + 6,3',4'-trihydroxyflavone (6-(2,3-dihydroxy-6-(6-hydroxy-4-oxo-4H-chromen-2-yl)phenyl)-5,7-dihydroxy-8-methoxy-2-phenyl-4H-chromen-4-one), 6.1 mg, 5% yield, yellow solid. Semi-prep HPLC, injection volume was 250 µL. ACN/Water = 40: 60, flow rate 5.0 mL/min, λ = 300 nm, ^{1}H NMR (500 MHz, DMSO-*d6*) δ 12.65 (s, 1H), 10.24 (s, 1H), 10.04 (s, 1H), 9.85 (s, 1H), 8.44 (s, 1H), 8.07 (dd, *J* = 8.2, 1.7 Hz, 2H), 7.60 (m, 3H), 7.19 – 7.13 (m, 2H), 7.07 (m, 2H), 7.00 (s, 1H), 6.93 (d, *J* = 8.4 Hz, 1H), 6.01 (s, 1H), 3.78 (s, 3H). ^{13}C NMR (126 MHz, DMSO) δ 182.5, 176.9, 165.6, 163.3, 155.7, 155.0, 154.6, 149.8, 149.0, 148.4, 144.7, 132.6, 131.3, 129.8, 127.9, 126.8, 124.9, 124.3, 123.2, 120.4, 119.7, 119.5, 114.9, 109.0, 108.1, 107.8, 105.7, 103.8, 62.0. HRMS(ESI-TOF) [**M**-**H**]$^{-}$ calculated for $C_{31}H_{19}O_{10}$ = 551.0984, found 551.0982.

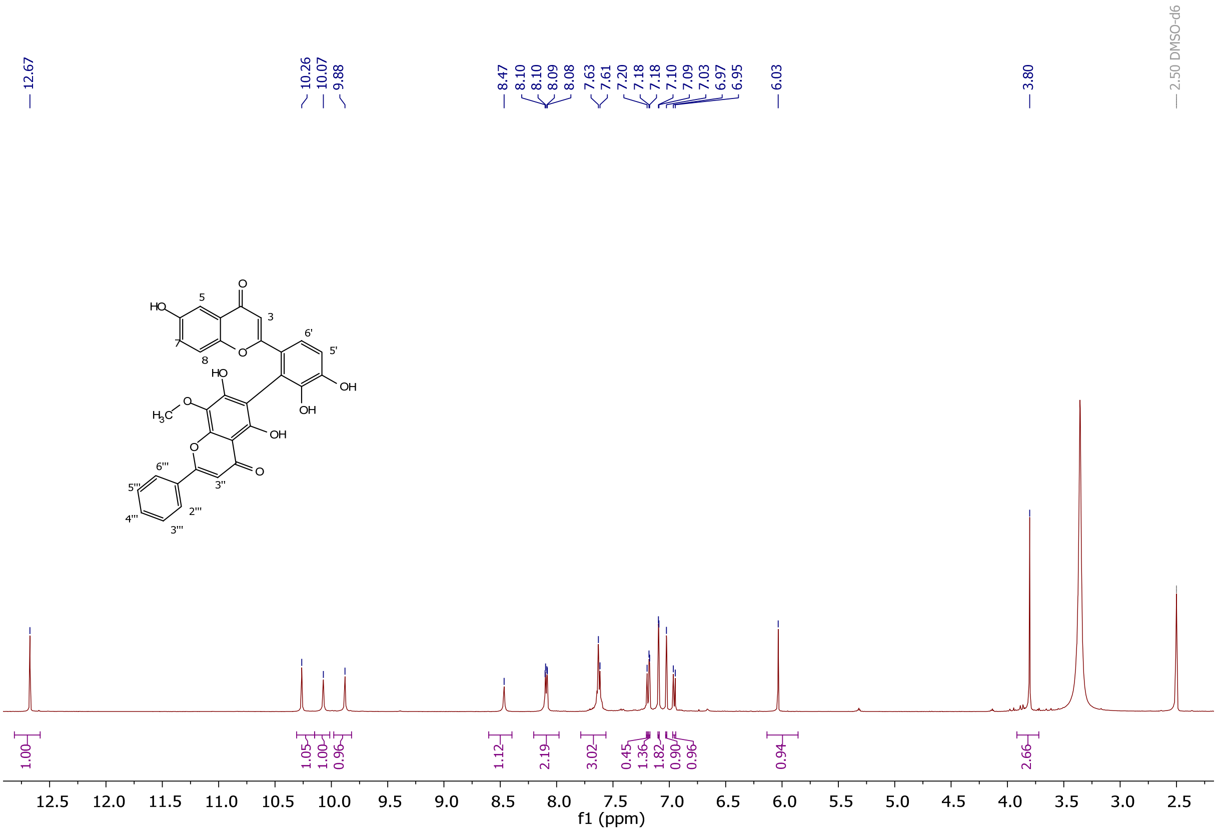

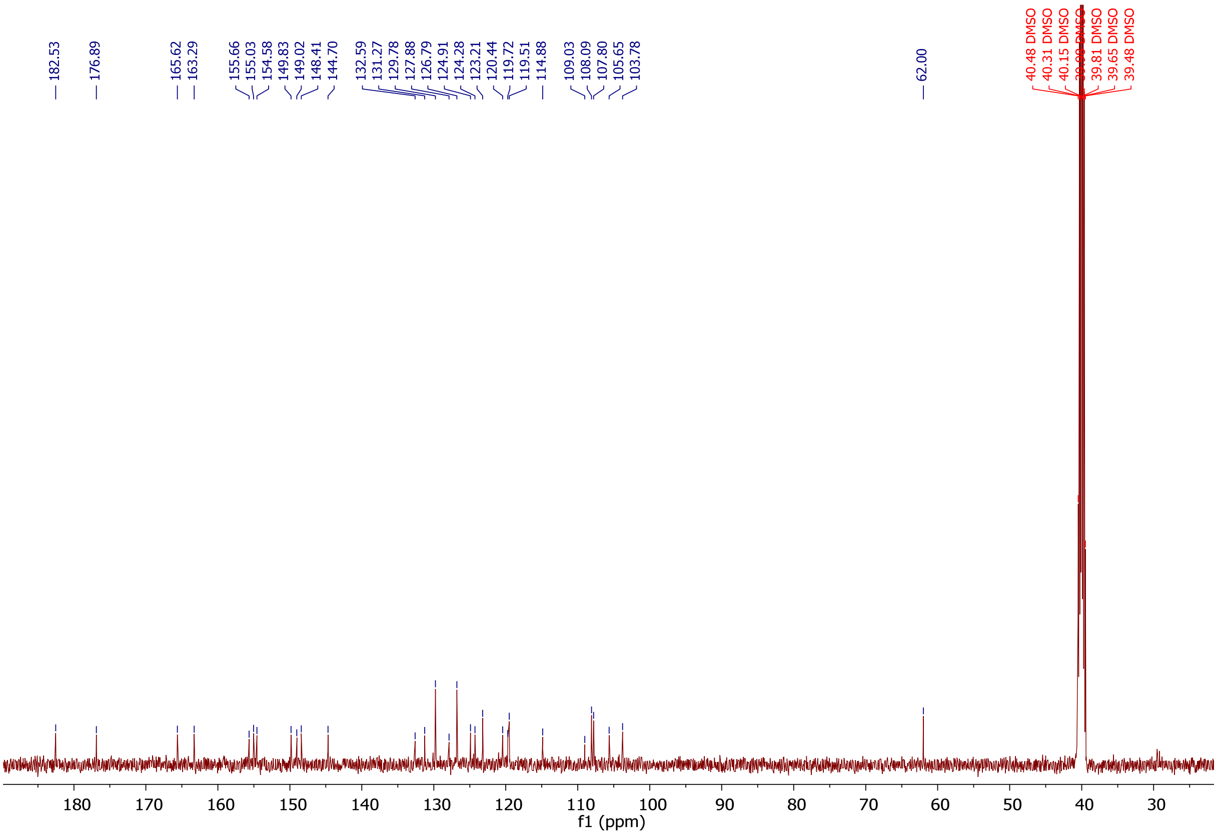

Figure S24.1. ^{1}H and ^{13}C NMR of biflavone 23

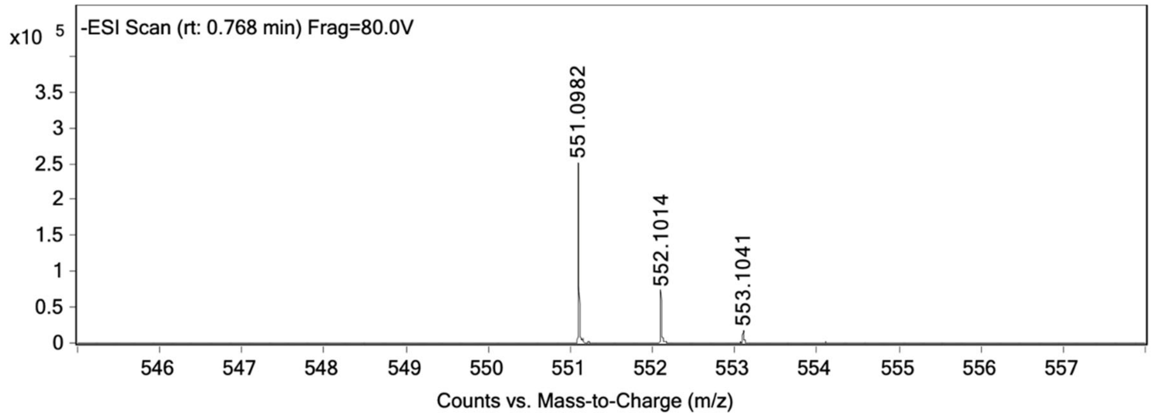

Figure S24.1. ^{1}H and ^{13}C NMR of biflavone 23

24 Wogonin + 7,3',4'-trihydroxyflavone (6-(2,3-dihydroxy-6-(7-hydroxy-4-oxo-4H-chromen-2-yl)phenyl)-5,7-dihydroxy-8-methoxy-2-phenyl-4H-chromen-4-one), 13.2 mg, 10% yield, yellow solid. Semi-prep HPLC, injection volume was 250 µL. ACN/Water = 42: 58, flow rate 5.0 mL/min, λ = 300 nm, ^{1}H NMR (500 MHz, DMSO-*d6*) δ 12.70 (s, 1H), 10.64 (s, 1H), 10.25 (s, 1H), 10.07 (s, 1H), 8.46 (s, 1H), 8.09 (dd, *J* = 7.8, 1.9 Hz, 2H), 7.74 (d, *J* = 8.6 Hz, 1H), 7.62 – 7.60 (m, 3H), 7.18 (d, *J* = 8.4 Hz), 7.03 (s, 1H), 6.96 (d, *J* = 8.3 Hz, 1H), 6.78 (dd, *J* = 8.7, 2.3 Hz), 6.43 (d, *J* = 2.2 Hz, 1H), 6.02 (s, 1H), 3.79 (s, 3H). ^{13}C NMR (126 MHz, DMSO) δ 182.6, 176.5, 165.4, 163.3, 162.8, 157.9, 155.6, 154.6, 149.0, 148.4, 144.7, 132.6, 131.3, 129.8, 127.9, 126.8, 126.8, 124.8, 120.4, 119.7, 116.2, 115.2, 114.9, 109.1, 108.7, 105.7, 103.8, 102.3, 61.9. HRMS(ESI-TOF) **[M-H]**$^{-}$ calculated for $C_{31}H_{19}O_{10}$ = 551.0984, found 551.0982.

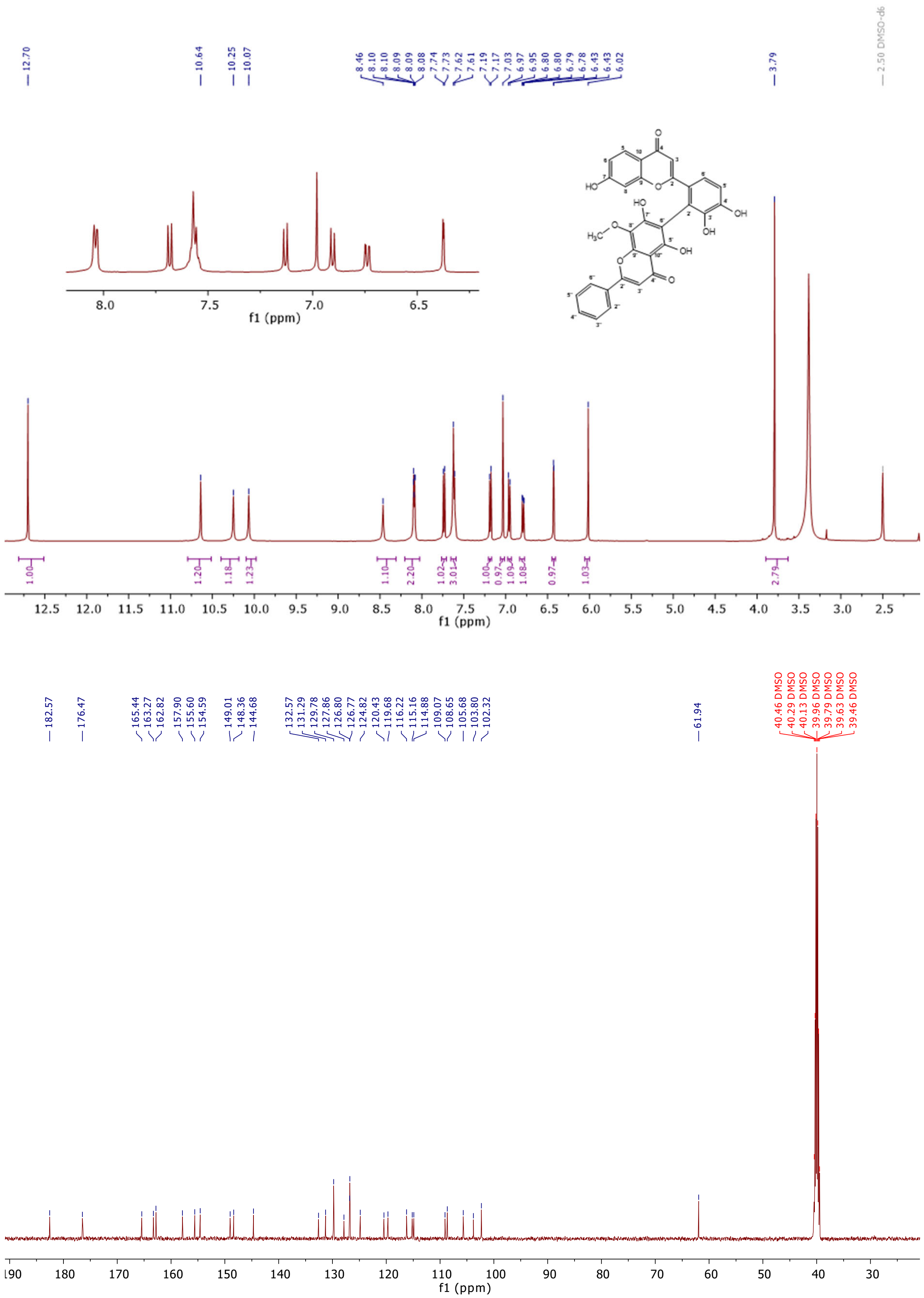

Figure S25.1. ^{1}H and ^{13}C NMR of biflavone 24

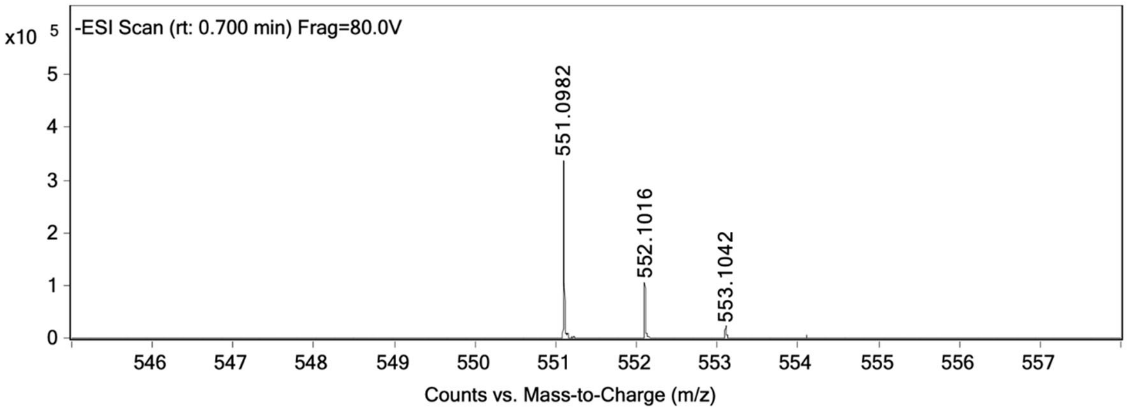

Figure S25.1. HRMS spectrum of biflavone 24

25 Chrysoeriol + 3',4'-dihydroxyflavone (6-(2,3-dihydroxy-6-(4-oxo-4H-chromen-2-yl)phenyl)-5,7-dihydroxy-2-(4-hydroxy-3-methoxyphenyl)-4H-chromen-4-one), 15.4 mg, 11% yield, yellow solid. Semi-prep HPLC, injection volume was 250 μL. ACN/Water = 21.5: 71.5, flow rate 5.0 mL/min, λ = 300 nm, ^{1}H NMR (500 MHz, DMSO-*d6*) δ 13.14 (s, 1H), 7.91 (dd, *J* = 7.9, 1.6 Hz, 1H), 7.68 (dd, *J* = 7.7 Hz, 1H), 7.60 – 7.55 (m, 2H), 7.38 (dd, *J* = 7.5 Hz, 1H), 7.27 (d, *J* = 8.4 Hz, 1H), 7.22 (d, *J* = 8.3 Hz, 1H), 6.97 (d, *J* = 8.6 Hz, 1H), 6.94 (d, *J* = 8.4 Hz, 1H), 6.91 (s, 1H), 6.58 (s, 1H), 6.12 (s, 1H), 3.89 (s, 3H)^{13}C NMR (126 MHz, DMSO) δ 182.3, 177.0, 166.2, 164.0, 162.5, 159.3, 156.8, 156.2, 151.2, 148.6, 148.5, 144.8, 134.4, 125.6, 125.1, 124.8, 123.4, 122.0, 120.9, 120.6, 120.3, 118.2, 116.3, 114.8, 110.7, 109.0, 108.6, 103.9, 103.7, 93.9, 63.6, 56.4. HRMS(ESI-TOF) [**M-H**]$^-$ calculated for $C_{31}H_{19}O_{10}$ = 551.0984, found 551.0982.

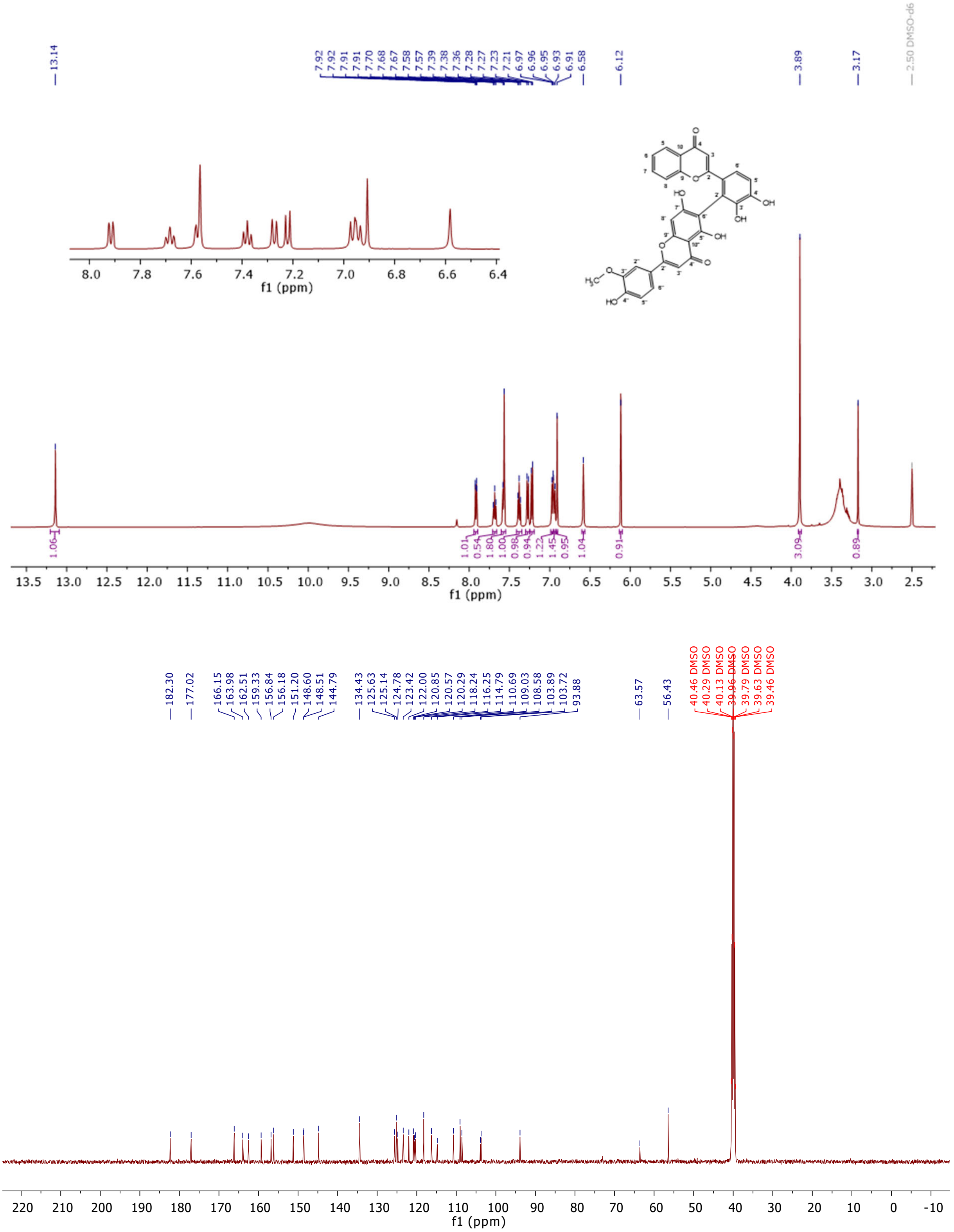

Figure S26.1. ^{1}H and ^{13}C NMR of biflavone 25

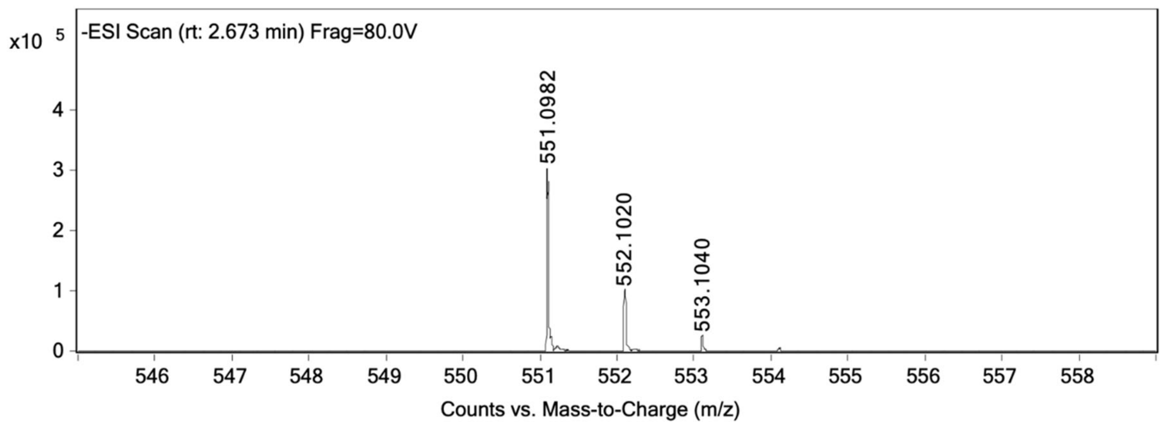

Figure S26.2. HRMS spectrum of biflavone 25

26 Chrysoeriol + 5,3',4'-dihydroxyflavone (6-(2,3-dihydroxy-6-(5-hydroxy-4-oxo-4H-chromen-2-yl)phenyl)-5,7-dihydroxy-2-(4-hydroxy-3-methoxyphenyl)-4H-chromen-4-one), 11.8 mg, 8 % yield, yellow solid. Semi-prep HPLC, injection volume was 250 µL. ACN/Water = 35: 65, flow rate 5.0 mL/min, λ = 300 nm, ^{1}H NMR (500 MHz, DMSO-*d6*) δ 13.16 (s, 1H), 12.63 (s, 1H), 7.61 – 7.56 (m, 2H), 7.53 (dd, *J* = 8.3 Hz, 1H), 7.25 (d, *J* = 8.3 Hz, 1H), 6.97 (d, *J* = 8.2 Hz, 1H), 6.95 (d, *J* = 8.1 Hz, 1H), 6.92 (s, 1H), 6.70 (d, *J* = 8.3 Hz, 1H), 6.65 (d, *J* = 8.4 Hz, 1H), 6.59 (s, 1H), 6.18 (s, 1H), 3.90 (s, 3H). ^{13}C NMR (126 MHz, DMSO) δ = 182.9, 182.3, 167.9, 164.0, 162.4, 160.3, 159.3, 156.9, 156.4, 151.2, 149.1, 148.5, 144.9, 136.2, 124.0, 122.0, 121.0, 120.9, 120.4, 116.3, 114.8, 111.1, 110.7, 110.1, 108.4, 107.4, 107.2, 103.9, 103.7, 93.9, 56.4. HRMS(ESI-TOF) [**M-H**]$^-$ calculated for $C_{31}H_{19}O_{11}$ = 567.0933, found 567.0934.

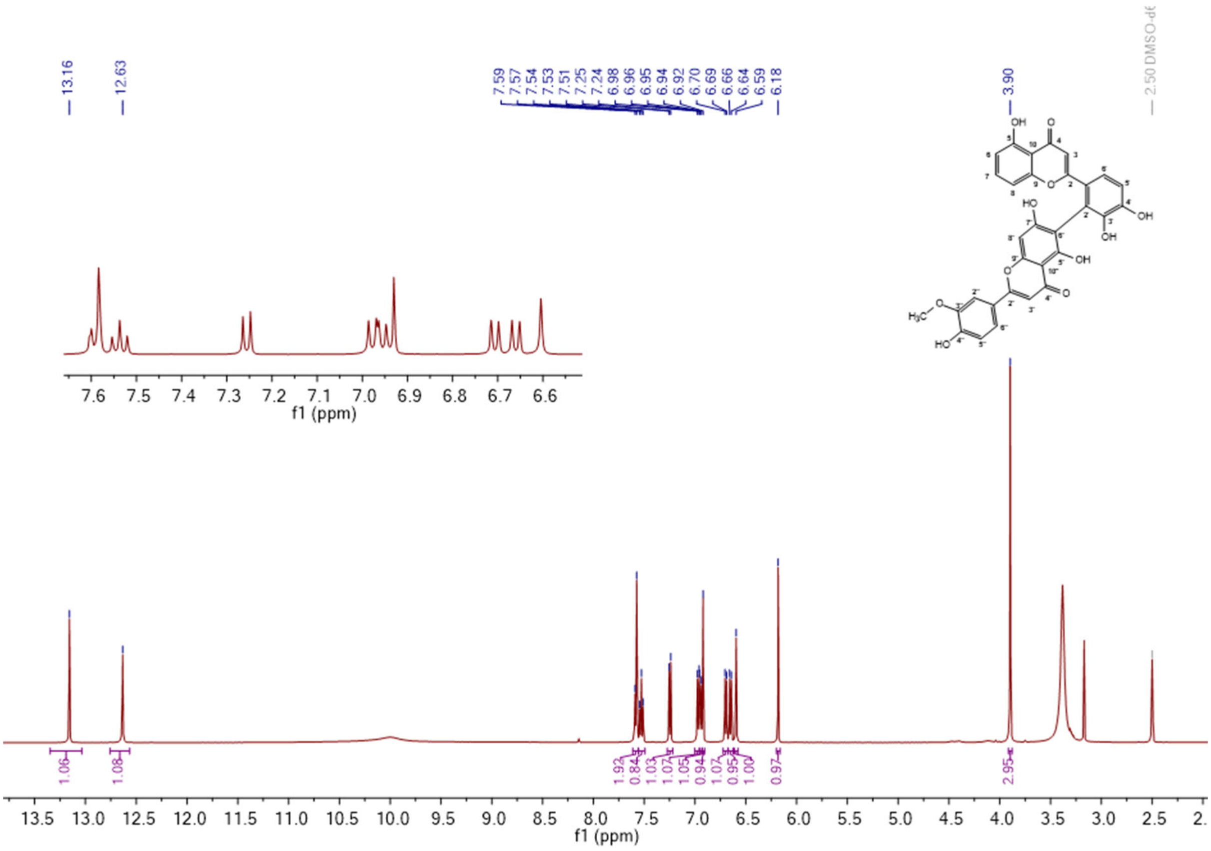

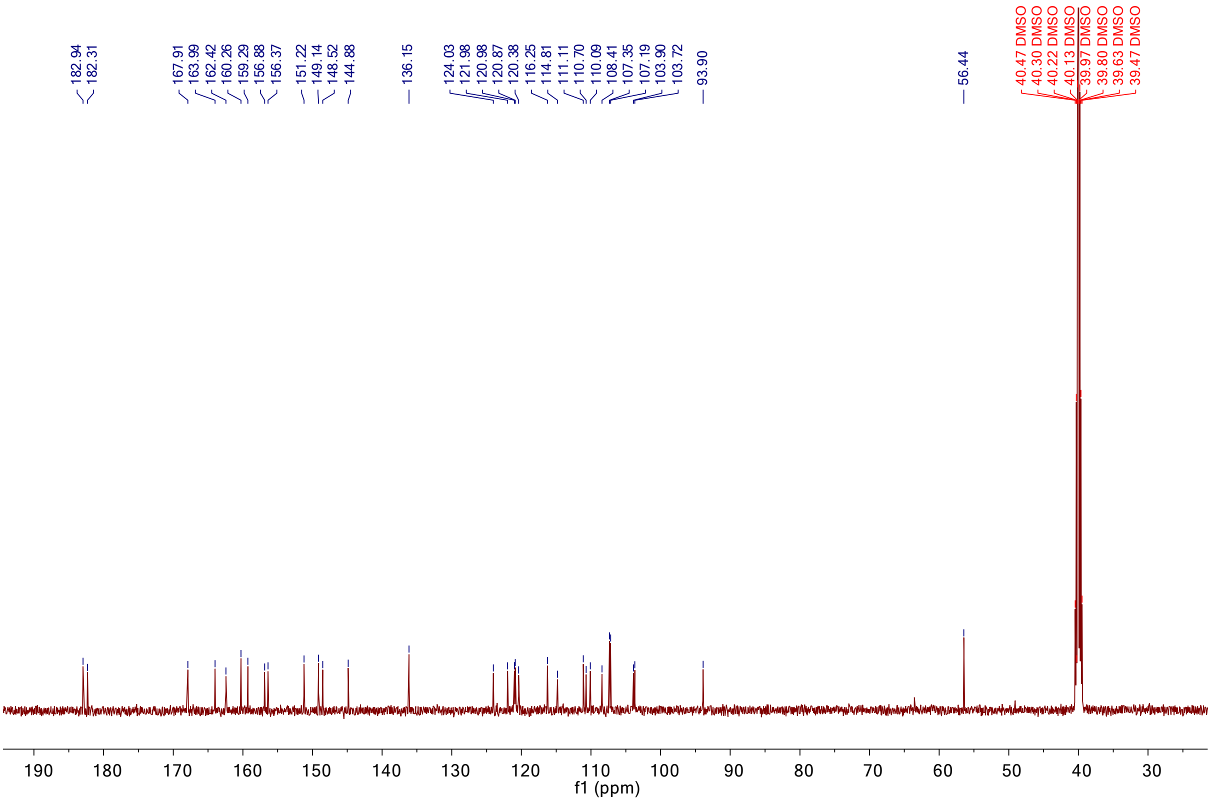

Figure S27.1. ^{1}H and ^{13}C NMR of biflavone 26

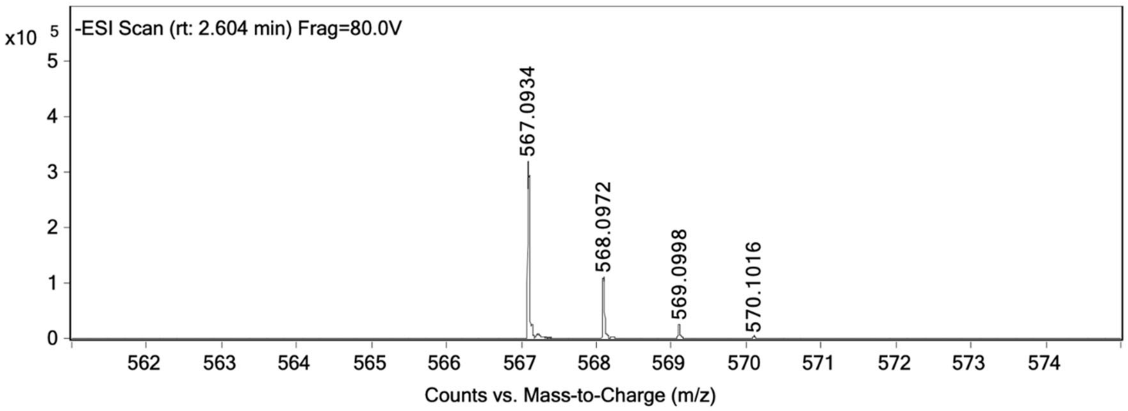

Figure S27.2.HRMS spectrum of biflavone 26

27 Chrysoeriol + 6,3',4'-dihydroxyflavone (6-(2,3-dihydroxy-6-(6-hydroxy-4-oxo-4H-chromen-2-yl)phenyl)-5,7-dihydroxy-2-(4-hydroxy-3-methoxyphenyl)-4H-chromen-4-one), 21.6 mg, 15% yield, yellow solid. Semi-prep HPLC, injection volume was 250 μL. ACN/Water = 21.5: 71.5, flow rate 5.0 mL/min, λ = 300 nm, ^{1}H NMR (500 MHz, DMSO-d_6) δ 13.12 (s, 1H), 7.61 – 7.55 (m, 2H), 7.20 – 7.16 (m, 2H), 7.15 (s, 1H), 7.11 (dd, J = 9.0, 2.9 Hz, 1H), 6.96 (d, J = 8.3 Hz, 1H), 6.94 (d, J = 8.3 Hz, 1H), 6.91 (s, 1H), 6.58 (s, 1H), 6.03 (s, 1H), 3.90 (s, 3H).^{13}C NMR (126 MHz, DMSO) δ = 182.9, 182.3, 167.9, 164.0, 162.4, 160.3, 159.3, 156.9, 156.4, 151.2, 149.1, 148.5, 144.9, 136.2, 124.0, 122.0, 121.0, 120.9, 120.4, 116.3, 114.8, 111.1, 110.7, 110.1, 108.4, 107.4, 107.2, 103.9, 103.7, 93.9, 56.4 HRMS(ESI-TOF) [**M**-**H**]$^-$ calculated for $C_{31}H_{19}O_{11}$ = 567.0933, found 567.0933

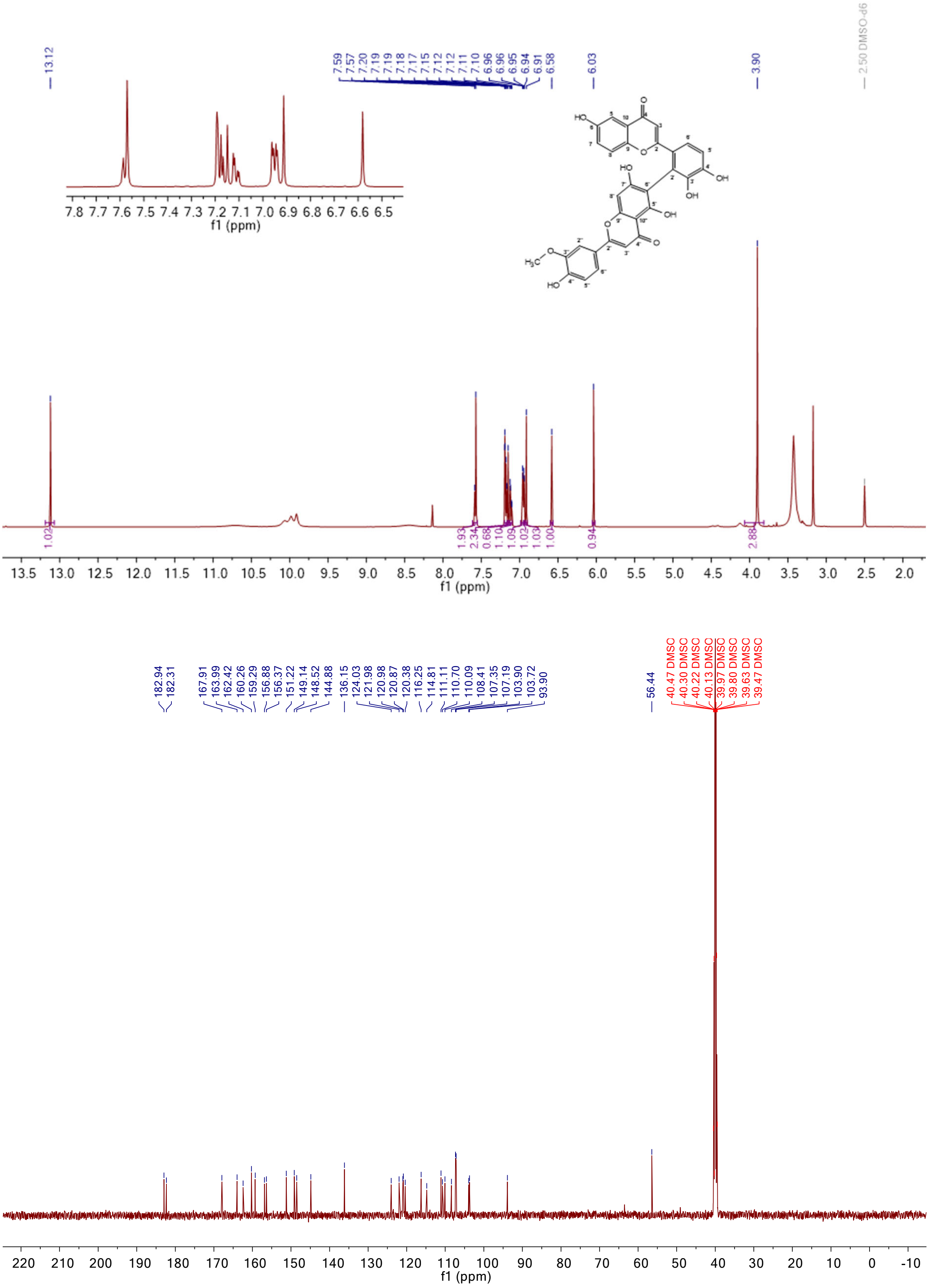

Figure S28.1. ^{1}H and ^{13}C NMR of biflavone 27

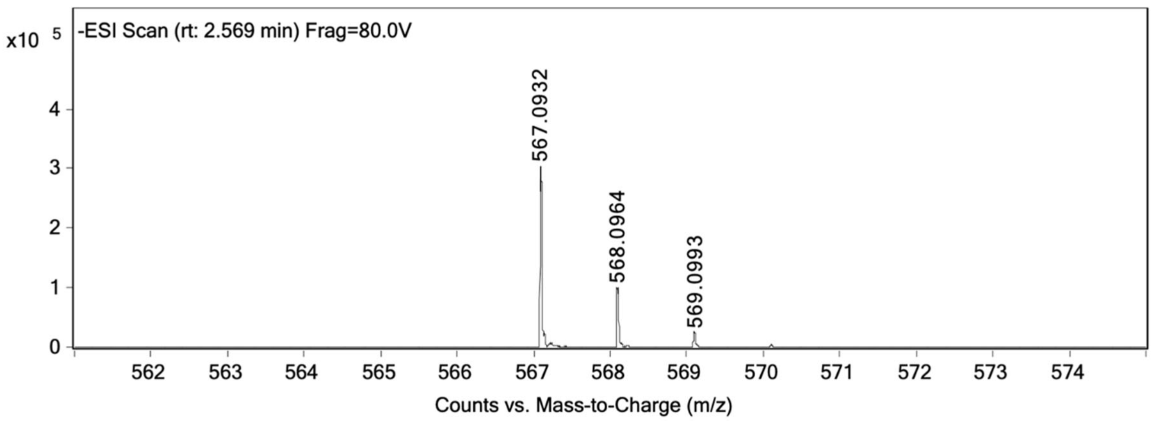

Figure S28.2. HRMS spectrum of biflavone 27

28 Chrysoeriol + 7,3',4'-dihydroxyflavone (6-(2,3-dihydroxy-6-(7-hydroxy-4-oxo-4H-chromen-2-yl)phenyl)-5,7-dihydroxy-2-(4-hydroxy-3-methoxyphenyl)-4H-chromen-4-one), 12.4 mg, 9 % yield, yellow solid. Semi-prep HPLC, injection volume was 250 µL. ACN/Water = 21.5: 71.5, flow rate 5.0 mL/min, λ = 300 nm, ^{1}H NMR (500 MHz, DMSO-d_6) δ 13.12 (s, 1H), 7.74 (d, *J* = 8.7 Hz, 1H), 7.62 – 7.55 (m, 2H), 7.17 (d, *J* = 8.4 Hz, 1H), 6.99 – 6.92 (m, 2H), 6.91 (s, 1H), 6.80 (dd, *J* = 8.7, 2.3 Hz, 1H), 6.57 (s, 1H), 6.49 (d, *J* = 2.2 Hz, 1H), 5.99 (s, 1H), 3.90 (s, 3H).^{13}C NMR (126 MHz, DMSO) δ = 182.3, 176.5, 165.5, 164.0, 162.8, 162.3, 159.3, 158.0, 156.8, 151.2, 148.5, 148.3, 144.7, 126.8, 125.0, 122.0, 120.9, 120.4, 120.1, 116.2, 115.2, 114.8, 110.7, 108.6, 103.9, 103.8, 102.4, 93.8, 73.0, 63.6, 56.4. HRMS(ESI-TOF) calculated [**M**-**H**]$^-$ for $C_{31}H_{19}O_{11}$ = 567.0933, found 567.0932.

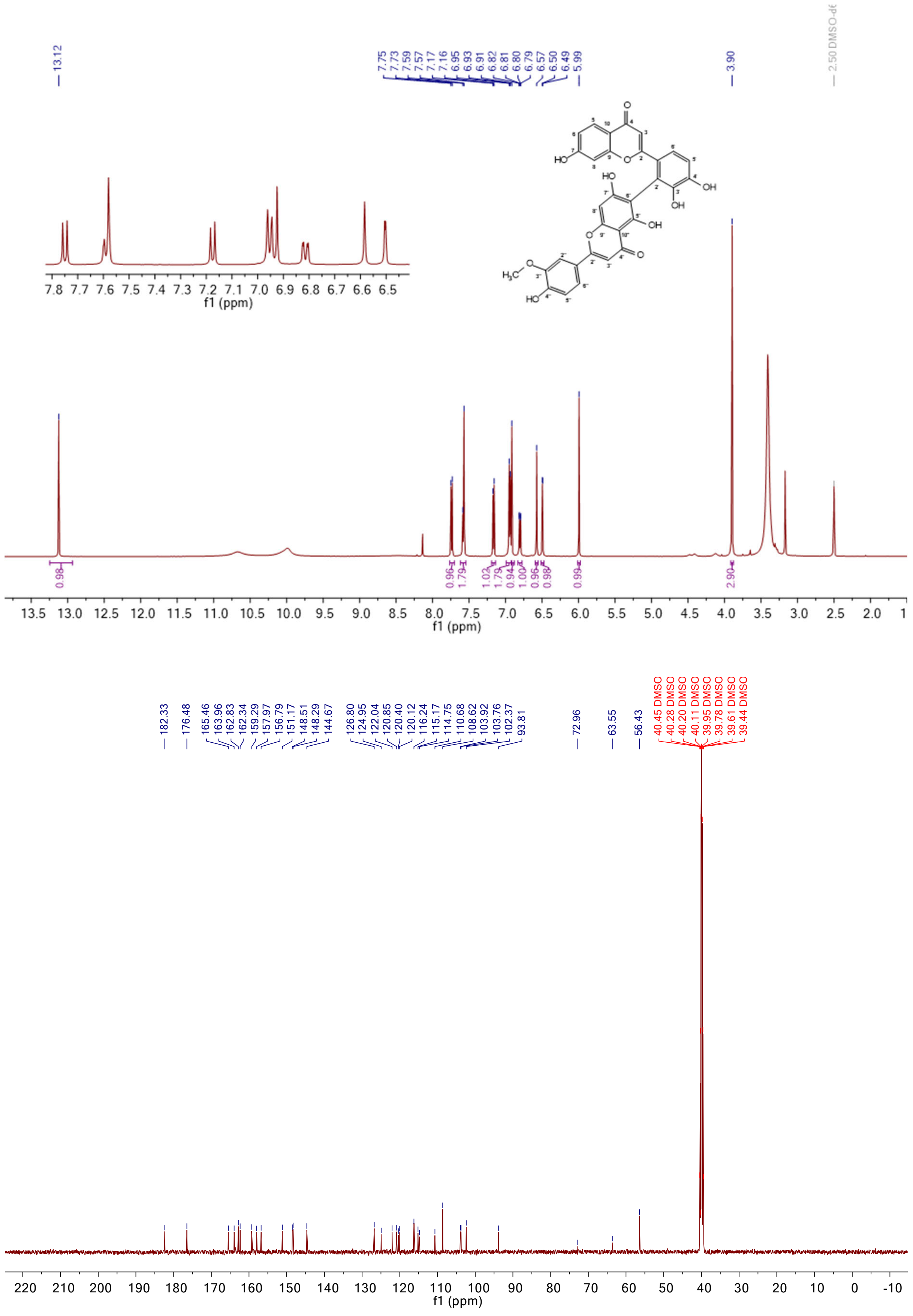

Figure S29.1. ^{1}H and ^{13}C NMR of biflavone 28

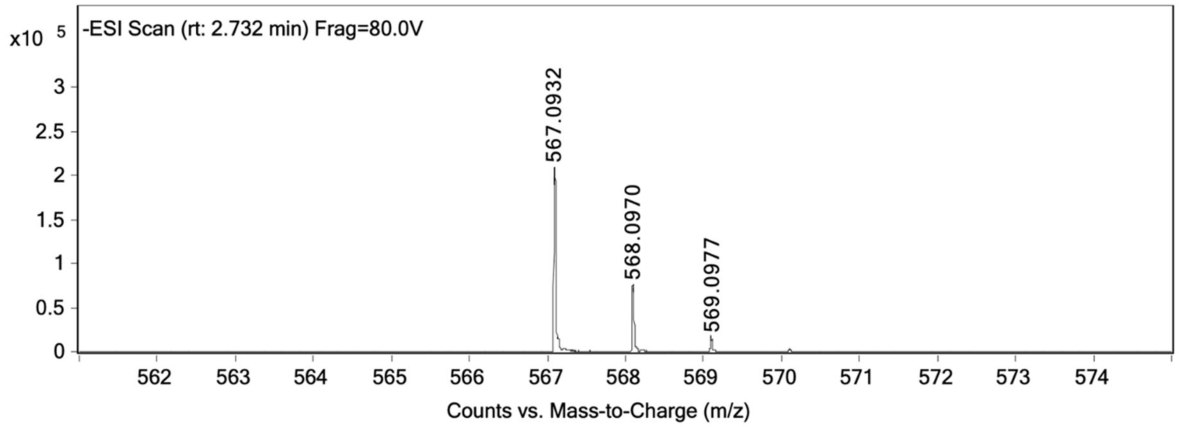

Figure S29.2. ^{1}H and ^{13}C NMR of biflavone 28

5a chrysin + 3',4'-dihydroxyflavone 8-(2,3-dihydroxy-6-(4-oxo-4H-chromen-2-yl)phenyl)-5,7-dihydroxy-2-phenyl-4H-chromen-4-one, 3 mg, 2 % yield, yellow solid. Semi-prep HPLC, injection volume was 250 µL. ACN/Water = 40: 60, flow rate 5.0 mL/min, λ = 300 nm, ^{1}H NMR (500 MHz, DMSO-d_6) δ 12.84 (s, 1H), 7.88 (d, *J* = 7.8 Hz, 1H), 7.69 (d, *J* = 7.8 Hz, 2H), 7.65 (t, *J* = 7.9 Hz, 1H), 7.53 (t, *J* = 7.3 Hz, 1H), 7.45 (t, *J* = 7.6 Hz, 2H), 7.36 (t, *J* = 7.5 Hz, 1H), 7.29 (d, *J* = 8.2 Hz, 1H), 7.05 (d, *J* = 8.3 Hz, 1H), 7.01 (d, *J* = 8.4 Hz, 1H), 6.94 (s, 1H), 6.30 (s, 1H), 6.09 (s, 1H). ^{13}C NMR (126 MHz, DMSO) δ = 182.6, 177.0, 166.3, 163.5, 162.6, 160.9, 156.0, 154.9, 148.8, 145.0, 134.5, 132.5, 131.2, 129.5, 126.5, 125.7, 125.2, 124.9, 123.3, 121.0, 119.1, 117.9, 115.2, 109.1, 105.2, 104.3, 103.9, 99.0, 56.5. HRMS(ESI-TOF) [**M-H**]$^-$ calculated for $C_{30}H_{17}O_8$ = 505.0929, found 505.0930.

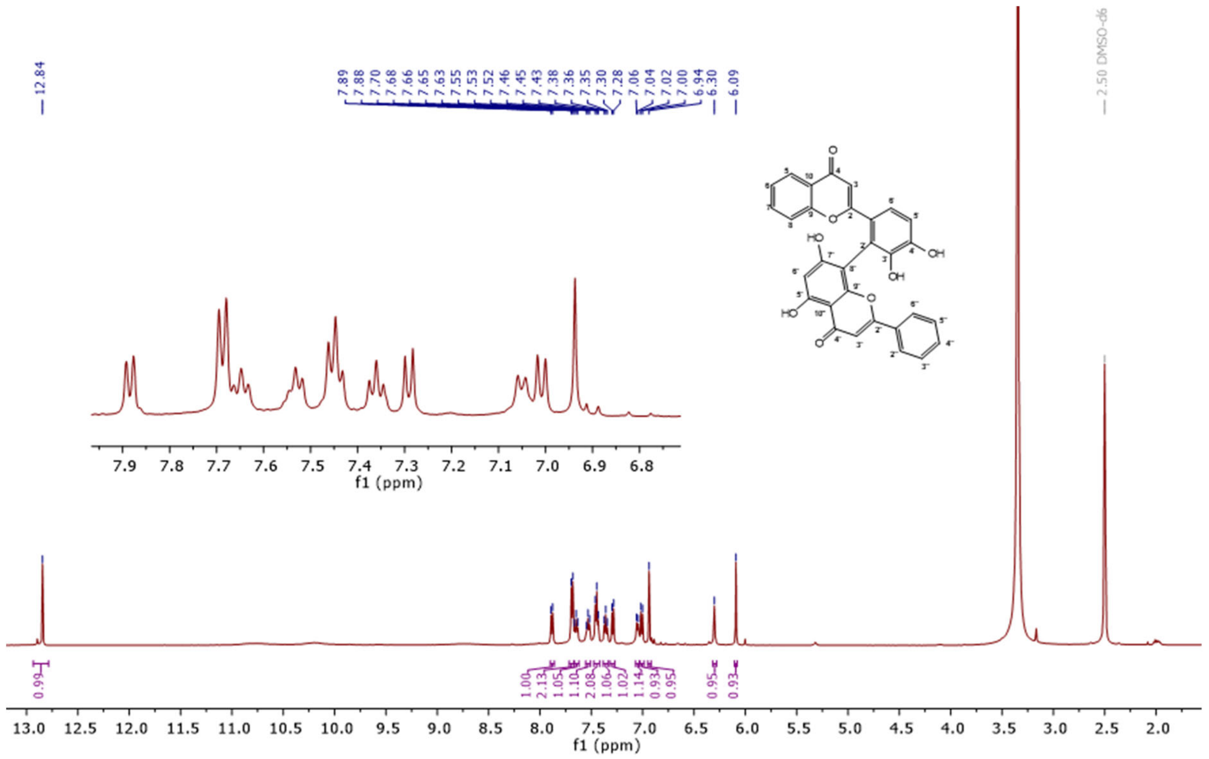

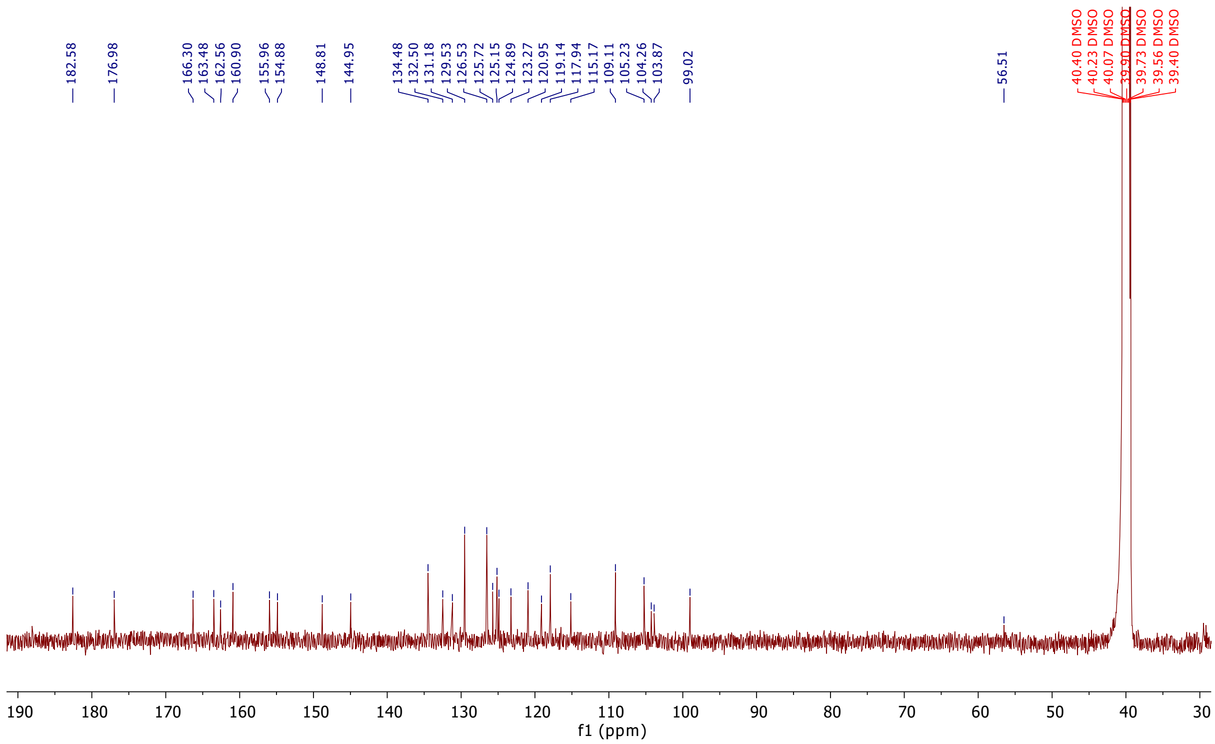

Figure S30.1. ^{1}H and ^{13}C NMR of isomer of biflavone 5a

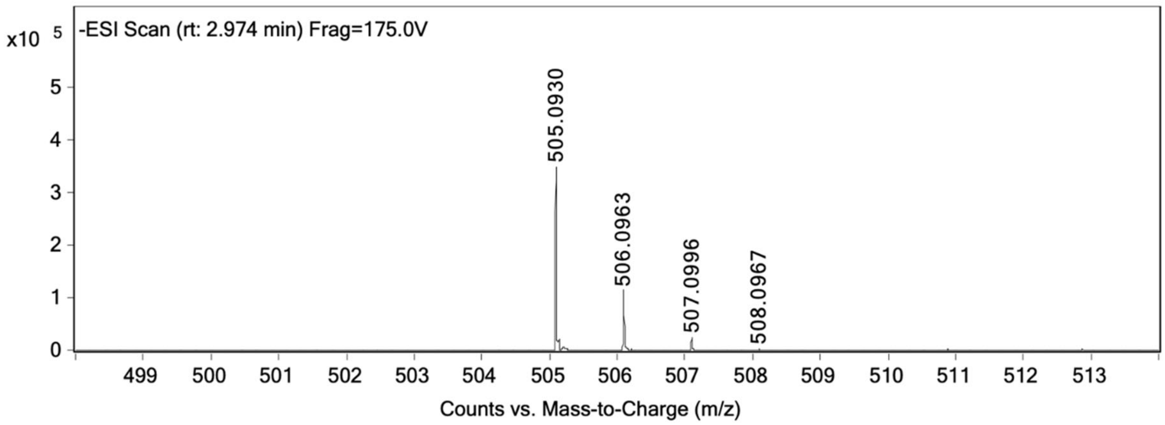

Figure S30.2. HRMS spectrum of isomer of biflavone 5a

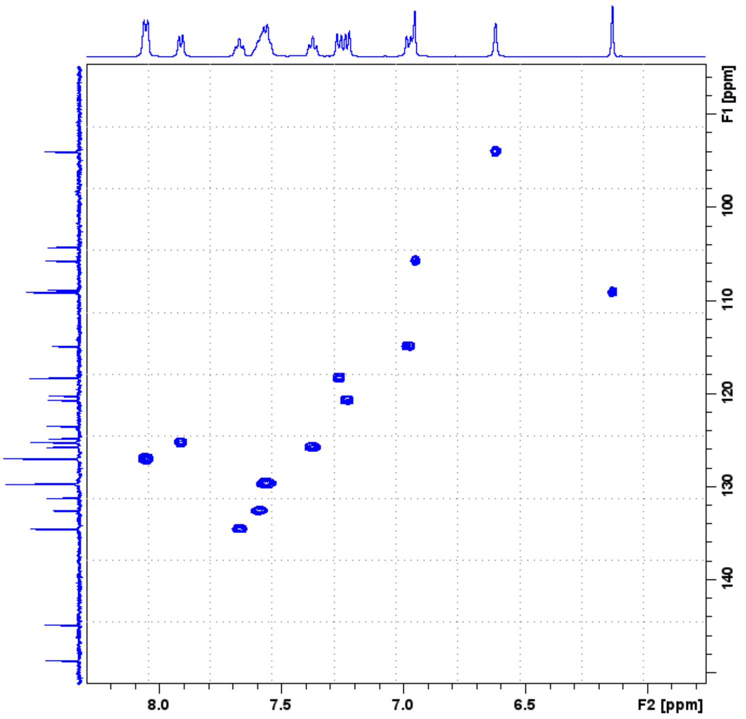

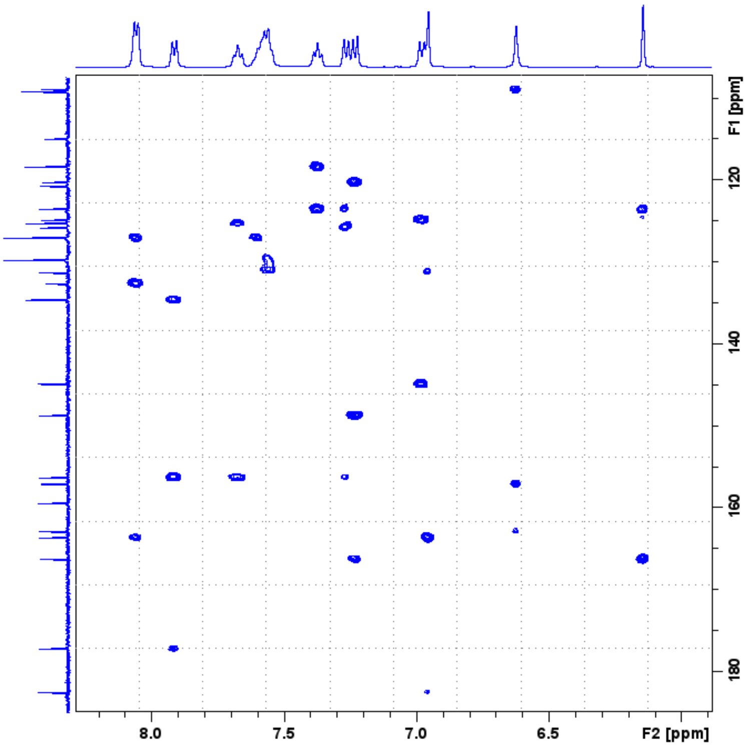

Figure S31 HSQC and HMBC of biflavone **5**

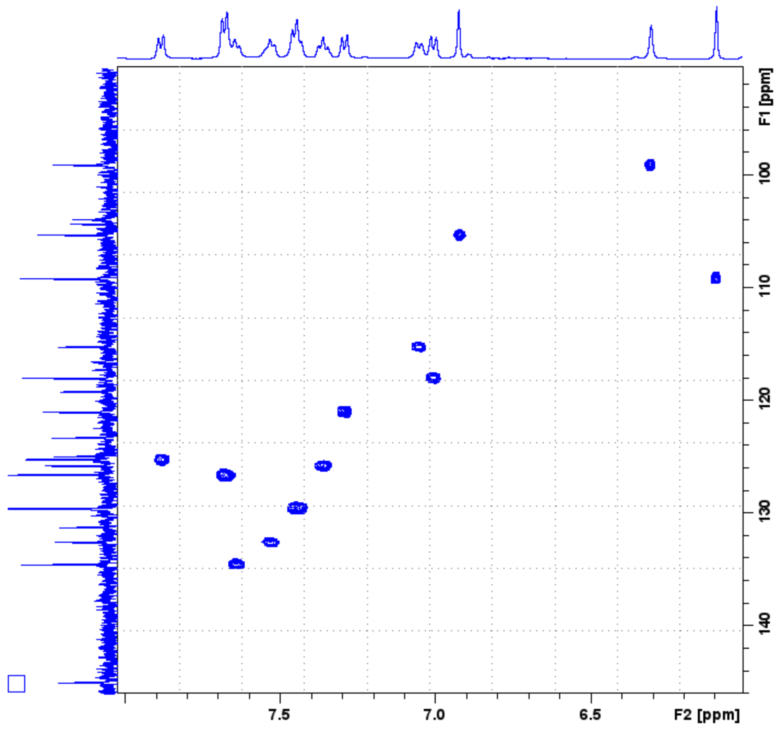

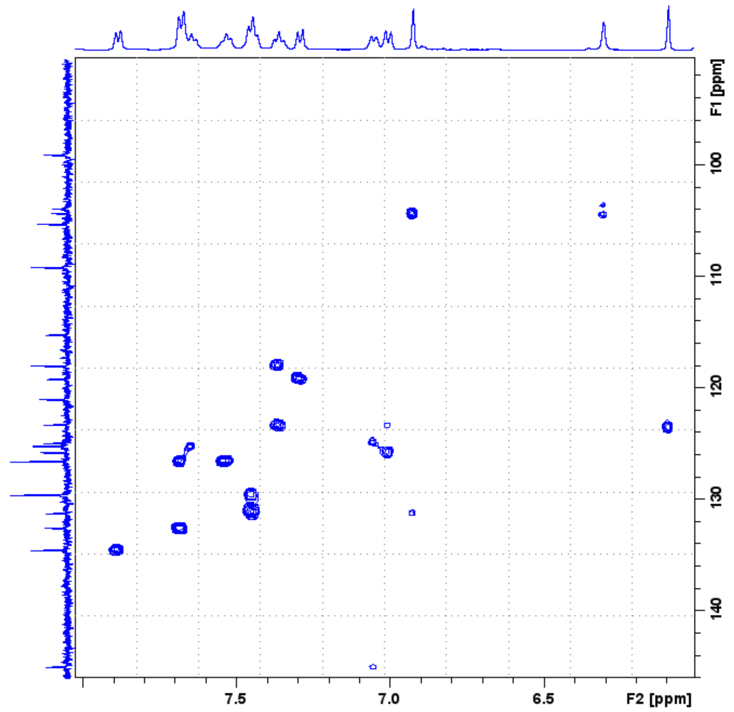

Figure S32.HSQC and HMBC of biflavone **5a**

(a)

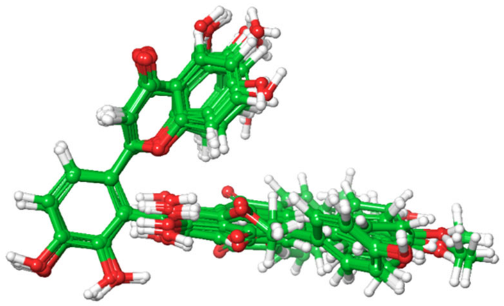

(b)

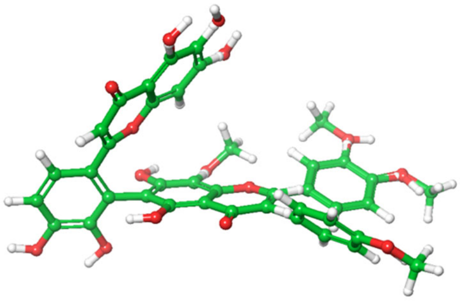

Figure S33. Alignment of the pharmacophoric features of flavonoid dimers. (a) Before alignment and (b) after alignment

Table S1. The ^{1}H chemical shifts of biflavonoids **1-28.**

δ of ^{1}H (*J*, Hz)							
Position	**1**	**5**	**9**	**13**	**17**	**21**	**25**
3	6.12 (s)	6.14 (s)	6.12 (s)	6.10 (s)	6.12 (s)	6.07 (s)	6.12 (s)
5	7.91 (d,7.4)	7.91 (d, 7.9)	7.91 (d, 7.8)	7.92 (d, 7.8)	7.92 (d, 7.8)	7.85 (dd, 7.9, 1.6)	7.91 (dd, 7.9, 1.6)
6	7.38 (dd, 8.0)	7.37 (dd, 7.5)	7.38 (dd, 7.5)	7.39 (dd, 7.5)	7.40 (dd, 7.5)	7.30 (dd, 7.5)	7.38 (dd, 7.5)
7	7.69 (dd, 8.0)	7.68 (dd, 7.1)	7.68 (dd, 7.8)	7.7 (dd, 7.8)	7.70 (dd, 7.9)	7.60 (dd, 7.8)	7.68 (dd, 7.7)
8	7.27 (d, 8.6)	7.23 (d, 8.3)	7.22 (d, 8.3)	7.20 (d, 8.2)	7.30 (d, 8.4)	7.15 (m)	7.27 (d, 8.4)
5'	6.96 (d, 8.8)	7.00 (m)	6.96 (d, 8.3)	6.94 (d, 8.3)	6.99 (m)	6.92 (d, 8.4)	6.95 (m)
6'	7.22 (d, 8.5)	7.27 (d, 8.4)	7.27 (d, 8.4)	7.28 (d, 8.4)	7.23 (d, 8.7)	7.18 (m)	7.22 (d, 8.3)
2''	-	-	-	8.29 (s)	8.37 (s)	-	-
3''	6.76 (s)	6.95 (m)	6.87 (s)	-	-	6.95 (s)	6.91 (s)
8''	6.55 (s)	6.62 (s)	6.59 (s)	6.44 (s)	6.49 (s)	-	6.58 (s)
2'''	7.44 (s)	8.07 (d, 7.4)	8.04 (d, 8.5)	7.35 (d, 8.2)	7.49 (d, 8.4)	8.01 (d, 8.3)	7.58 (m)
3'''	-	7.63 (m)	7.10 (d, 8.5)	6.79 (d, 8.3)	6.99 (m)	7.54 (m)	-
4'''	-	7.52 (m)	-	-	-	7.52 (m)	-
5'''	7.08 (d, 8.6)	7.63 (m)	7.10 (d, 8.5)	6.79 (d, 8.3)	6.99 (m)	7.54 (m)	6.95 (m)
6'''	7.55 (d, 8.6)	8.07 (d, 7.4)	8.04 (d, 8.5)	7.35 (d, 8.2)	7.49 (d, 8.4)	8.01 (d, 8.3)	7.58 (m)
Position	**2**	**6**	**10**	**14**	**18**	**22**	**26**
3	6.18 (s)	6.20 (s)	6.19 (s)	6.18 (s)	6.18 (s)	6.20 (s)	6.18 (s)
5	-	-	-	-	-	-	-
6	6.56 (d, 8.5)	6.68 (m)	6.65 (d, 8.4)	6.66 (d, 8.3)	6.67 (d, 8.3)	6.58 (d, 8.4)	6.70 (d, 8.3)
7	7.58 (m)	7.53 (dd, 8.3)	7.52 (dd, 8.3)	7.55 (dd, 8.3)	7.55 (dd, 8.3)	7.51 (d, 8.3)	7.53 (dd, 8.3)
8	6.70 (d, 8.3)	6.70 (d, 8.1)	6.69 (d, 8.2)	6.72 (d, 8.1)	6.71 (d, 7.9)	6.68 (d, 8.2)	6.65 (d, 8.4)
5'	6.96 (d, 8.5)	6.98 (m)	6.97 (d, 8.4)	6.97 (d, 8.4)	6.96 (d, 7.9)	6.98 (d, 8.3)	6.97 (d, 8.2)
6'	7.24 (d, 8.4)	7.26 (d, 8.3)	7.23 (d, 8.4)	7.25 (d, 8.3)	7.24 (d, 8.3)	7.26 (d, 8.4)	7.25 (d, 8.3)

2''	-	-	-	8.34 (s)	8.37 (s)	-	-
3''	6.76 (s)	6.98 (m)	6.87 (s)	-	-	7.01 (s)	6.92 (s)
8''	6.55 (s)	6.61 (m)	6.60 (s)	6.49 (s)	6.50 (s)	-	6.59 (s,)
2'''	7.44 (s)	8.09 (d, 7.5)	8.04 (d, 8.5)	7.37 (d, 8.2)	7.49 (d, 8.2)	8.02 (d, 7.9)	7.58 (m)
3'''	-	7.66 (m)	7.10 (d, 8.5)	6.81 (d, 8.1)	6.99 (d, 8.4)	7.64 (m)	-
4'''	-	7.56 (m)	-	-	-	7.64 (m)	-
5'''	7.09 (d, 8.6)	7.66 (m)	7.10 (d, 8.5)	6.82 (d, 8.1)	6.99 (d, 8.4)	7.64 (m)	6.95 (d, 8.1)
6'''	7.59 (m)	8.09 (d, 7.5)	8.04 (d, 8.5)	7.38 (d, 8.2)	7.49 (d, 8.2)	8.02 (d, 7.9)	7.58 (m)
Position	**3**	**7**	**11**	**15**	**19**	**23**	**27**
3	6.04 (s)	6.04 (s)	6.02 (s)	6.00 (s)	6.03 (s)	6.01 (s)	6.03 (s)
5	7.14 (m)	7.15 (m)	7.11 (m)	7.16 (m)	7.12 (dd, 8.6, 1.8)	7.07 (m)	7.15 (s)
6	-	-	-	-	-	-	-
7	7.14 (m)	7.20 (m)	7.16 (m)	7.16 (m)	7.21 (m)	7.07 (m)	7.11 (dd, 9.0, 2.9)
8	7.14 (m)	7.20 (m)	7.16 (m)	7.16 (m)	7.21 (m)	7.19 (m)	6.96 (d, 8.3)
5'	6.96 (d, 8.3)	6.95 (d, 8.5)	6.94 (d, 8.3)	6.95 (d, 8.4)	6.94 (d, 8.4)	6.93 (s)	6.94 (d, 8.3)
6'	7.14 (m)	7.09 (m)	7.16 (m)	7.16 (m)	7.21 (m)	7.13 (m)	7.18 (m)
2''	-	-	-	8.32 (s)	8.37 (s)	-	-
3''	6.77 (s)	6.98 (s)	6.87 (s)	-	-	7.00 (s)	6.91 (s)
8''	6.56 (s)	6.61 (s)	6.58 (s)	6.48 (s)	6.48 (s)	-	6.58 (s)
2'''	7.45 (s)	8.08 (d, 7.4)	8.04 (d, 8.6)	7.38 (d, 8.2)	7.49 (d, 8.6)	8.07 (dd, 8.2, 1.7)	7.58 (m)
3'''	-	7.66 (m)	7.11 (m)	6.82 (d, 8.1)	6.99 (d, 8.6)	7.60 (m)	-
4'''	-	7.52 (m)	-	-	-	7.60 (m)	-
5'''	7.10 (d, 8.3)	7.66 (m)	7.11 (m)	6.82 (d, 8.1)	6.99 (d, 8.6)	7.60 (m)	7.18 (m)
6'''	7.56 (d, 8.5)	8.08 (d, 7.4)	8.04 (d, 8.6)	7.38 (d, 8.2)	7.49 (d, 8.6)	8.07 (dd, 8.2, 1.7)	7.58 (m)
Position	**4**	**8**	**12**	**16**	**20**	**24**	**28**
3	6.01 (s)	6.00 (s)	5.99 (s)	5.99 (s)	5.99 (s)	6.02 (s)	5.99 (s)
5	7.75 (d, 8.7)	7.73 (d, 8.4)	7.73 (d, 8.6)	7.75 (d, 8.7)	7.75 (d, 8.7)	7.74 (d, 8.6)	7.74 (d, 8.7)
6	6.81 (dd, 8.7, 2.3)	6.80 (d, 8.1)	6.79 (dd, 8.8, 2.2)	6.83 (d, 8.3)	6.82 (dd, 8.7, 2.3)	6.78 (dd, 8.7, 2.3)	6.80 (dd, 8.7, 2.3)
7	-	-	-	-	-	-	-
8	6.49 (d, 2.2)	6.48 (s)	6.47 (d, 2.2)	6.51 (d, 2.2)	6.52 (d, 2.0)	6.43 (d, 2.2)	6.49 (d, 2.2)
5'	6.95 (d, 8.4)	6.94 (d, 8.1)	6.94 (d, 8.4)	6.94 (d, 8.3)	6.95 (d, 8.3)	6.96 (d, 8.3)	6.96 (m)
6'	7.17 (d, 8.4)	7.16 (d, 8.2)	7.16 (d, 8.4)	7.16 (d, 8.3)	7.17 (d, 8.3)	7.18 (d, 8.4)	7.17 (d, 8.4)
2''	-	-	-	8.32 (s)	8.37 (s)	-	-
3''	6.77 (s)	6.99 (s)	6.94 (s)	-	-	7.03 (s)	6.91 (s)
8''	6.55 (s)	6.60 (s)	6.58 (s)	6.47 (s)	6.49 (s)	-	6.57 (s)

2'''	7.45 (d, 2.3)	8.09 (d, 7.9)	8.05 (d, 8.7)	7.38 (d, 8.5)	7.51 (d, 8.5)	8.09 (dd, 7.8, 1.9)	7.59 (m)
3'''	-	7.60 (m)	7.11 (d, 8.7)	6.81 (d, 8.6)	7.00 (d, 8.5)	7.61 (m)	-
4'''	-	7.60 (m)	-	-	-	7.61 (m)	-
5'''	7.09 (d, 8.6)	7.60 (m)	7.11 (d, 8.7)	6.81 (d, 8.6)	7.00 (d, 8.5)	7.61 (m)	6.96 (m)
6'''	7.56 (dd, 8.5, 2.3)	8.10 (d, 7.9)	8.05 (d, 8.7)	7.38 (d, 8.5)	7.51 (d, 8.5)	8.09 (dd, 7.8, 1.9)	7.59 (m)

Table S2. The ^{13}C chemical shifts of biflavonoids **1-28.**

δ of ^{13}C							
Position	**1**	**5**	**9**	**13**	**17**	**21**	**25**
2	165.68	165.71	165.69	174.89	165.62	165.65	165.72
3	108.17	108.38	108.19	108.52	108.30	108.55	108.15
4	176.55	176.61	176.59	176.63	176.57	176.58	176.59
5	125.20	125.24	125.23	153.82	130.15	125.21	125.20
6	123.00	124.72	124.71	134.05	125.23	124.70	124.71
7	134.00	134.04	134.03	155.64	134.02	133.99	133.99
8	117.79	117.82	117.82	119.90	117.86	117.75	117.81
9	155.72	155.75	155.74	159.30	155.74	155.23	155.75
10	124.69	120.18	122.86	121.33	120.12	120.19	122.99
1'	124.29	122.99	122.97	122.29	122.00	122.96	121.57
2'	118.77	124.32	124.31	148.24	124.71	124.22	124.34
3'	148.13	144.33	144.30	155.77	144.29	144.33	144.36
4'	144.32	148.16	148.13	156.69	148.14	148.19	148.08
5'	114.33	114.41	114.64	117.88	114.36	114.48	114.36
6'	122.96	119.70	119.76	120.11	119.73	119.37	120.14
2''	163.35	163.09	163.20	157.42	154.17	162.85	163.55
3''	103.47	105.23	103.58	122.99	122.97	103.37	103.46
4''	181.74	181.97	181.86	180.27	180.19	182.09	181.87
5''	158.87	158.89	158.87	165.77	159.17	155.68	158.90
6''	108.55	108.61	108.59	114.29	108.56	108.61	108.60
7''	162.12	162.33	162.07	173.62	162.33	154.17	162.08
8''	93.28	93.51	55.60	93.15	93.06	127.48	93.45
9''	156.35	156.53	156.40	163.00	156.64	148.61	156.41
10''	103.52	103.76	93.40	108.38	104.19	105.20	103.29
1'''	120.11	130.73	120.14	124.74	124.26	132.14	120.42
2'''	112.93	126.49	128.41	130.27	130.26	126.32	110.26
3'''	146.83	129.20	114.64	115.10	113.72	129.32	148.17

4'''	151.16	132.07	162.35	165.37	159.28	130.79	150.77
5'''	158.87	129.20	114.37	115.10	113.72	129.32	115.82
6'''	119.76	126.49	128.41	130.27	130.26	126.32	119.86
Position	**2**	**6**	**10**	**14**	**18**	**22**	**26**
2	167.49	167.45	167.45	167.36	167.38	182.17	167.47
3	106.74	106.74	106.76	106.78	106.81	106.79	106.74
4	181.71	181.93	181.85	182.48	182.51	182.61	181.87
5	162.30	162.37	162.05	162.13	162.24	162.98	161.97
6	110.64	110.66	110.67	110.68	110.72	114.59	110.66
7	135.70	135.71	135.72	135.72	135.76	144.51	135.70
8	108.08	108.23	108.04	108.03	108.14	108.50	107.96
9	155.90	155.91	155.92	155.90	155.93	155.93	155.93
10	106.84	106.90	106.91	106.88	106.90	107.00	106.91
1'	120.51	120.57	122.83	120.55	120.57	123.52	121.53
2'	123.54	123.55	123.57	123.51	123.52	127.61	123.58
3'	144.49	144.43	144.41	144.35	144.38	148.75	144.43
4'	146.83	148.71	148.68	148.67	148.69	148.83	148.08
5'	114.30	114.40	114.39	114.40	114.42	119.55	114.37
6'	119.93	119.82	119.88	119.85	119.84	120.72	120.42
2''	163.32	163.05	163.19	153.90	154.22	167.51	163.55
3''	103.51	105.21	103.57	121.24	122.03	103.44	103.46
4''	182.48	182.50	182.50	180.30	180.21	109.71	182.50
5''	158.82	158.82	158.83	159.25	159.19	159.86	158.84
6''	109.63	109.64	109.65	109.64	109.66	110.79	109.64
7''	159.80	159.81	159.81	159.81	159.82	155.33	159.81
8''	93.38	93.55	93.44	93.01	93.08	130.85	93.45
9''	156.40	156.56	156.44	156.66	156.69	154.16	156.43
10''	103.39	103.71	103.52	104.22	104.22	105.27	103.27
1'''	123.00	130.70	120.55	122.32	122.96	135.82	120.54
2'''	112.21	126.47	128.39	130.23	130.27	126.42	110.26
3'''	148.73	129.18	114.63	115.07	113.74	129.45	148.69
4'''	151.15	132.05	162.35	157.42	159.25	132.28	150.77
5'''	112.92	129.18	114.63	115.07	113.74	129.45	115.81
6'''	118.77	126.47	128.39	130.23	130.27	126.42	119.94
Position	**3**	**7**	**11**	**15**	**19**	**23**	**27**
2	165.23	165.20	165.20	165.21	180.24	165.16	165.26
3	107.37	107.35	107.34	107.39	176.54	107.35	107.39
4	176.49	176.46	176.45	176.52	165.22	176.43	176.51
5	108.22	108.40	108.23	114.38	162.32	108.57	110.28

6	154.62	154.61	154.59	153.89	159.30	154.57	154.64
7	119.68	119.59	119.67	119.69	159.22	119.26	120.00
8	118.79	119.12	119.12	119.20	156.66	119.06	119.15
9	151.18	149.45	149.45	149.50	154.66	154.12	150.77
10	122.82	119.98	122.87	121.32	154.21	122.75	122.83
1'	123.03	123.85	123.84	122.36	149.51	123.82	123.88
2'	124.58	124.57	124.58	124.57	147.96	124.46	124.64
3'	144.25	144.24	144.24	144.25	144.26	144.24	144.29
4'	146.85	147.92	147.91	147.95	130.31	147.95	147.94
5'	114.35	114.35	114.31	115.48	130.31	114.42	114.36
6'	119.99	122.80	119.95	120.01	124.57	119.98	120.44
2''	163.39	163.06	163.16	154.65	123.88	162.83	163.58
3''	103.56	105.22	103.57	122.85	123.02	103.32	103.50
4''	181.78	181.94	181.84	180.34	122.87	182.07	181.90
5''	158.86	158.84	158.84	159.31	122.07	155.20	158.90
6''	107.64	107.64	107.63	108.30	120.02	107.63	107.69
7''	162.07	162.31	162.09	162.24	119.67	149.37	161.97
8''	93.25	93.46	93.36	93.00	119.22	127.42	93.40
9''	156.35	156.48	156.36	156.65	114.39	148.56	156.39
10''	103.50	103.72	103.48	104.24	113.77	105.20	103.32
1'''	123.86	130.74	122.79	123.87	113.77	132.13	121.60
2'''	112.24	126.48	128.39	130.29	108.38	126.33	108.18
3'''	147.93	129.19	114.63	115.11	107.66	129.32	148.10
4'''	149.47	132.05	162.33	157.45	107.40	130.81	149.50
5'''	112.95	129.19	114.63	115.11	104.24	129.32	115.84
6'''	119.14	126.48	128.39	130.29	93.06	126.33	119.75

Position	**4**	**8**	**12**	**16**	**20**	**24**	**28**
2	162.42	164.97	162.40	162.45	162.28	165.00	165.03
3	108.16	108.44	108.18	108.20	108.22	108.21	108.20
4	176.08	175.99	176.06	176.09	176.12	176.03	176.06
5	126.38	130.76	126.37	126.41	126.43	127.42	126.37
6	114.77	114.69	114.74	114.80	114.81	114.44	110.25
7	165.07	162.36	165.02	165.00	162.47	162.38	162.40
8	101.95	101.89	101.93	101.98	101.99	101.88	101.95
9	157.55	147.85	157.53	157.56	156.65	155.16	156.36
10	115.81	119.55	114.33	114.36	115.82	115.78	115.81
1'	124.49	124.45	124.48	124.49	124.50	119.99	121.61
2'	118.79	126.32	119.64	119.66	119.64	124.38	124.52
3'	144.29	143.29	144.23	144.23	144.25	144.24	144.25

4'	147.91	144.19	147.86	147.89	147.90	147.92	147.86
5'	112.95	119.15	115.79	115.81	114.39	114.72	114.32
6'	119.99	119.93	119.96	120.01	120.02	119.24	119.97
2''	162.16	162.99	162.34	162.20	154.20	162.83	163.53
3''	108.32	108.13	108.29	121.35	122.08	103.36	103.49
4''	181.80	181.93	181.88	180.36	180.26	182.13	181.91
5''	158.87	156.45	162.03	157.44	159.31	157.45	158.86
6''	103.59	114.33	103.62	104.25	104.25	108.63	108.20
7''	163.36	158.81	163.18	153.88	165.01	154.15	161.91
8''	93.31	93.45	93.37	93.00	93.07	126.36	93.38
9''	156.36	144.21	156.37	156.63	159.22	148.57	157.54
10''	103.49	105.22	103.53	108.32	108.42	105.24	103.33
1'''	123.08	132.01	122.91	122.37	123.06	132.13	120.42
2'''	114.33	126.46	128.41	130.29	130.32	126.33	132.07
3'''	146.86	129.17	114.66	115.11	113.77	129.34	148.09
4'''	151.18	138.48	158.85	159.31	157.58	130.85	150.74
5'''	112.25	129.17	114.66	115.11	113.77	129.34	114.74
6'''	119.69	126.46	128.41	130.29	130.32	126.33	119.70

Table S3. Predicted DPPH activity from field-based QSAR

Compounds	Training or test set	Experimental activity	Predicted activity -1	Predicted activity -2	Predicted activity -3	Predicted activity -4
ZHL1	training	1.167	1.433	1.158	1.142	1.111
ZHL2	training	1.297	1.423	1.426	1.338	1.324
ZHL3	training	1.134	1.056	1.12	1.133	1.134
ZHL4	training	0.853	1.062	1.092	1.076	1.058
ZHL5	test	1.036	1.062	1.092	1.076	1.058
ZHL6	training	1.076	1.41	1.015	1.041	1.045
ZHL7	training	1.141	1.41	1.015	1.041	1.045
ZHL8	training	1.121	1.4	1.283	1.238	1.257
ZHL9	training	1.01	1.033	0.977	1.033	1.067
ZHL10	training	1.082	1.039	0.949	0.976	0.992
ZHL11	training	1.089	1.039	0.949	0.976	0.992
ZHL12	test	1.167	1.412	1.131	1.126	1.128
ZHL13	test	1.03	1.412	1.131	1.126	1.128
ZHL14	training	0.978	1.414	1.041	1.021	1.013
ZHL15	test	0.991	1.043	0.975	0.956	0.959
ZHL16	training	1.236	1.043	0.975	0.956	0.959
ZHL17	training	1.612	1.469	1.373	1.355	1.309
ZHL18	training	1.369	1.459	1.641	1.552	1.522
ZHL19	training	1.338	1.093	1.335	1.347	1.332
ZHL20	training	1.403	1.098	1.307	1.29	1.256
ZHL27	training	1.639	1.471	1.489	1.44	1.393
ZHL28	training	1.5	1.476	1.643	1.563	1.595

ZHL29	training	1.438	1.453	1.5	1.463	1.528
ZHL30	training	1.855	1.457	1.526	1.443	1.496
ZHL35	training	1.82	1.512	1.858	1.777	1.793
ZHL36	training	1.751	1.504	1.61	1.773	1.766
ZHL37	test	1.653	1.481	1.467	1.672	1.699
ZHL38	training	1.833	1.54	1.825	1.986	1.964
